# Feasibility of Immunohistochemical p16 Staining in the Diagnosis of Human Papillomavirus Infection in Patients With Squamous Cell Carcinoma of the Head and Neck: A Systematic Review and Meta-Analysis

**DOI:** 10.3389/fonc.2020.524928

**Published:** 2020-11-25

**Authors:** Huanhuan Wang, Yuyu Zhang, Wei Bai, Bin Wang, Jinlong Wei, Rui Ji, Ying Xin, Lihua Dong, Xin Jiang

**Affiliations:** ^1^Department of Radiation Oncology, The First Hospital of Jilin University, Changchun, China; ^2^Jilin Provincial Key Laboratory of Radiation Oncology & Therapy, The First Hospital of Jilin University, Changchun, China; ^3^NHC Key Laboratory of Radiobiology, School of Public Health, Jilin University, Changchun, China; ^4^Department of Epidemiology and Biostatistics, School of Public Health, Jilin University, Changchun, China; ^5^Department of Biology, Valencia College, Orlando, FL, United States; ^6^Key Laboratory of Pathobiology, Ministry of Education, Jilin University, Changchun, China

**Keywords:** human papillomavirus, squamous cell cancers of the head and neck, immunohistochemical staining, p16, meta-analysis

## Abstract

Human papillomavirus (HPV) is a risk factor for squamous cell carcinoma of the head and neck (HNSCC). This study aimed to investigate the feasibility of IHC- p16INK4a (p16) as an alternative modality for diagnosing HPV infection. We searched PubMed, EMBASE, Web of Science, and Cochrane library for studies that evaluated the diagnostic accuracy of IHC-p16 staining. A total of 30 studies involving 2,963 patients were included from 2007 to 2019. The combined sensitivity was 0.94 (95% CI: 0.92–0.95); specificity, 0.90 (95% CI: 0.89–0.91); positive likelihood ratio (LR), 6.80 (95% CI: 5.63–8.21); negative LR, 0.10 (95% CI: 0.07–0.16); diagnostic odds ratio, 85.98 (95% CI: 55.57–133.03); and area under the curve value, 0.9550. Subgroup analysis showed that the IHC-p16 test was more consistent with the *in situ* hybridization (ISH) test and has greater diagnostic value for oropharyngeal squamous cell carcinoma. The diagnostic efficacy of IHC-p16 varied among countries. In conclusion, IHC-p16 has high sensitivity and specificity for diagnosing HPV infection in HNSCC. The consistency of IHC-p16 findings with those of ISH indicate that their combination can be used to improve the specificity of diagnosis.

## Introduction

Head and neck squamous cell carcinoma (HNSCC) is the sixth most common malignancy worldwide, with ~830,000 incident cases annually (1). Smoking and drinking are the most established risk factors for HNSCC, but ~20–80% of recent HNSCC cases have been reported to be associated with human papillomavirus (HPV) infection (2–4). The proportion of HPV-related tumors vary by country and tumor site (5, 6). In addition, HPV-associated HNSCC has better disease-free survival and overall survival (7–9) owing to its high radiosensitivity (10–12). Concurrently, the standard treatment modality for HNSCC yields more serious adverse reactions, such as dryness of the mouth, dysphagia, and hearing loss, in patients with HPV-related HNSCC patients (4, 7). Accordingly, de-intensified treatment has become the new standard approach for HPV-positive patients. In general, the treatment for patients with HPV-positive tumors is de-intensified to reduce the adverse reactions and improve the quality of life while ensuring good tumor control. This is achieved by reducing the radiation dose and using radiotherapy alone instead of concurrent chemoradiotherapy (13, 14). Correct diagnosis of HPV infection is the most important step in de-escalating treatment. Only when HPV infection is properly diagnosed can a more suitable population be selected for this new treatment approach. However, there are several diagnostic modalities for HPV infection, and they have varying sensitivity and specificity. Therefore, choosing the appropriate modality for accurate diagnosis of HPV infection will be a key challenge for de-escalating treatment.

Currently, HPVE6/E7mRNA detection is the primary basis for diagnosing HPV infection as it has the advantage of detecting HPV with transcriptional activity (9, 15). Common methods for detecting HPVE6/E7mRNA include polymerase chain reaction (PCR) and *in situ* hybridization (ISH). PCR-based detection is more sensitive, while ISH-based detection is more specific (16, 17). However, no specific modality has been recommended as the gold standard for diagnosing HPV infection (18, 19). Both PCR and ISH methods have limitations including stringent sampling requirements, long detection time, complicated detection process, and high cost. Therefore, there have been several efforts to develop a novel diagnostic method for HPV infection that is both simple and economical.

Currently, alternative diagnostic methods include PCR or ISH detection of HPV-DNA (20–22). PCR-DNA is a highly sensitive method that can use primers to detect a wide range of HPV types (23). However, its specificity in distinguishing free and integrated DNA is relatively low. This disadvantage is overcome by ISH that can distinguish between the complete and dissociated form of HPV-DNA according to a dot signal and a diffuse signal (24). Even so, the ISH-DNA test for HPV infection in an integrated state is not reliable, and it is impossible to tell whether HPV-DNA is integrated into the host's genome. Although used clinically, the HPV-DNA test can only reflect a transitory infection and cannot identify the HPV driving the carcinogenic process. Further, its accuracy and prognostic relevance are unclear (25, 26).

Increasing studies have used the immunohistochemical (IHC) p16INK4a(p16) staining as an alternative modality for diagnosing HPV infection (27, 28). In HPV infection of squamous epithelial cells, p16 is overexpressed after infection due to inactivation of the Rb protein (23, 29). In general, upregulated p16 expression is believed to be closely associated with HPV infection (30). As such, this test is widely used in oropharyngeal squamous cell carcinoma (OPSCC); however, the relationship between p16 positivity and HPV infection in non-oropharyngeal sites (e.g., paranasal sinuses, mouth, larynx, nasopharynx and hypopharynx) is extremely limited (31, 32). In addition, the diagnostic efficacy of IHC-p16 for HPV infection in all HNSCC patients has not been completely evaluated. Given the advantages of IHC-p16, including its simple operation, short testing time, and being economical, it is essential to better understand its usefulness in the diagnosis of HPV infection. This systematic review and meta-analysis aimed to investigate the feasibility of using IHC-p16 for diagnosing HPV infection in HNSCC and its value for de-escalating treatment. Further, we aimed to assess whether the results varied by tumor site and country.

## Materials and Methods

### Protocol and Registration

The protocol for this systematic review was registered on INPLASY (202070068) and is available in full on the inplasy.com (https://doi.org/10.37766/inplasy2020.7.0068).

### Search Strategy

This study was conducted according to the PRISMA guidelines and the Cochrane diagnostic test manual (33, 34). The search strategy, study selection, methodological quality assessment, data extraction, and data analysis protocols were developed in advance. We searched PubMed, EMBASE, Web of Science, and Cochrane library for relevant articles published from the establishment of the database until October 2019, without language restrictions. The search was assisted by an experienced library staff member. We used a combination of MeSH words and free text words including “Papillomaviridae” and “Head and Neck Neoplasms.” The search strategy and the number of relevant articles identified in each database are shown in the Supplementary Documents. The references of the identified articles were also reviewed to further search for other relevant articles. All articles were searched according to international standards.

### Study Selection

We reviewed the full text of all observational studies, both retrospective and prospective, and randomized controlled clinical trials that compared the diagnostic efficacy of IHC-p16 positivity with the gold standard modality for HPV diagnosis. The inclusion criteria were: (i) the included patients had HNSCC; (ii) the samples tested were biopsy or puncture specimens; (iii) HPV E6/E7mRNA detection was used as the gold standard for the diagnosis of HPV infection; (iv) p16 expression was detected using IHC; (v) the total sample size was >10. All case reports, preclinical studies, case series, animal studies, and conference summaries were excluded. In addition, papers were also excluded if the specific location of HNSCC was not clearly defined. Further, the included studies must present the specific true positive (TP), false positive (FP), false negative (FN), and true negatives (TN) values or have adequate data so these can be calculated. If data were lacking, we contacted the author by email to ask for the data, and the study was excluded if the author did not respond. Study selection was divided into two parts. First, the authors (JW and BW) screened all the articles independently by browsing the titles and abstracts. Second, the same two authors independently evaluated the full text of the initially included articles. Any disagreements were resolved by the third author (XJ), and the study was finalized for inclusion.

### Methodological Quality Assessment

Two authors (JW and BW) independently assessed the methodological quality of the included studies using the QUADAS-2 tool (35). Briefly, the QUADAS-2 tool comprises four domains, namely, patient selection, index test, reference standard, and flow and timing. In addition, the first three sections are evaluated with respect to clinical applicability. Patient selection primarily evaluates whether the selection of patients have introduced bias, including whether the patient selection is random and whether there is inappropriate exclusion. The index test primarily evaluates whether the conduct or interpretation of the test has bias, including whether the process of the experiment is detailed. The reference standard evaluates biases caused by reference criteria and their interpretations. The flow and timing evaluates whether all patients are using the same criteria. Evaluation of the these four parameters helps to assess the risk of bias.

### Data Extraction

Data extraction was carried out in two parts. First, a researcher (HHW) used a pre-designed data extraction table to extract basic elements from the study, such as author, publication year, and patient source. Then, two other authors (YYZ and WB) independently extracted the specific values of TP, FP, FN, and TN from the text and cross-checked them according to the pre-set standards to ensure the accuracy of the original values extracted. Any differences in the data extraction were resolved through discussion and negotiation. The extracted data were verified by the third author (WB).

### Data Analysis

Because the head and neck are divided into many regions, and there are two methods for HPVE6/E7mRNA detection, we expected that the data included in the meta-analysis might be uneven. Therefore, we divided the study into several different subgroups based on factors such as tumor location and the detection methods for HPVE6/E7mRNA set as the gold standard. Given that the accuracy of p16 positivity in diagnosing HPV infection is related to the positive threshold, differences in thresholds between studies may have an impact on the sensitivity and specificity. Thus, we further evaluated whether there was a threshold effect using Spearman correlation coefficient. If there was no threshold effect, the sensitivity, specificity, and other indicators were further combined. Sensitivity was defined as the percentage of TP for diagnosing HPV infection in the total number of p16-positive cases (TP+FN). Specificity was defined as the percentage of TN for diagnosis of no-HPV infection in the total number of p16-negative cases (FP+TN). All data were combined using Meta Disc and STATA 15.0 software. We developed a forest map that graphically displays estimates of sensitivity and specificity and visualized heterogeneity between studies. Moreover, heterogeneity was examined using *I*^2^ and Cochrane *Q*-tests. An *I*^2^ of >50% indicated heterogeneity, and the source of heterogeneity was further explored. After obtaining the sensitivity and specificity values, we further used the receiver operating characteristic curve (ROC) model to obtain the positive likelihood ratio (LR), negative LR, and the diagnostic odds ratio (OR) and their 95% confidence intervals (CI). Positive LR was defined as the ratio of sensitivity to 1-specificity. Negative LR was defined as the ratio of 1-sensitivity to specificity. The larger the positive LR and the smaller the negative LR, the better the diagnostic experiment. The diagnostic OR was defined as the ratio of positive LR to negative LR. The greater the diagnostic OR, the better the capability of p16 to distinguish between HPV infection and non-HPV infection. The ROC curve was also drawn to obtain the area under the curve (AUC) value to comprehensively evaluate the efficacy of p16 positivity in diagnosing HPV infection. In addition, a funnel plot was used to further evaluate the presence of publication bias.

## Results

**Figure 1:**
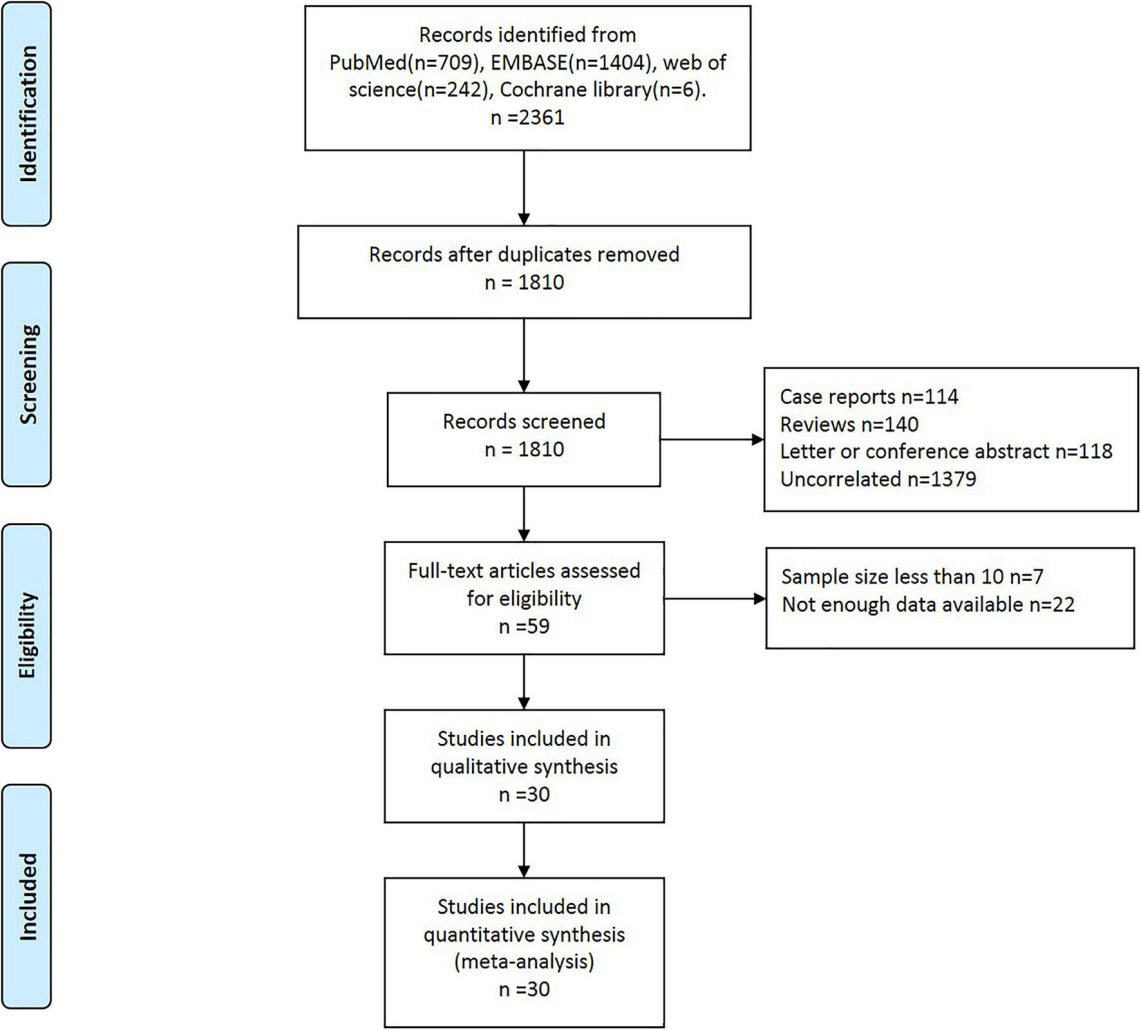
Flow diagram of article search and study selection.

In total, 2,361 studies were initially identified (Figure 1). After excluding 550 duplicate studies, 1,810 studies were screened, and the full text of 59 studies were reviewed. Of the 59 studies, 29 studies were excluded because the sample size was too small (*n* = 7) and detailed data were not available (*n* = 22). Finally, 30 studies (15, 36–64) involving 2,963 patients were included in the meta-analysis.

The characteristics of the 30 studies are shown in Table 1. Some studies used two methods simultaneously. In the 19 (54.3%) studies, HPVE6/E7mRNA was detected using ISH as the gold standard for the diagnosis of HPV infection. Meanwhile, in the 16 (45.7%) studies, HPVE6/E7mRNA was detected via PCR as the gold standard for HPV infection. In total, 5 studies used both ISH and PCR as the gold standard for diagnosis. A total of 2,014 (68.0%) OPSCC cases were included from 25 studies. Other studies included cases with malignant tumors in other subregions of the head and neck, including 752 (25.4%) cases of oral squamous cell carcinoma, 148 (5.0%) cases of hypopharyngeal squamous cell carcinoma, and 49 (1.6%) cases of nasopharyngeal squamous cell carcinoma. With respect to the country of origin, 1,008 (34.0%) cases were from Europe, 1,592 (53.7%) cases from North America, 283 (9.6%) cases from Asia, and 80 (2.7%) cases from Oceania. All the included studies were published after 2007.

**Table 1:** Characteristics of all include studies (*n* = 2,963).

**Authors**	**Country**	**Year**	***n***	**Age**	**Method**	**Region**	**TP**	**FP**	**FN**	**TN**
Smeets et al. (36)	NL	2007	29	–	PCR	OC	6	4	0	19
Smeets et al. (36)	NL	2007	15	–	PCR	OP	5	3	0	7
Shi et al. (37)	CA	2009	111	58.7	PCR	OP	62	10	11	28
Hoffmann et al. (38)	DE	2010	39	62.1	PCR	OP	10	2	1	26
Rotnaglova et al. (39)	CZK	2010	45	–	PCR	OP	26	1	1	17
Schache et al. (40)	UK	2011	95	58.5	PCR	OP	32	11	2	50
Ukpo et al. (41)	USA	2011	192	56.2	ISH	OP	148	3	4	37
Bishop et al. (42)	USA	2012	77	–	ISH	OP	42	6	1	28
Bishop et al. (42)	USA	2012	109	–	ISH	OC	1	8	0	100
Jordan et al. (15)	USA	2012	232	–	PCR	OP	153	12	5	62
Mehrad et al. (43)	USA	2013	18	61.5	ISH	OP	13	0	1	4
Mehrad et al. (43)	USA	2013	19	58.9	ISH	LP	2	0	3	14
Dreyer et al. (44)	DE	2013	64	66	ISH	OP	18	3	0	43
Gao et al. (45)	USA	2013	98	56.1	PCR	OP	74	2	4	18
Lingen et al. (46)	USA	2013	409	–	PCR	OC	19	27	5	358
Deng et al. (47)	JP	2014	53	64.1	PCR	OP	17	3	1	32
Mehrad et al. (48)	USA	2014	20	–	PCR	OP	19	0	0	19
Poling et al. (49)	USA	2014	78	55	ISH	OC	1	8	0	69
Salazar et al. (50)	USA	2014	50	–	PCR	OP	22	4	1	23
Jalaly et al. (51)	USA	2015	27	60.8	ISH	OP	18	0	1	8
Kerr et al. (52)	USA	2015	34	–	ISH	OP	29	1	0	4
Laco et al. (53)	CZK	2015	49	62	ISH	NP	13	3	0	33
Laco et al. (53)	CZK	2015	48	62	PCR	NP	8	6	0	32
Mirghani et al. (54)	FR	2015	44	–	PCR	OP	26	2	1	15
Morbini et al. (55)	IT	2015	41	63.68	ISH	OP	20	2	0	19
Young et al. (56)	AU	2015	80	66	ISH	LP	7	0	0	73
Bhosale, et al. (57)	IN	2016	49	52	ISH	LP	1	0	2	46
Bhosale et al. (57)	IN	2016	54	52	ISH	OP	3	1	2	48
Mirghani et al. (58)	FR	2016	104	56	ISH	OP	59	11	3	31
Gelwan et al. (59)	USA	2017	32	61	ISH	OP	1	1	0	30
Minami et al. (60)	JP	2017	127	63.8	PCR	OC	3	15	4	105
Augustin et al. (61)	FR	2018	126	63.2	ISH	OP	42	11	9	64
Chernesky et al. (62)	CA	2018	59	59.8	PCR	OP	46	2	1	10
Drumheller et al. (63)	USA	2019	27	–	ISH	OP	21	0	3	3
Randén-Brady et al. (64)	FI	2019	357	–	ISH	OP	211	15	10	121

*All included articles are sorted by year of publication. The following is the full abbreviation of the table. NL, Nederland; CA, Canada; DE, Germany; CZ, Crzech Republic; UK, The United Kingdom; JP, Japan; USA, United states of America; FR, French; IT, Italy; AU, Australia; IN, India; FI, Finland; PCR, Polymerase chain reaction; ISH, In situ hybridization; OP, Oropharyngeal; OC, oral cavity; LP, larynx; NP, Nasopharyngeal*.

### Methodological Quality

**Figure 2:**
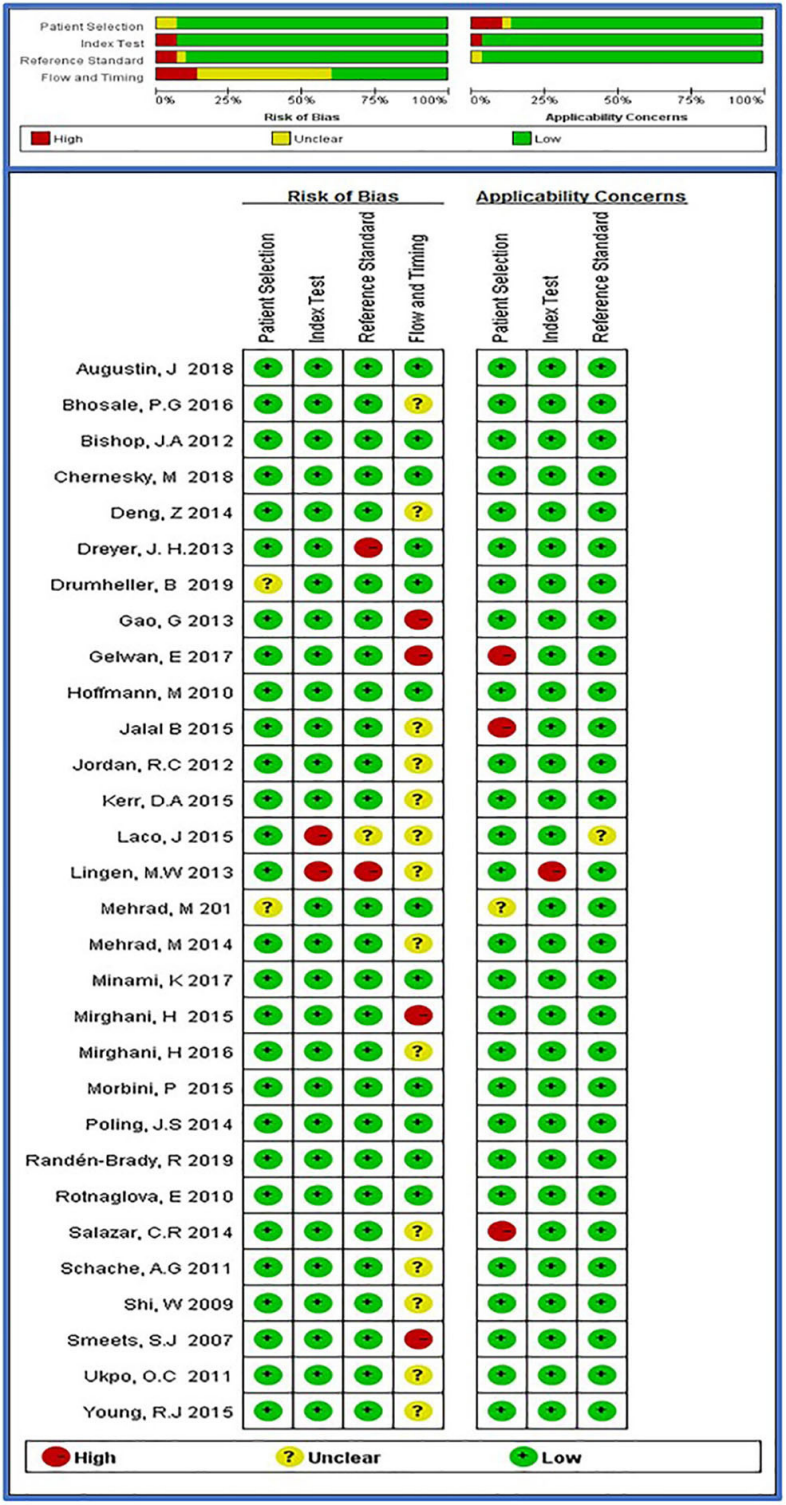
Summary of bias risks and applicability concerns for each study, based on QUADAS-2.

The results of quality assessment of the 30 studies are shown in Figure 2. In many studies, not all the factors that might influence the quality assessment were completely reported. With respect to patient selection, 2 studies were assessed to have uncertain risk of bias mainly because patient selection was unclear. For flow and timing, 14 studies were assessed to have uncertain risk of bias mainly because the time interval between tests was not specified. A total of 7 studies were assessed to have high risk of bias in different areas mainly because not all cases were included in the analysis.

### Diagnostic Efficacy of p16 Positivity

**Figure 3:**
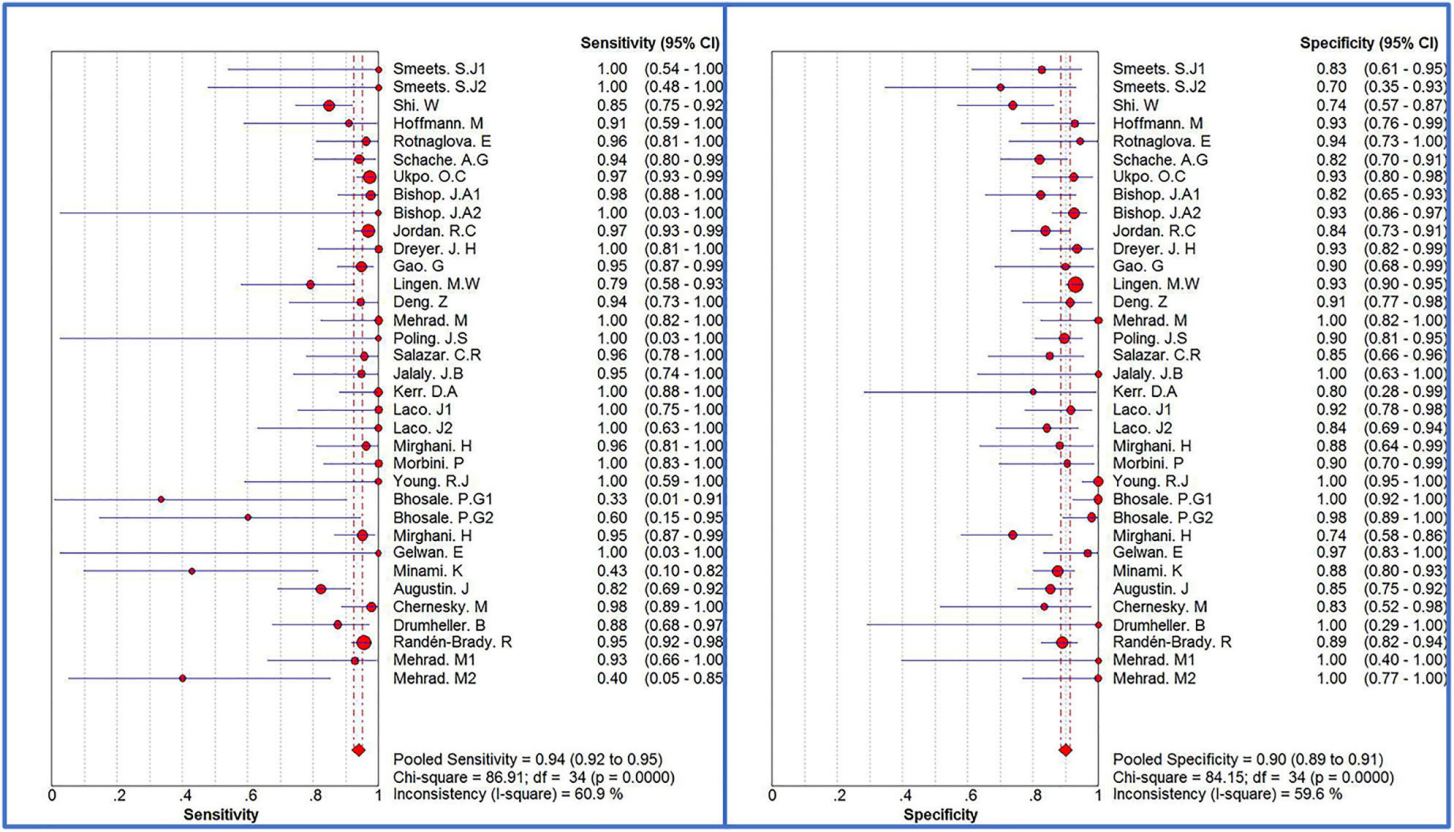
Forest plots of sensitivity and specificity of 30 original studies combined for diagnosis of HPV infection compared with p16 positive. Among them, 5 studies used different detection methods or included patients with different tumors, so they were divided into two records, named 1.2, respectively.

**Figure 4:**
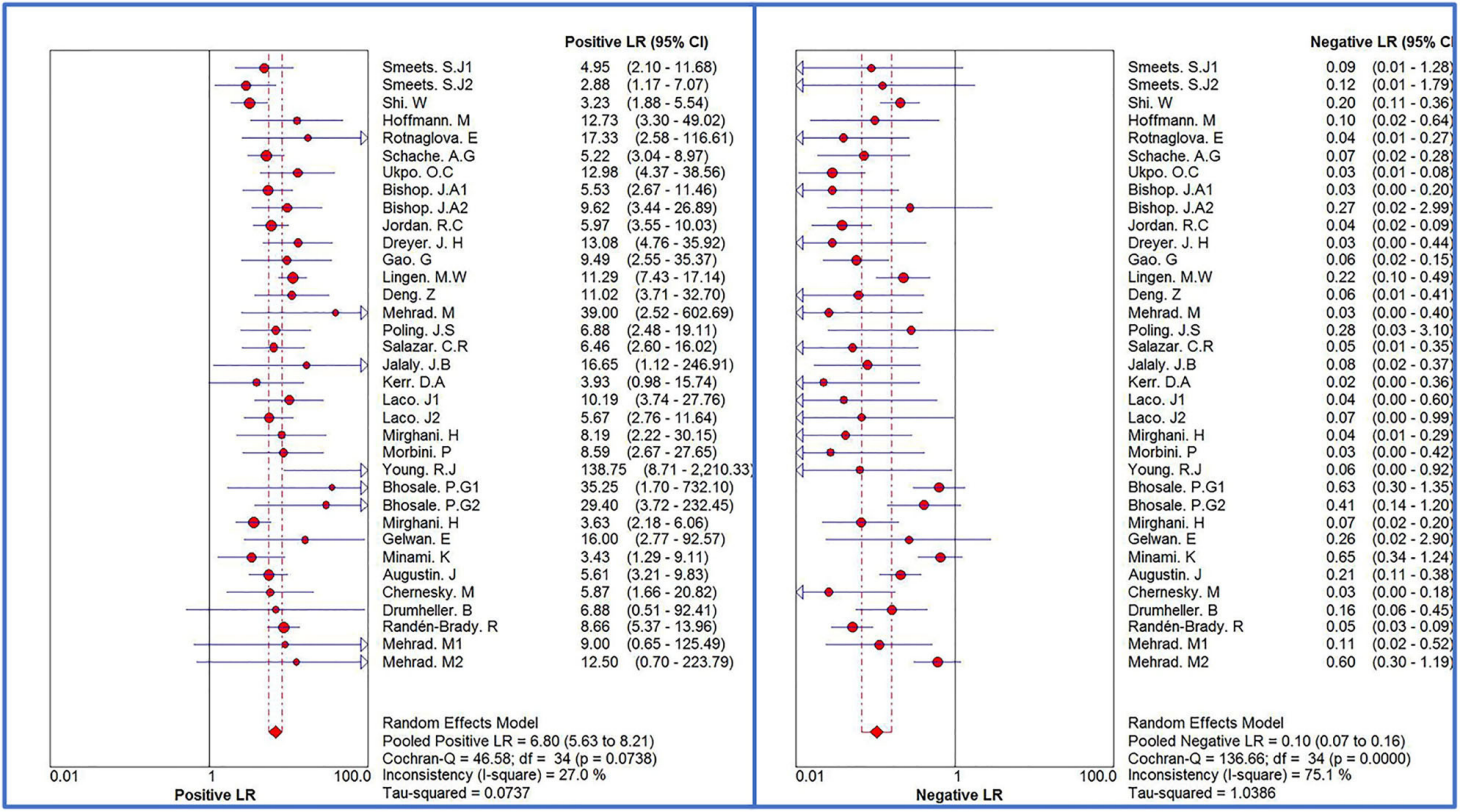
Forest plots of positive LR and negative LR of 30 original studies combined for diagnosis of HPV infection compared with p16 positive. Among them, 5 studies used different detection methods or included patients with different tumors, so they were divided into two records, named 1.2, respectively.

**Figure 5:**
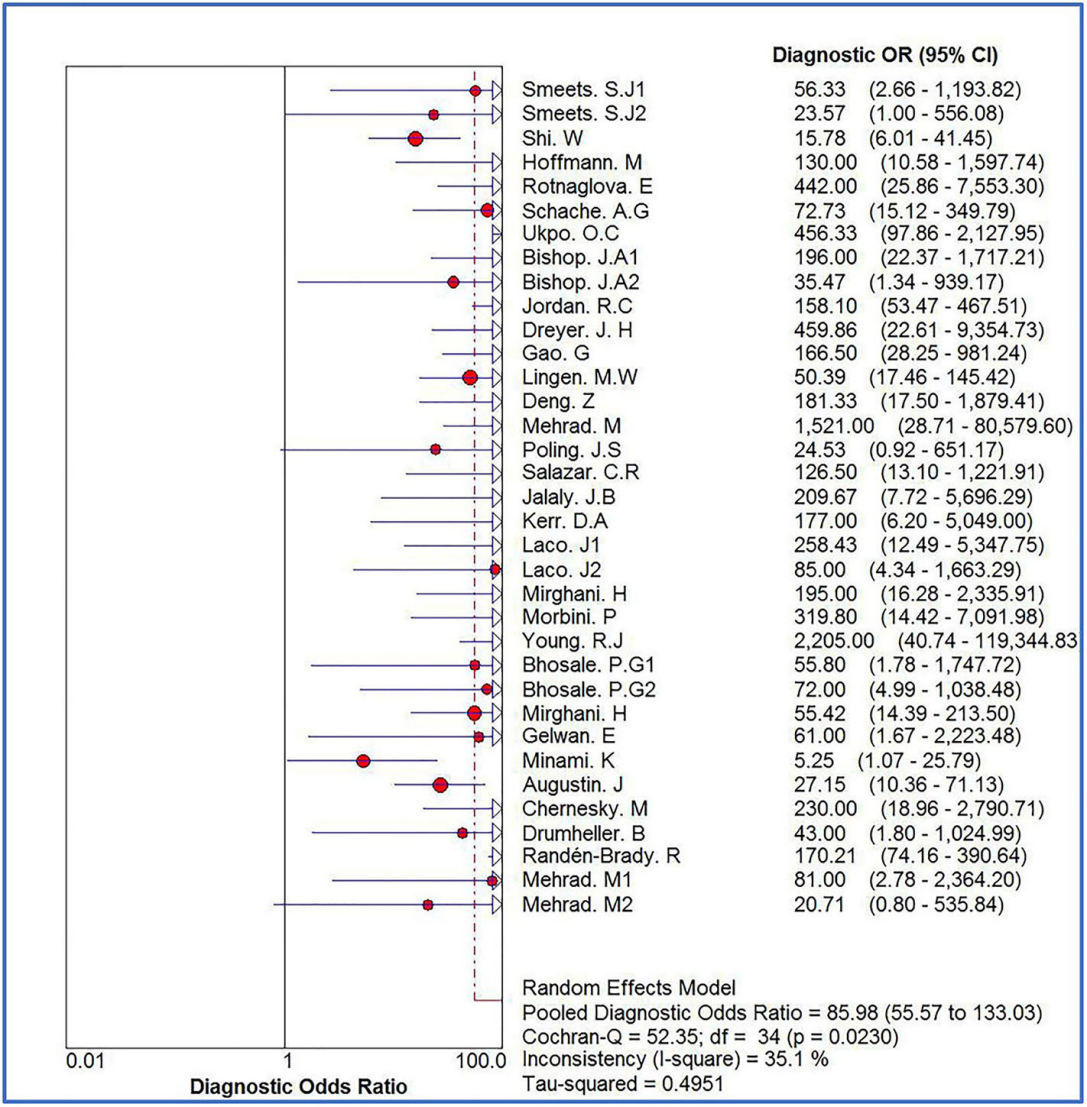
Forest plot of diagnostic OR of 30 original studies combined for diagnosis of HPV infection compared with p16 positive. Heterogeneity test *I*^2^ = 35.1%.

**Figure 6:**
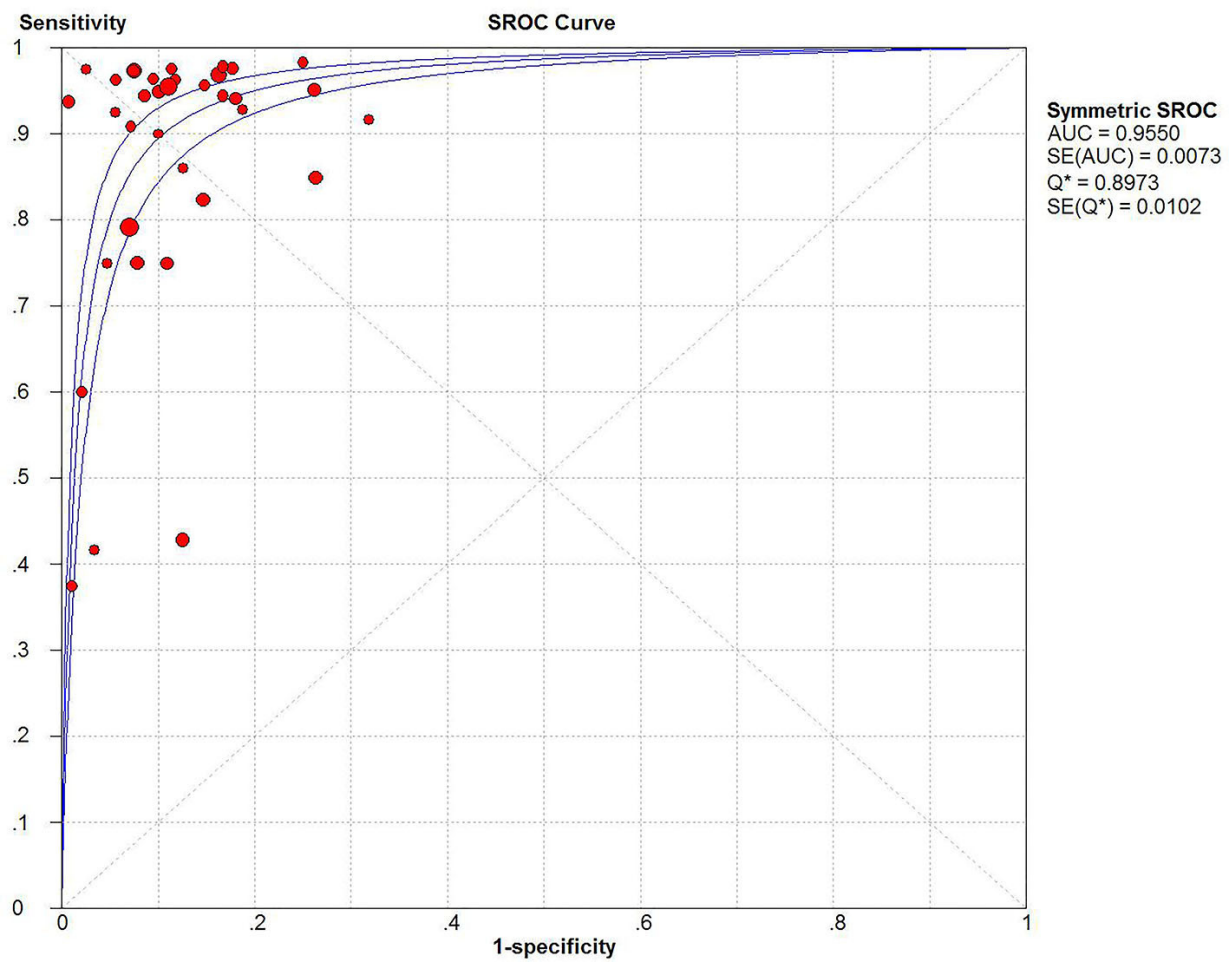
SROC curve and 95% CI of 30 studies combined for diagnosis of HPV infection compared with p16 positive, AUC value = 0.9550.

The Spearman correlation coefficient was *p* = 0.081, indicating that there was no threshold effect in this meta-analysis. Therefore, we further combined the sensitivity, specificity, and other indicators of the study. The combined sensitivity of the 30 studies was 0.94 (95% CI: 0.92–0.95) and specificity was 0.90 (95% CI: 0.89–0.91) (Figure 3). We found that p16 positivity had high sensitivity for diagnosing HPV infection. Among the p16-positive patients, 94% had HPV infection; the misdiagnosis rate was only 6%. Among the p-16 negative patients, 90% were not infected with HPV, but there was a 10% of missed diagnosis. The positive LR was 6.80 (95% CI: 5.63–8.21); negative LR, 0.10 (95% CI: 0.07–0.16) (Figure 4); and diagnostic OR, 85.98 (95% CI: 55.57–133.03). These values indicate that p16 positivity was able to distinguish 85.98% of HPV infections from non-infections. The *I*^2^-value was 35.1%, indicating good consistency (Figure 5), and the AUC value was 0.9550 (Figure 6), showing that p16 positivity has great diagnostic value.

In addition, we investigated whether the diagnostic efficacy of p16 positivity is consistent when different testing methods are used as the gold standard. PCR and ISH were analyzed separately. For ISH HPVE6/E7mRNA, the combined sensitivity was 0.94 (95% CI: 0.92–0.96); specificity, 0.91 (95% CI: 0.89–0.93); positive LR, 7.53 (95% CI: 5.77–9.83); and negative LR, 0.12 (95% CI: 0.06–0.22). The diagnostic OR was 101.09 (95% CI: 59.93–170.54); *I*^2^-value, 12.1%; and AUC value, 0.9627. For PCR HPVE6/E7mRNA, the combined sensitivity was 0.93 (95% CI: 0.91–0.95); specificity, 0.89 (95% CI: 0.87–0.91); positive LR, 6.17 (95% CI: 4.70–8.09); negative LR, 0.09 (95% CI: 0.05–0.17); diagnostic OR, 75.25 (95% CI: 38.28–147.91); *I*^2^-value, 48.9%; and AUC value, 0.9424. After all the studies were grouped according to the test methods, the possibility of heterogeneity in each group was reduced. When ISH was used as the gold standard, the combined sensitivity, specificity, and diagnostic OR were higher, and the AUC value was larger than those in the PCR. This indicates that when ISH is used as the gold standard, p16 has higher diagnostic efficacy for HPV infection. The combined results are shown in Table 2.

**Table 2:** Summary of combined effect values after grouping according to gold standard detection method, tumor location and study country.

**Subgroups**	***N* (%)**	**Sensitivity (95% CI)**	**Specificity (95% CI)**	**Positive LR (95% CI)**	**Negative LR 95% CI)**	**Diagnostic OR (95% CI)**	**AUC value**	***I*^**2**^**
ISH	1537 (51.05%)	0.95 (0.92–0.96)	0.91 (0.89–0.93)	7.53 (5.77–9.83)	0.12 (0.06–0.22)	101.09 (59.93–170.54)	0.9627	12.10%
PCR	1474 (48.95%)	0.93 (0.91–0.95)	0.89 (0.87–0.91)	6.17 (4.70–8.09)	0.09 (0.05–0.17)	75.25 (38.28–147.9)	0.9424	48.90%
OPSCC	2014 (67.97%)	0.95 (0.93–0.96)	0.88 (0.85–0.90)	6.33 (5.12–7.83)	0.08 (0.05–0.11)	104.49 (65.14–167.61)	0.9598	31.90%
Non-OPSCC	949 (32.03%)	0.79 (0.77–0.88)	0.93 (0.91–0.94)	8.43 (5.46–13.01)	0.33 (0.17–0.66)	40.38 (14.36–113.49)	0.9455	33.90%
Europe	1008 (34.02%)	0.95 (0.92–0.96)	0.87 (0.84–0.90)	6.30 (4.74–8.38)	0.08 (0.05–0.12)	90.94 (50.89–162.49)	0.9515	18.20%
America	1592 (53.73%)	0.94 (0.92–0.96)	0.91 (0.89–0.92)	7.06 (5.43–9.18)	0.10 (0.05–0.17)	91.91 (48.84–172.97)	0.9552	34.4%
Non-western	363 (12.25%)	0.94 (0.93–0.95)	0.94 (0.91–0.96)	14.77 (4.27–51.09)	0.33 (0.13–0.88)	68.96 (9.07–524.47)	0.9667	66.50%

*In the meta-analysis, 48 samples were diagnosed with HPV infection using both test methods as gold standard. When the detection method was used for subgroup analysis, the two groups of data were classified as different subgroups for analysis. In the grouping of tumor regions and study countries, data analyzed by PCR were deleted to avoid duplication*.

To further explore whether the diagnostic efficacy of p16 positivity for HPV infection is consistent across all sites of squamous cell carcinomas, we conducted subgroup analysis according to tumor site. In patients with OPSCC, the combined sensitivity was 0.95 (95% CI: 0.93–0.96); specificity, 0.88 (95% CI: 0.85–0.90); positive LR, 6.33 (95% CI: 5.12–7.83); negative LR, 0.08 (95% CI: 0.05–0.11); diagnostic OR, 104.49 (95% CI: 65.14–167.61); *I*^2^-value, 31.9%; and AUC value, 0.9598. In non-OPSCC patients, the combined sensitivity was 0.79 (95% CI: 0.77–0.88); specificity, 0.93 (95% CI: 0.91–0.94); positive LR, 8.43 (95% CI: 5.46–13.01); negative LR, 0.33 (95% CI: 0.17–0.66); diagnostic OR, 40.38 (95% CI: 14.36–113.49); *I*^2^-value, 33.9%; and AUC value, 0.9455. Compared with non-OPSCC patients, the sensitivity, positive LR, diagnostic OR, and AUC value of HPV infection diagnosed according to p16 positivity is higher in OPSCC patients, indicating that p16 positivity has higher diagnostic efficacy for HPV infection in OPSCC (Table 2).

In subgroup analysis by country to investigate whether the diagnostic efficacy of p16 positivity for HPV infection is consistent across countries, we grouped studies according to their origin: European, north American, and non-Western. The results showed higher diagnostic efficacy of p16 positivity in European and American countries. In contrast, in non-western countries, the combined diagnostic OR was only 68.96, and heterogeneity was observed, indicating that p16 positivity had no significant diagnostic value. The above results are shown in Table 2. The forest plots for all subgroups are shown in the Supplementary Document.

**Figure 7:**
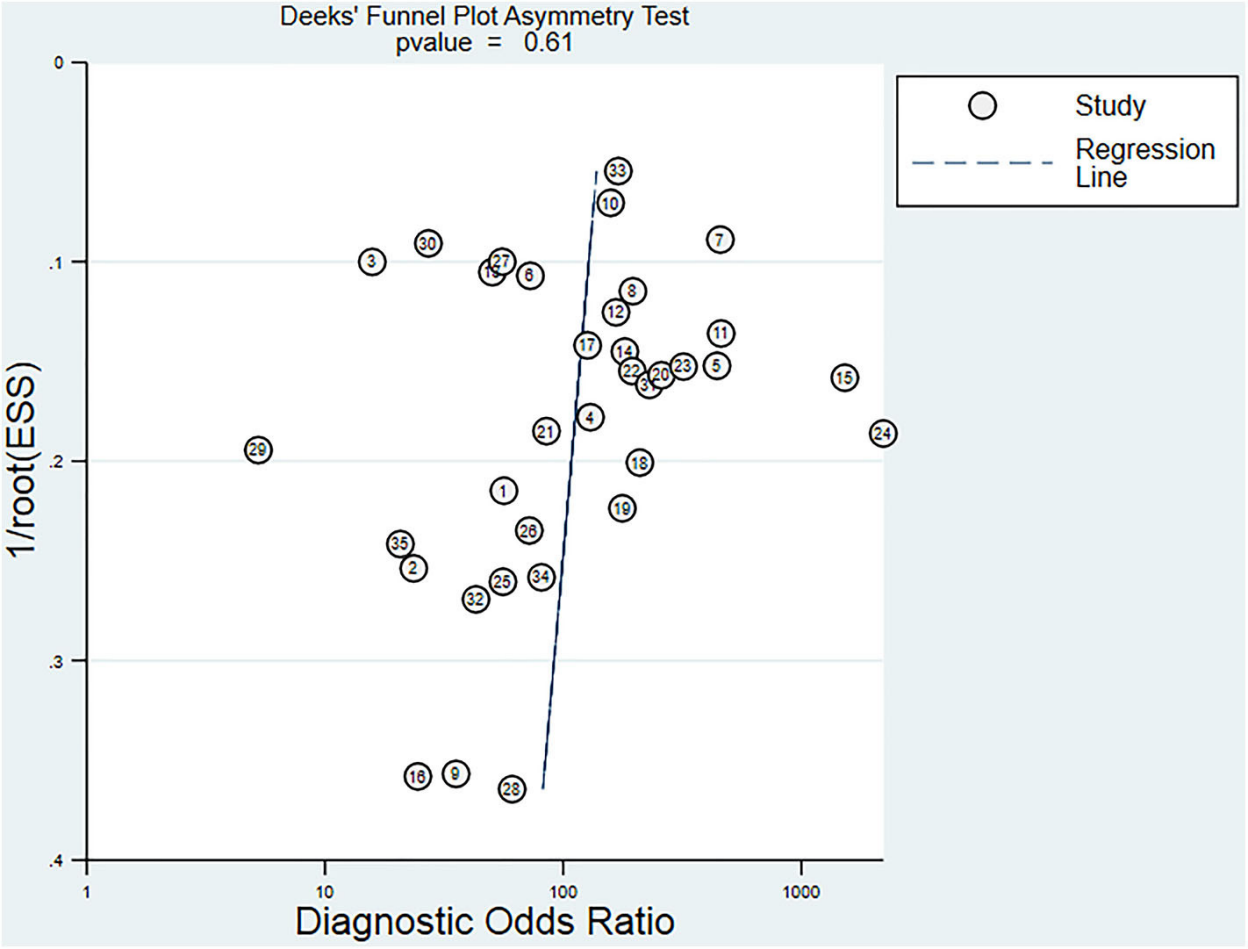
Funnel plot of publication bias, *p* = 0.61, it can be considered without publication bias.

The funnel plot for publication bias showed no statistically significant difference (*p* = 0.61), indicating that there was no publication bias (Figure 7).

## Discussion

This systematic review and meta-analysis investigated the diagnostic accuracy of p16 for HPV infection. We found that p16 expression has high sensitivity and moderate specificity as an alternative biomarker for the diagnosis of HPV infection. The findings of this meta-analysis are consistent with those of previous studies where p16 expression for diagnosing HPV infection had 90% sensitivity and >80% specificity (27, 28, 65). Concurrently, the misdiagnosis rate was 5–20%. This suggests that p16 alone has inadequate diagnostic efficacy for HPV infection (41, 66, 67). In some cases of HPVE6/E7mRNA-negative HNSCC, p16 staining was still diffuse, indicating that p16 expression was not specific to HPV infection (36, 68). High expression of p16 was also found in cervical adenocarcinoma, suggesting that the high expression of p16 can be carried out in a non-HPV dependent manner (69). Some researchers also highlighted that p16 overexpression may be related to Rb dysfunction, but Rb dysfunction may not be related to HPV infection (70). Rb protein is the upstream protein of p16, and its mutation can lead to up-regulation of p16 expression (71), and the false-positive rate is ~25% (72). Therefore, the overexpression of upstream protein and gene mutation of p16 may also be important causes of p16 upregulation. In addition, IHC-p16 was performed in only one section of the tumor tissue. The staining results may vary between sections, leading to incorrect results. In addition, the cut-off value for p16 positivity also widely varied between studies, ranging from 5 to 75%. There also many terms used for its definition, such as diffusion and powerful staining, which are unspecific (36, 73). Therefore, different diagnostic cut-off value, different staining levels, and the subjectivity of the diagnoser may lead to partial negative results, which may be the important reasons for false negative errors. It is also important that mutations and deletions in the p16 gene itself prevent it from being overexpressed in a HPV-dependent manner. The correlation between p16 and HPV infection may differ according to different patterns. In general, p16 positivity is not completely indicative of HPV infection. Aside from IHC-p16 being a simple and more readily available method, it also costs 2–6 times lower than other detection methods and has high sensitivity. However, its specificity is relatively moderate (73). Therefore, the clinical use of p16 in the diagnosis of HPV infection should be fully considered. When considering de-escalating treatment, the diagnosis of HPV infection should be more specific. Therefore, p16 alone may not be the optimal biomarker. A recent meta-analysis showed that the combination of IHC-p16 with HPV-DNA testing significantly improved the specificity of the diagnosis of HPV infection (74). To overcome the limitations of a single detection method, a novel strategy of using a combination of different detection methods for HPV is proposed (75). The combination of IHC-p16 with other HPV-specific tests may be more appropriate for selecting patients eligible for de-escalated treatment (75).

The studies in this meta-analysis used different modalities as the gold standard for diagnosing HPV infection. The results showed that the diagnostic efficacy of IHC-p16 for detecting HPVE6/E7mRNA differed between PCR and ISH, with IHC-p16 being more consistent with ISH for diagnosing HPV infection. PCR is widely used because of its high sensitivity and specificity (19), but PCR detection usually requires a higher level of skills and special experimental conditions to avoid contamination. Further, it is more difficult to replicate clinically. Meanwhile, ISH has higher specificity (19), but it has lower sensitivity in cases of low viral load. Thus, the selection of the appropriate diagnostic modality should be individualized, placing high importance on reducing the misdiagnosis when considering de-escalating treatment for HPV-positive HNSCC patients. Lower misdiagnosis rates can prevent the wrongful treatment de-escalation for HPV-negative patients, which can lead to poor local control of the tumor. Compared with missed diagnosis of HPV infection, the consequences of misdiagnosis are more fatal. Considering the current incidence of HPV-related HNSCC and the socio-economic cost of various test methods, the Supplementary Diagnostic modality should be a more practical strategy based on p16 positivity. Accordingly, ISH-HPVE6/E7mRNA should be evaluated in p16-positive tumors to improve the specificity of detection and prevent unreasonable de-escalation of treatment.

In subgroup analysis according to tumor location, p16 positivity for HPV diagnosis had higher sensitivity and specificity in OPSCC. The diagnostic OR was also two times higher than that of non-OPSCC. This may be related to the different infection rates of HPV in different tumor sites (5). Accordingly, p16 expression has been reported to have diagnostic value in OPSCC. Previous studies have also reported that the positive predictive value of p16 expression is lower for tumors outside the oropharynx, suggesting that IHC-p16 should not be used as an alternative biomarker for non-OPSCC (76, 77). Collectively, these findings indicate that IHC-p16 should be used cautiously in the diagnosis of HPV infection in non-OPSCC.

In subgroup analysis according to country, we found that the diagnostic efficacy of p16 expression varied between countries. This difference may be related to the different infection rates of HPV, which is influenced by alcohol and tobacco smoking and sexual behavior. Collectively, these results suggested the optimal diagnostic biomarker for HPV infection may different by country or region.

This meta-analysis was conducted according to stringent guidelines. Relevant studies were identified from four databases using a pre-defined search strategy, and data were extracted according to pre-set tables. Further, the risk of bias for each study was analyzed, and the data were analyzed statistically using two software. However, this study also has some limitations, including the lack of prospective data and multivariate analysis.

## Conclusion

IHC-p16 staining is a highly effective modality for diagnosing HPV infection, particularly for OPSCC patients. However, the diagnostic efficacy varies between countries, and misdiagnosis could not be eliminated. When selecting patients for treatment de-escalation, HPVE6/E7mRNA should be detected using ISH based on p16 positivity to ensure accurate treatment.

## Data Availability Statement

All datasets generated for this study are included in the article/Supplementary Material.

## Author Contributions

XJ and LD: conceptualization and validation. BW: software. WB: formal analysis. HW: investigation. YZ: resources. HW, BW, and YZ: writing-original draft preparation. RJ, YX, and XJ: writing-review and editing. XJ: funding acquisition. All authors read and approved the manuscript.

## Conflict of Interest

The authors declare that the research was conducted in the absence of any commercial or financial relationships that could be construed as a potential conflict of interest.
