# Supplementary material for: Feasibility of Immunohistochemical p16 Staining in the Diagnosis of Human Papillomavirus Infection in Patients With Squamous Cell Carcinoma of the Head and Neck: A Systematic Review and Meta-Analysis
Source: Front Oncol. 2020 Nov 25;10:524928. doi: 10.3389/fonc.2020.524928 (PMC7724109; doi:10.3389/fonc.2020.524928)
Supplement: Supplementary file 1 [file Data_Sheet_1.zip › Supplement file 2020.1.7/Supplementary documents - search strategy in PubMed.docx]

| Recent queries in pubmed | | |
| --- | --- | --- |
| Search | Query | Items found |
| #28 | Search (((((((((((((((((((((((((((Neoplasms, Head[Title/Abstract] AND Neck[Title/Abstract])) OR Head, Neck Neoplasms[Title/Abstract]) OR (Cancer of Head[Title/Abstract] AND Neck[Title/Abstract])) OR (Head[Title/Abstract] AND Neck Cancer[Title/Abstract])) OR (Cancer of the Head[Title/Abstract] AND Neck[Title/Abstract])) OR Upper Aerodigestive Tract Neoplasms[Title/Abstract]) OR UADT Neoplasms[Title/Abstract]) OR Neoplasm, UADT[Title/Abstract]) OR Neoplasms, UADT[Title/Abstract]) OR UADT Neoplasm[Title/Abstract]) OR Neoplasms, Upper Aerodigestive Tract[Title/Abstract]) OR Head Neoplasms[Title/Abstract]) OR Neoplasms, Head[Title/Abstract]) OR Neck Neoplasms[Title/Abstract]) OR Neoplasms, Neck[Title/Abstract]) OR Cancer of Head[Title/Abstract]) OR Head Cancer[Title/Abstract]) OR Cancer of the Head[Title/Abstract]) OR Cancer of Neck[Title/Abstract]) OR Neck Cancer[Title/Abstract]) OR Cancer of the Neck[Title/Abstract])) OR ("Head and Neck Neoplasms"[Mesh]))) AND (("Papillomaviridae"[Mesh]) OR (((((((((((((Human Papilloma Virus[Title/Abstract]) OR Human Papilloma Viruses[Title/Abstract]) OR Papilloma Virus, Human[Title/Abstract]) OR Papilloma Viruses, Human[Title/Abstract]) OR Virus, Human Papilloma[Title/Abstract]) OR Viruses, Human Papilloma[Title/Abstract]) OR HPV, Human Papillomavirus Viruses[Title/Abstract]) OR Human Papillomavirus Viruses[Title/Abstract]) OR Human Papillomavirus Virus[Title/Abstract]) OR Papillomavirus Virus, Human[Title/Abstract]) OR Papillomavirus Viruses, Human[Title/Abstract]) OR Virus, Human Papillomavirus[Title/Abstract]) OR Viruses, Human Papillomavirus[Title/Abstract])))) AND ((((((((((((((((((((((((((((((Immunolabeling Techniques[Title/Abstract]) OR Immunolabeling Technique[Title/Abstract]) OR Technique, Immunolabeling[Title/Abstract]) OR Techniques, Immunolabeling[Title/Abstract]) OR Immunolabeling Technics[Title/Abstract]) OR Immunolabeling Technic[Title/Abstract]) OR Technic, Immunolabeling[Title/Abstract]) OR Technics, Immunolabeling[Title/Abstract]) OR Immunogold Techniques[Title/Abstract]) OR Immunogold Technique[Title/Abstract]) OR Technique, Immunogold[Title/Abstract]) OR Techniques, Immunogold[Title/Abstract]) OR Immunogold Technics[Title/Abstract]) OR Immunogold Technic[Title/Abstract]) OR Technic, Immunogold[Title/Abstract]) OR Technics, Immunogold[Title/Abstract]) OR Immunohistocytochemistry[Title/Abstract]) OR Immunogold-Silver Techniques[Title/Abstract]) OR Immunogold Silver Techniques[Title/Abstract]) OR Immunogold-Silver Technique[Title/Abstract]) OR Technique, Immunogold-Silver[Title/Abstract]) OR Techniques, Immunogold-Silver[Title/Abstract]) OR Immunogold-Silver Technics[Title/Abstract]) OR Immunogold Silver Technics[Title/Abstract]) OR Immunogold-Silver Technic[Title/Abstract]) OR Technic, Immunogold-Silver[Title/Abstract]) OR Technics, Immunogold-Silver[Title/Abstract]) OR Immunocytochemistry[Title/Abstract])) OR "Immunohistochemistry"[Mesh]) | 709 |
| #27 | Search (((((((((((((((((((((((((((((Immunolabeling Techniques[Title/Abstract]) OR Immunolabeling Technique[Title/Abstract]) OR Technique, Immunolabeling[Title/Abstract]) OR Techniques, Immunolabeling[Title/Abstract]) OR Immunolabeling Technics[Title/Abstract]) OR Immunolabeling Technic[Title/Abstract]) OR Technic, Immunolabeling[Title/Abstract]) OR Technics, Immunolabeling[Title/Abstract]) OR Immunogold Techniques[Title/Abstract]) OR Immunogold Technique[Title/Abstract]) OR Technique, Immunogold[Title/Abstract]) OR Techniques, Immunogold[Title/Abstract]) OR Immunogold Technics[Title/Abstract]) OR Immunogold Technic[Title/Abstract]) OR Technic, Immunogold[Title/Abstract]) OR Technics, Immunogold[Title/Abstract]) OR Immunohistocytochemistry[Title/Abstract]) OR Immunogold-Silver Techniques[Title/Abstract]) OR Immunogold Silver Techniques[Title/Abstract]) OR Immunogold-Silver Technique[Title/Abstract]) OR Technique, Immunogold-Silver[Title/Abstract]) OR Techniques, Immunogold-Silver[Title/Abstract]) OR Immunogold-Silver Technics[Title/Abstract]) OR Immunogold Silver Technics[Title/Abstract]) OR Immunogold-Silver Technic[Title/Abstract]) OR Technic, Immunogold-Silver[Title/Abstract]) OR Technics, Immunogold-Silver[Title/Abstract]) OR Immunocytochemistry[Title/Abstract])) OR "Immunohistochemistry"[Mesh] | 609510 |
| #25 | Search (((((((((((((((((((((((((((Immunolabeling Techniques[Title/Abstract]) OR Immunolabeling Technique[Title/Abstract]) OR Technique, Immunolabeling[Title/Abstract]) OR Techniques, Immunolabeling[Title/Abstract]) OR Immunolabeling Technics[Title/Abstract]) OR Immunolabeling Technic[Title/Abstract]) OR Technic, Immunolabeling[Title/Abstract]) OR Technics, Immunolabeling[Title/Abstract]) OR Immunogold Techniques[Title/Abstract]) OR Immunogold Technique[Title/Abstract]) OR Technique, Immunogold[Title/Abstract]) OR Techniques, Immunogold[Title/Abstract]) OR Immunogold Technics[Title/Abstract]) OR Immunogold Technic[Title/Abstract]) OR Technic, Immunogold[Title/Abstract]) OR Technics, Immunogold[Title/Abstract]) OR Immunohistocytochemistry[Title/Abstract]) OR Immunogold-Silver Techniques[Title/Abstract]) OR Immunogold Silver Techniques[Title/Abstract]) OR Immunogold-Silver Technique[Title/Abstract]) OR Technique, Immunogold-Silver[Title/Abstract]) OR Techniques, Immunogold-Silver[Title/Abstract]) OR Immunogold-Silver Technics[Title/Abstract]) OR Immunogold Silver Technics[Title/Abstract]) OR Immunogold-Silver Technic[Title/Abstract]) OR Technic, Immunogold-Silver[Title/Abstract]) OR Technics, Immunogold-Silver[Title/Abstract]) OR Immunocytochemistry[Title/Abstract] | 41100 |
| #24 | Search "Immunohistochemistry"[Mesh] | 589679 |
| #18 | Search (((((((((((((((((((((((((Neoplasms, Head[Title/Abstract] AND Neck[Title/Abstract])) OR Head, Neck Neoplasms[Title/Abstract]) OR (Cancer of Head[Title/Abstract] AND Neck[Title/Abstract])) OR (Head[Title/Abstract] AND Neck Cancer[Title/Abstract])) OR (Cancer of the Head[Title/Abstract] AND Neck[Title/Abstract])) OR Upper Aerodigestive Tract Neoplasms[Title/Abstract]) OR UADT Neoplasms[Title/Abstract]) OR Neoplasm, UADT[Title/Abstract]) OR Neoplasms, UADT[Title/Abstract]) OR UADT Neoplasm[Title/Abstract]) OR Neoplasms, Upper Aerodigestive Tract[Title/Abstract]) OR Head Neoplasms[Title/Abstract]) OR Neoplasms, Head[Title/Abstract]) OR Neck Neoplasms[Title/Abstract]) OR Neoplasms, Neck[Title/Abstract]) OR Cancer of Head[Title/Abstract]) OR Head Cancer[Title/Abstract]) OR Cancer of the Head[Title/Abstract]) OR Cancer of Neck[Title/Abstract]) OR Neck Cancer[Title/Abstract]) OR Cancer of the Neck[Title/Abstract])) OR ("Head and Neck Neoplasms"[Mesh]))) AND (("Papillomaviridae"[Mesh]) OR (((((((((((((Human Papilloma Virus[Title/Abstract]) OR Human Papilloma Viruses[Title/Abstract]) OR Papilloma Virus, Human[Title/Abstract]) OR Papilloma Viruses, Human[Title/Abstract]) OR Virus, Human Papilloma[Title/Abstract]) OR Viruses, Human Papilloma[Title/Abstract]) OR HPV, Human Papillomavirus Viruses[Title/Abstract]) OR Human Papillomavirus Viruses[Title/Abstract]) OR Human Papillomavirus Virus[Title/Abstract]) OR Papillomavirus Virus, Human[Title/Abstract]) OR Papillomavirus Viruses, Human[Title/Abstract]) OR Virus, Human Papillomavirus[Title/Abstract]) OR Viruses, Human Papillomavirus[Title/Abstract])) | 5115 |
| #17 | Search (((((((((((((((((((((((Neoplasms, Head[Title/Abstract] AND Neck[Title/Abstract])) OR Head, Neck Neoplasms[Title/Abstract]) OR (Cancer of Head[Title/Abstract] AND Neck[Title/Abstract])) OR (Head[Title/Abstract] AND Neck Cancer[Title/Abstract])) OR (Cancer of the Head[Title/Abstract] AND Neck[Title/Abstract])) OR Upper Aerodigestive Tract Neoplasms[Title/Abstract]) OR UADT Neoplasms[Title/Abstract]) OR Neoplasm, UADT[Title/Abstract]) OR Neoplasms, UADT[Title/Abstract]) OR UADT Neoplasm[Title/Abstract]) OR Neoplasms, Upper Aerodigestive Tract[Title/Abstract]) OR Head Neoplasms[Title/Abstract]) OR Neoplasms, Head[Title/Abstract]) OR Neck Neoplasms[Title/Abstract]) OR Neoplasms, Neck[Title/Abstract]) OR Cancer of Head[Title/Abstract]) OR Head Cancer[Title/Abstract]) OR Cancer of the Head[Title/Abstract]) OR Cancer of Neck[Title/Abstract]) OR Neck Cancer[Title/Abstract]) OR Cancer of the Neck[Title/Abstract])) OR ("Head and Neck Neoplasms"[Mesh]) | 335695 |
| #16 | Search (((((((((((((((((((((Neoplasms, Head[Title/Abstract] AND Neck[Title/Abstract])) OR Head, Neck Neoplasms[Title/Abstract]) OR (Cancer of Head[Title/Abstract] AND Neck[Title/Abstract])) OR (Head[Title/Abstract] AND Neck Cancer[Title/Abstract])) OR (Cancer of the Head[Title/Abstract] AND Neck[Title/Abstract])) OR Upper Aerodigestive Tract Neoplasms[Title/Abstract]) OR UADT Neoplasms[Title/Abstract]) OR Neoplasm, UADT[Title/Abstract]) OR Neoplasms, UADT[Title/Abstract]) OR UADT Neoplasm[Title/Abstract]) OR Neoplasms, Upper Aerodigestive Tract[Title/Abstract]) OR Head Neoplasms[Title/Abstract]) OR Neoplasms, Head[Title/Abstract]) OR Neck Neoplasms[Title/Abstract]) OR Neoplasms, Neck[Title/Abstract]) OR Cancer of Head[Title/Abstract]) OR Head Cancer[Title/Abstract]) OR Cancer of the Head[Title/Abstract]) OR Cancer of Neck[Title/Abstract]) OR Neck Cancer[Title/Abstract]) OR Cancer of the Neck[Title/Abstract] | 99370 |
| #15 | Search "Head and Neck Neoplasms"[Mesh] | 300335 |
| #12 | Search ("Papillomaviridae"[Mesh]) OR (((((((((((((Human Papilloma Virus[Title/Abstract]) OR Human Papilloma Viruses[Title/Abstract]) OR Papilloma Virus, Human[Title/Abstract]) OR Papilloma Viruses, Human[Title/Abstract]) OR Virus, Human Papilloma[Title/Abstract]) OR Viruses, Human Papilloma[Title/Abstract]) OR HPV, Human Papillomavirus Viruses[Title/Abstract]) OR Human Papillomavirus Viruses[Title/Abstract]) OR Human Papillomavirus Virus[Title/Abstract]) OR Papillomavirus Virus, Human[Title/Abstract]) OR Papillomavirus Viruses, Human[Title/Abstract]) OR Virus, Human Papillomavirus[Title/Abstract]) OR Viruses, Human Papillomavirus[Title/Abstract]) | 38488 |
| #11 | Search ((((((((((((Human Papilloma Virus[Title/Abstract]) OR Human Papilloma Viruses[Title/Abstract]) OR Papilloma Virus, Human[Title/Abstract]) OR Papilloma Viruses, Human[Title/Abstract]) OR Virus, Human Papilloma[Title/Abstract]) OR Viruses, Human Papilloma[Title/Abstract]) OR HPV, Human Papillomavirus Viruses[Title/Abstract]) OR Human Papillomavirus Viruses[Title/Abstract]) OR Human Papillomavirus Virus[Title/Abstract]) OR Papillomavirus Virus, Human[Title/Abstract]) OR Papillomavirus Viruses, Human[Title/Abstract]) OR Virus, Human Papillomavirus[Title/Abstract]) OR Viruses, Human Papillomavirus[Title/Abstract] | 31127 |
| #10 | Search "Papillomaviridae"[Mesh] | 31579 |
