# Supplementary figures and images for: Feasibility of Immunohistochemical p16 Staining in the Diagnosis of Human Papillomavirus Infection in Patients With Squamous Cell Carcinoma of the Head and Neck: A Systematic Review and Meta-Analysis
Source: Front Oncol. 2020 Nov 25;10:524928. doi: 10.3389/fonc.2020.524928 (PMC7724109; doi:10.3389/fonc.2020.524928)

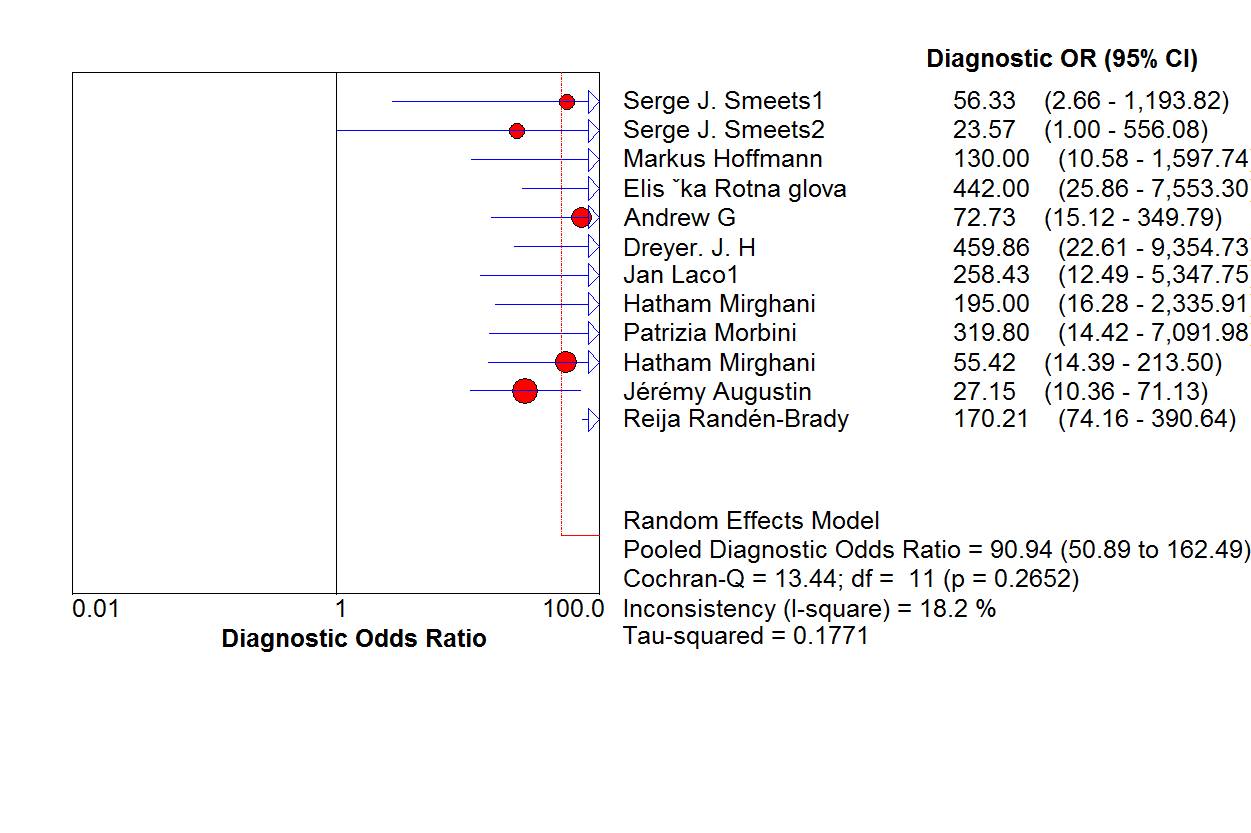

Supplement: Supplementary file 1 [file Data_Sheet_1.zip › Supplement file 2020.1.7/Supplementary file - figure/different countries/European-diagnostic OR.jpg]

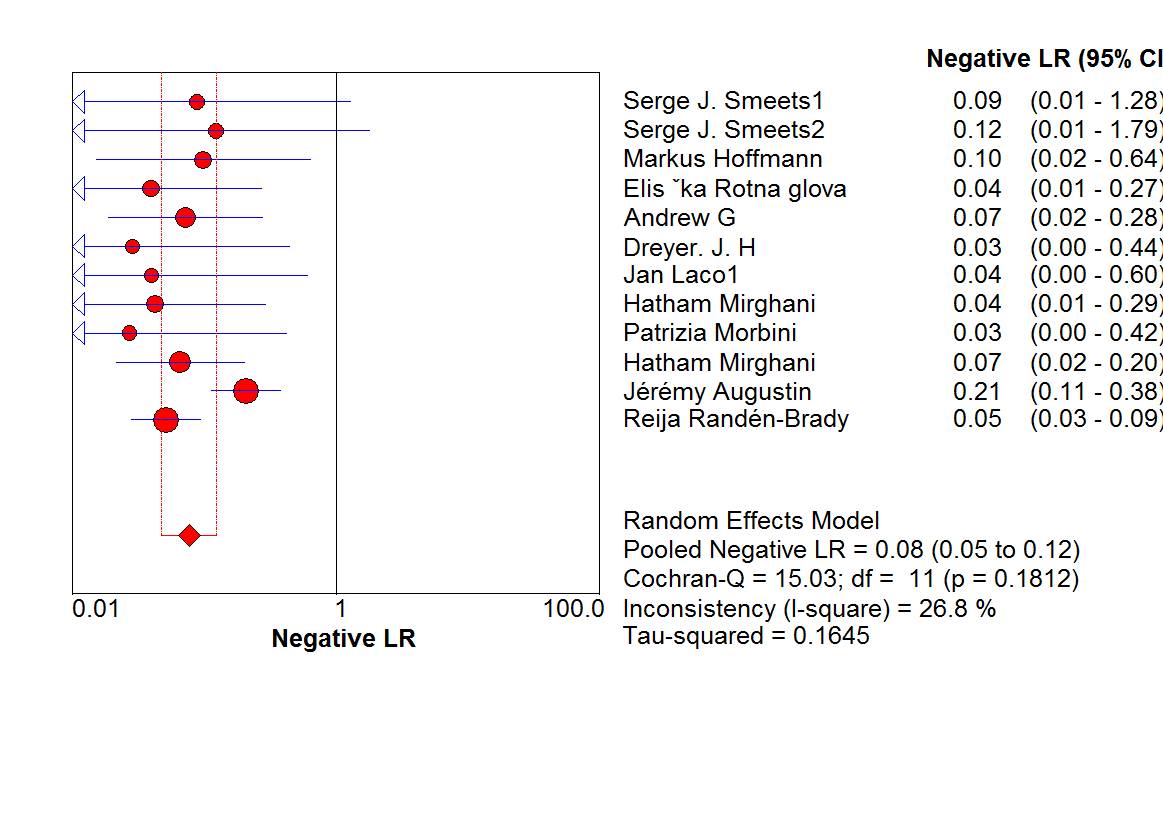

Supplement: Supplementary file 1 [file Data_Sheet_1.zip › Supplement file 2020.1.7/Supplementary file - figure/different countries/European-negative LR.jpg]

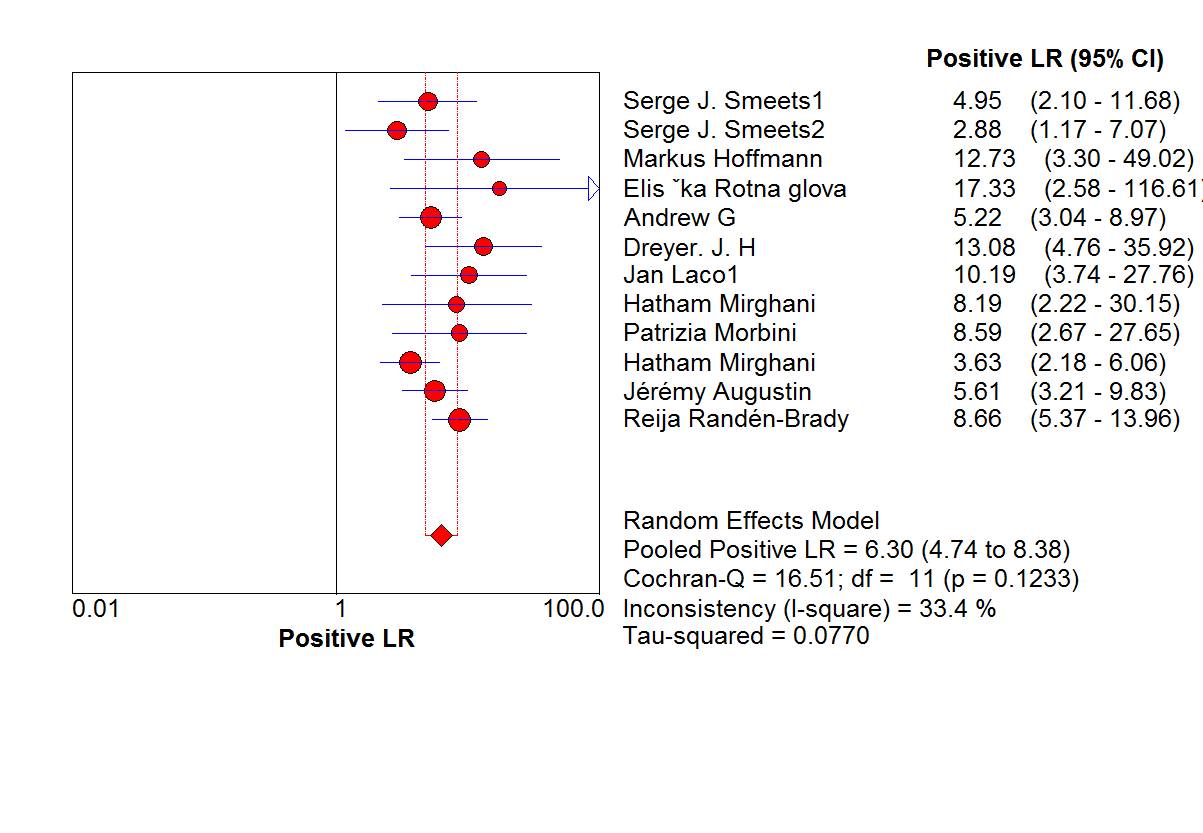

Supplement: Supplementary file 1 [file Data_Sheet_1.zip › Supplement file 2020.1.7/Supplementary file - figure/different countries/European-positive LR.jpg]

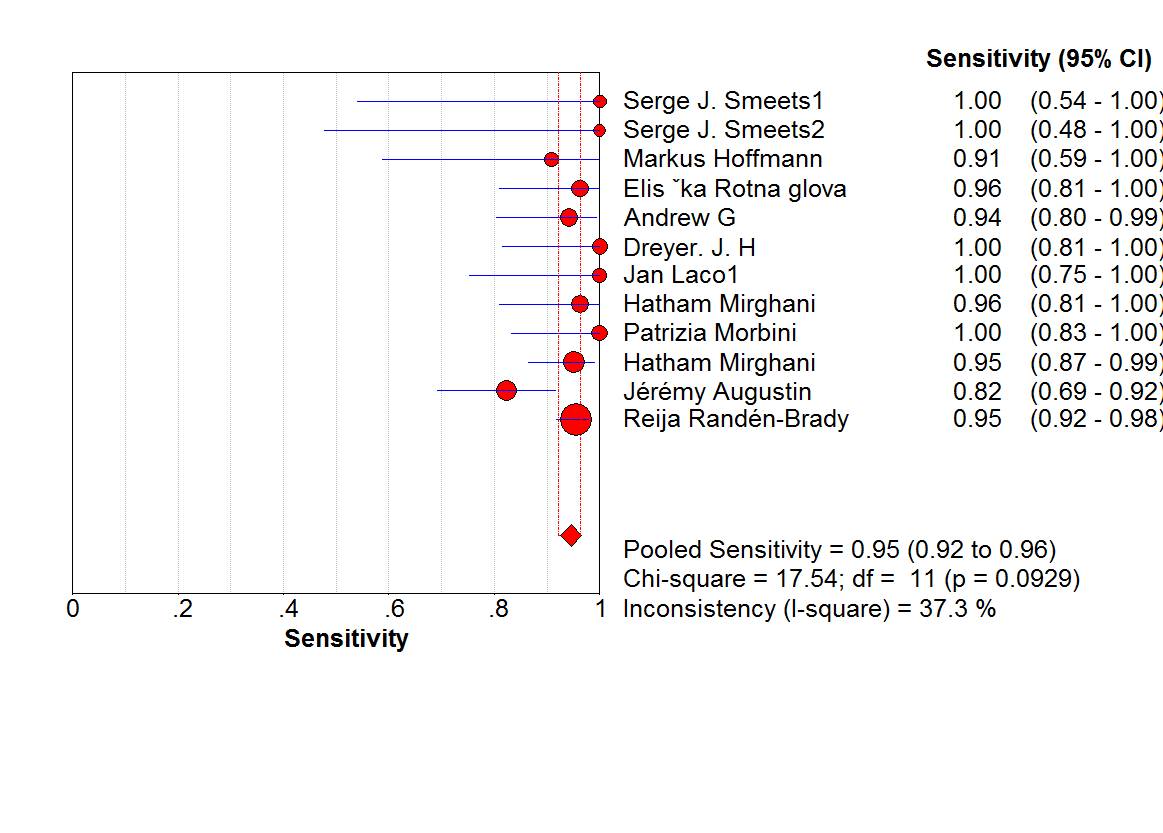

Supplement: Supplementary file 1 [file Data_Sheet_1.zip › Supplement file 2020.1.7/Supplementary file - figure/different countries/European-sensitivity.jpg]

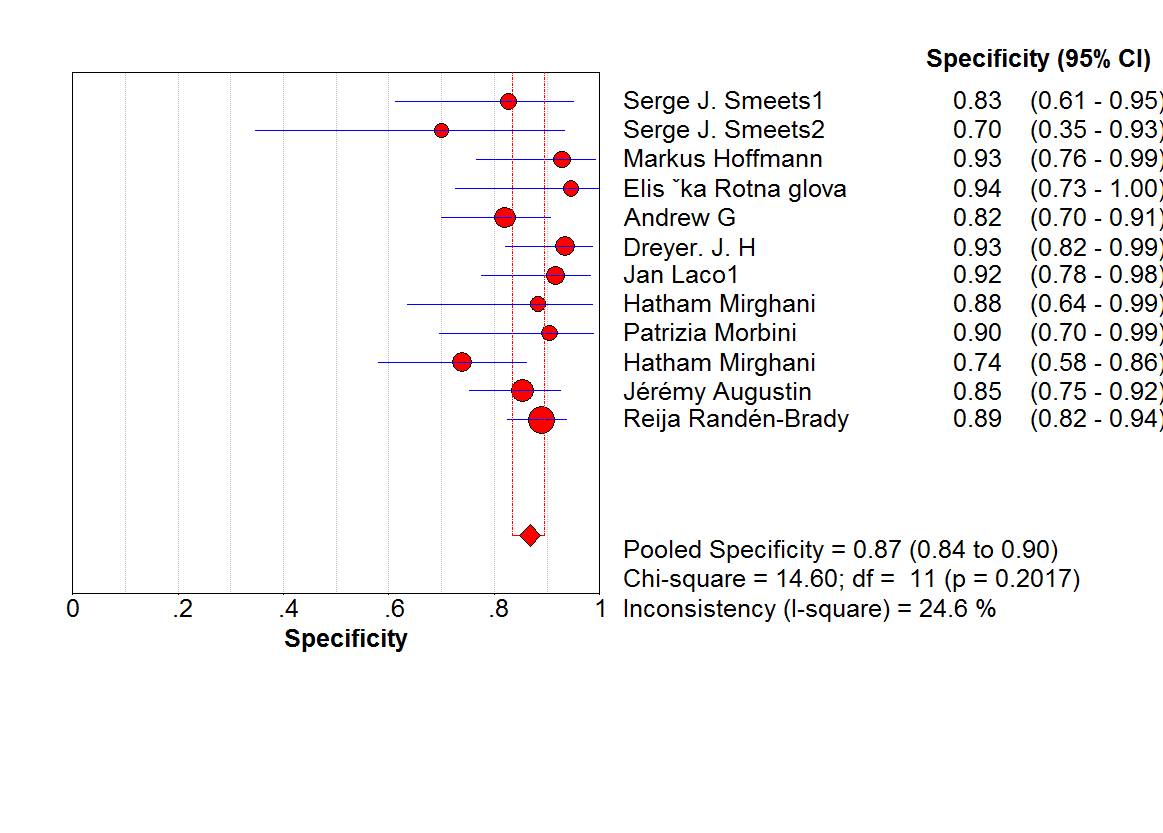

Supplement: Supplementary file 1 [file Data_Sheet_1.zip › Supplement file 2020.1.7/Supplementary file - figure/different countries/European-specificity.jpg]

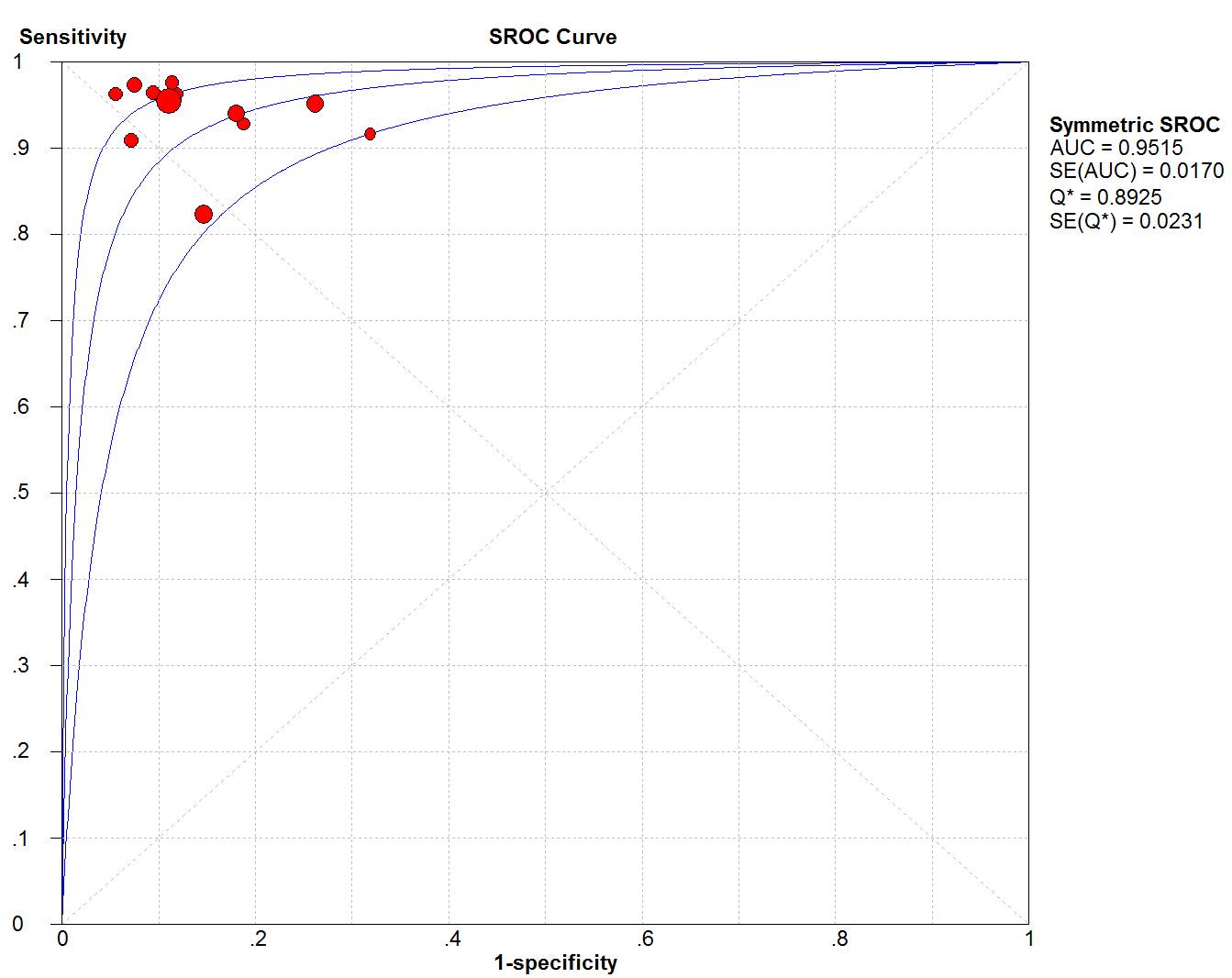

Supplement: Supplementary file 1 [file Data_Sheet_1.zip › Supplement file 2020.1.7/Supplementary file - figure/different countries/European-SROC curve.jpg]

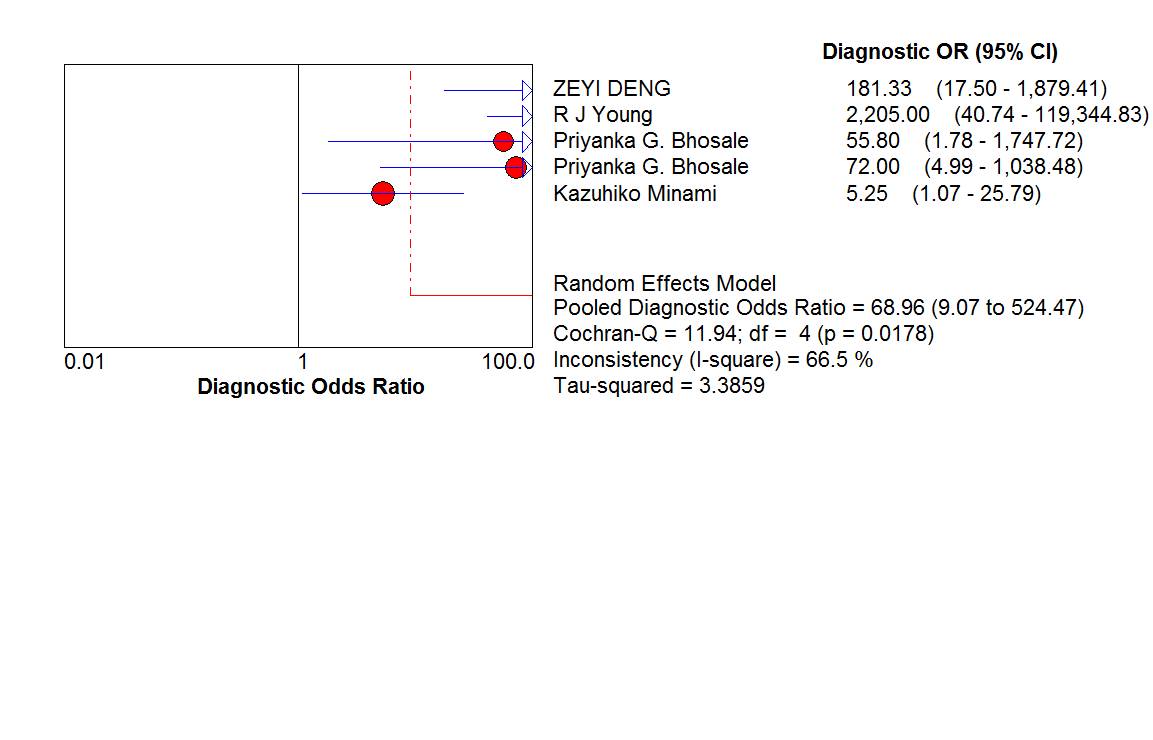

Supplement: Supplementary file 1 [file Data_Sheet_1.zip › Supplement file 2020.1.7/Supplementary file - figure/different countries/non-diagnostic OR.jpg]

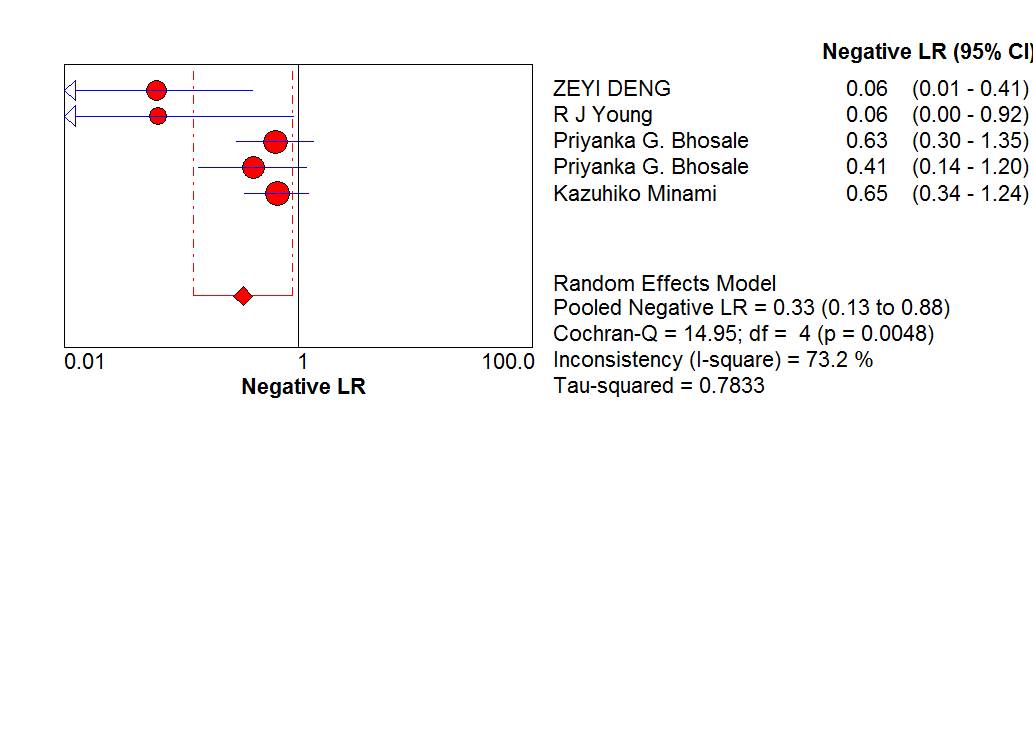

Supplement: Supplementary file 1 [file Data_Sheet_1.zip › Supplement file 2020.1.7/Supplementary file - figure/different countries/non-negative LR.jpg]

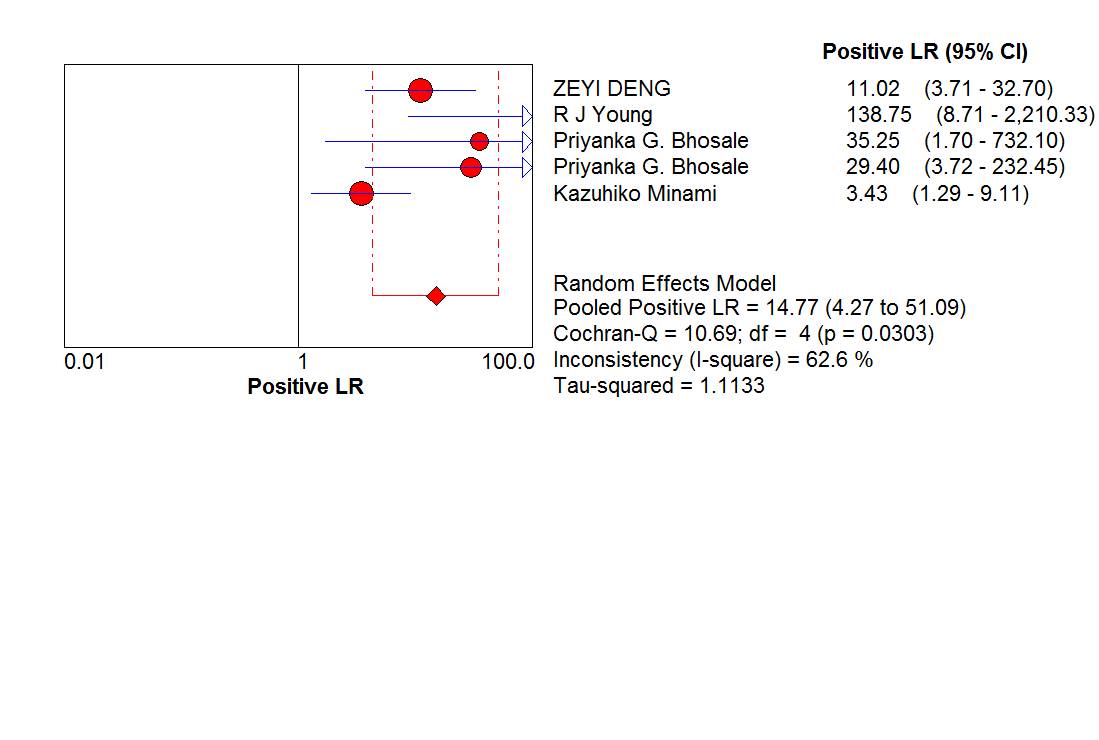

Supplement: Supplementary file 1 [file Data_Sheet_1.zip › Supplement file 2020.1.7/Supplementary file - figure/different countries/non-positive LR.jpg]

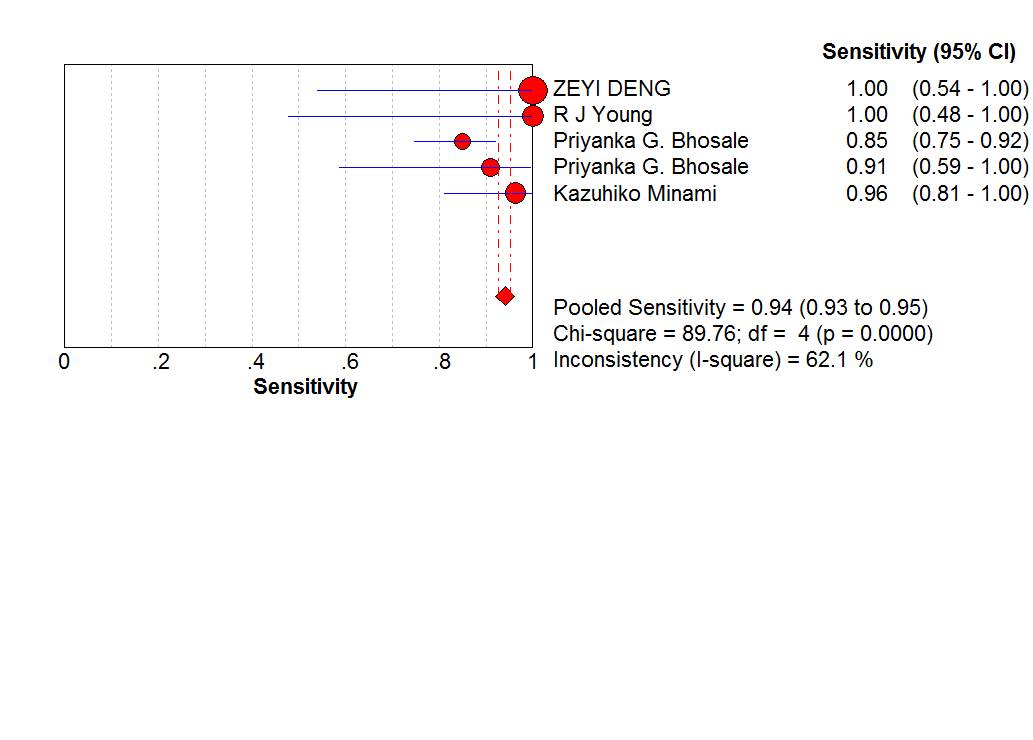

Supplement: Supplementary file 1 [file Data_Sheet_1.zip › Supplement file 2020.1.7/Supplementary file - figure/different countries/non-sensitivity.jpg]

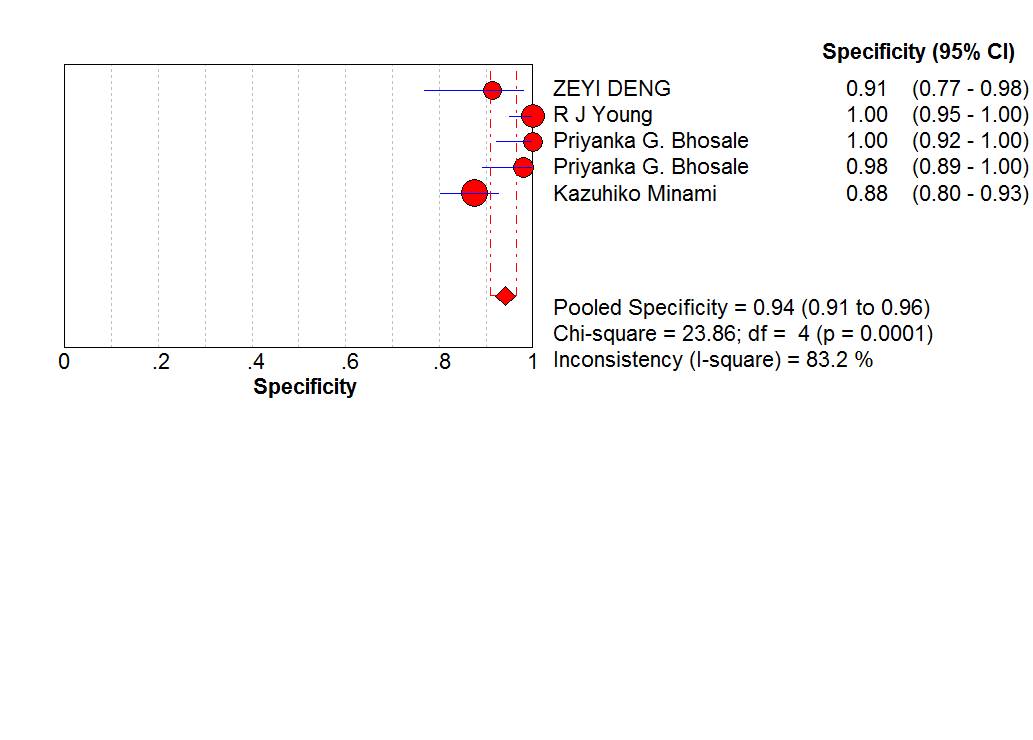

Supplement: Supplementary file 1 [file Data_Sheet_1.zip › Supplement file 2020.1.7/Supplementary file - figure/different countries/non-specificity.jpg]

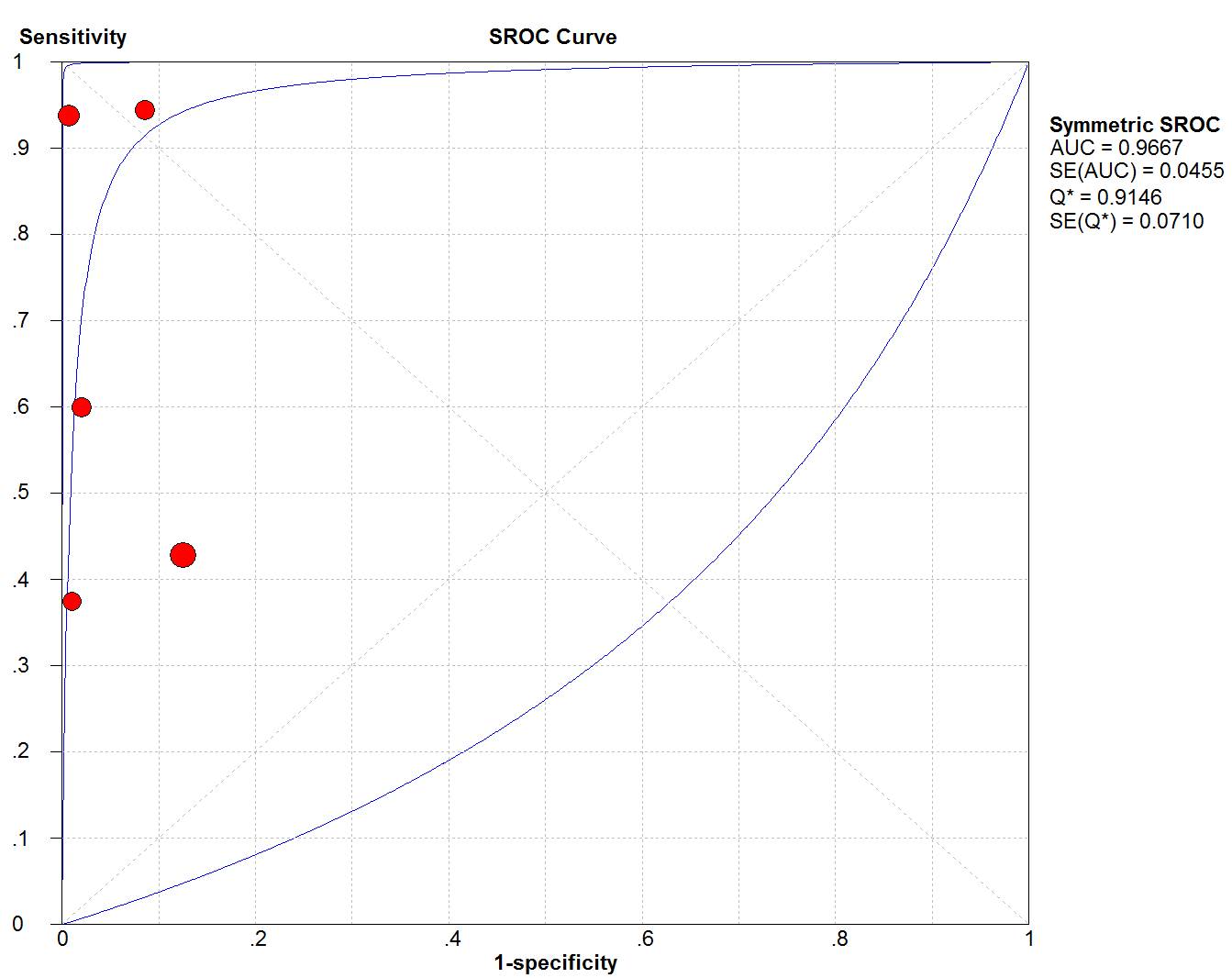

Supplement: Supplementary file 1 [file Data_Sheet_1.zip › Supplement file 2020.1.7/Supplementary file - figure/different countries/non-SROC curve.jpg]

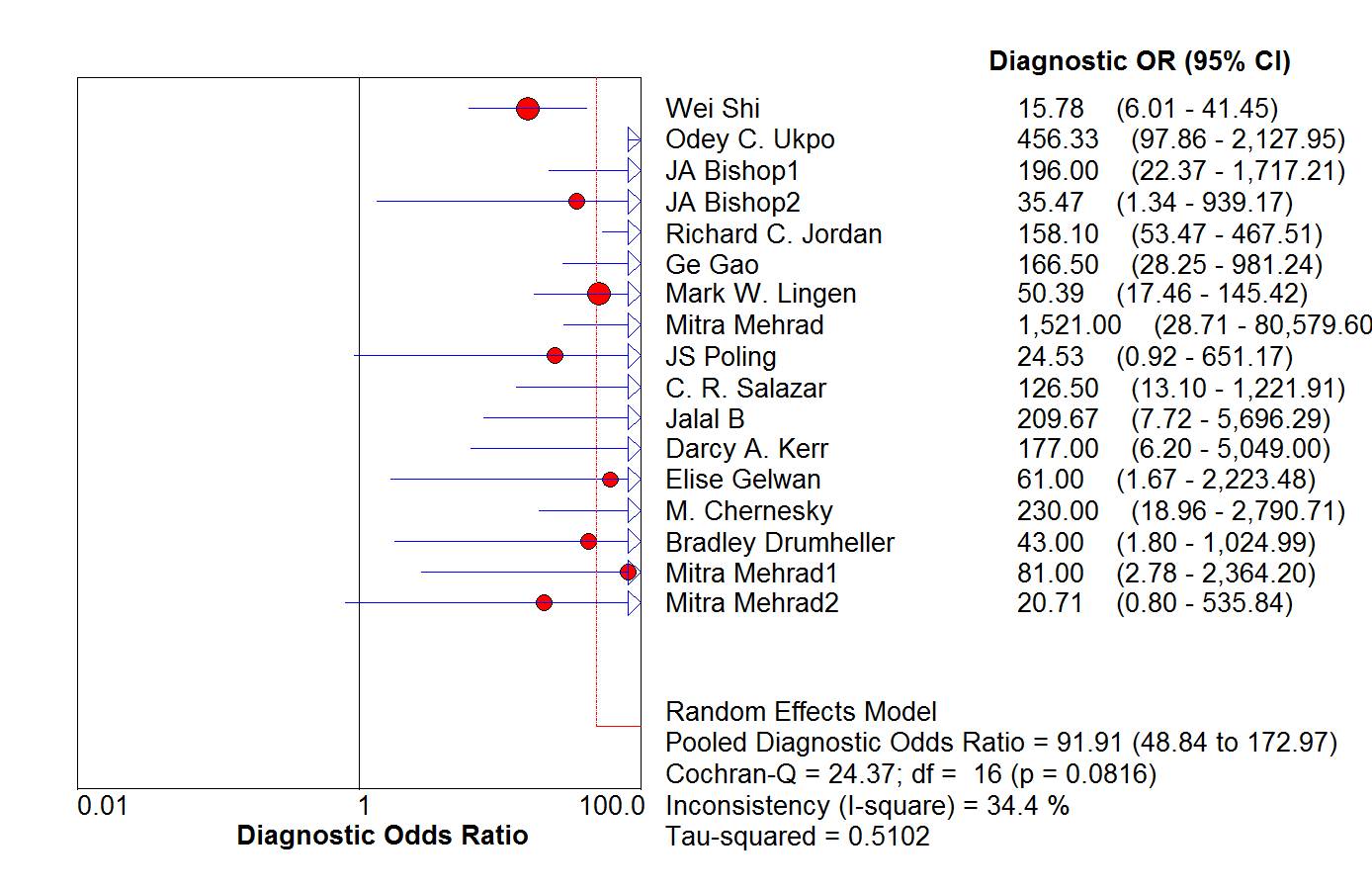

Supplement: Supplementary file 1 [file Data_Sheet_1.zip › Supplement file 2020.1.7/Supplementary file - figure/different countries/North America-diagnostic OR.jpg]

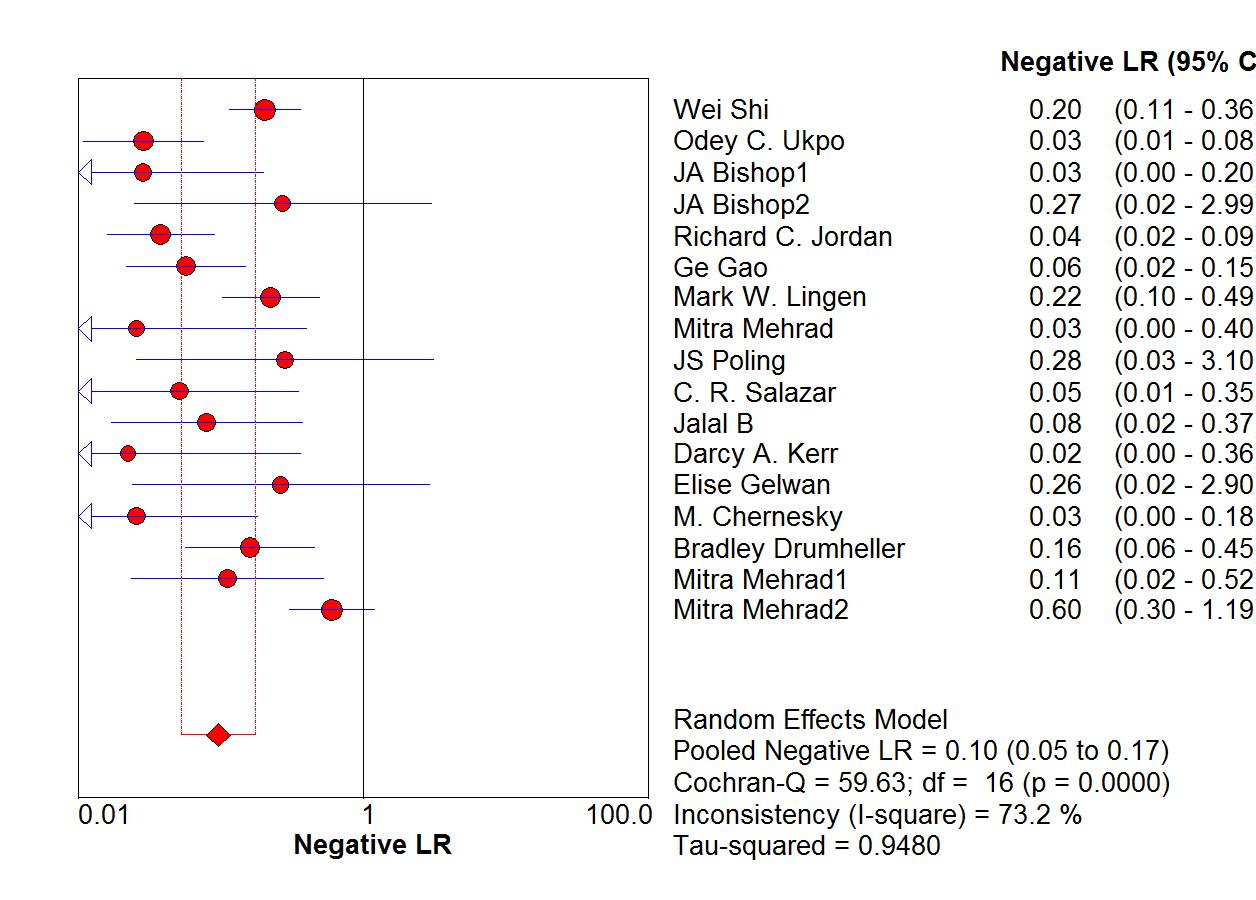

Supplement: Supplementary file 1 [file Data_Sheet_1.zip › Supplement file 2020.1.7/Supplementary file - figure/different countries/North America-negative LR.jpg]

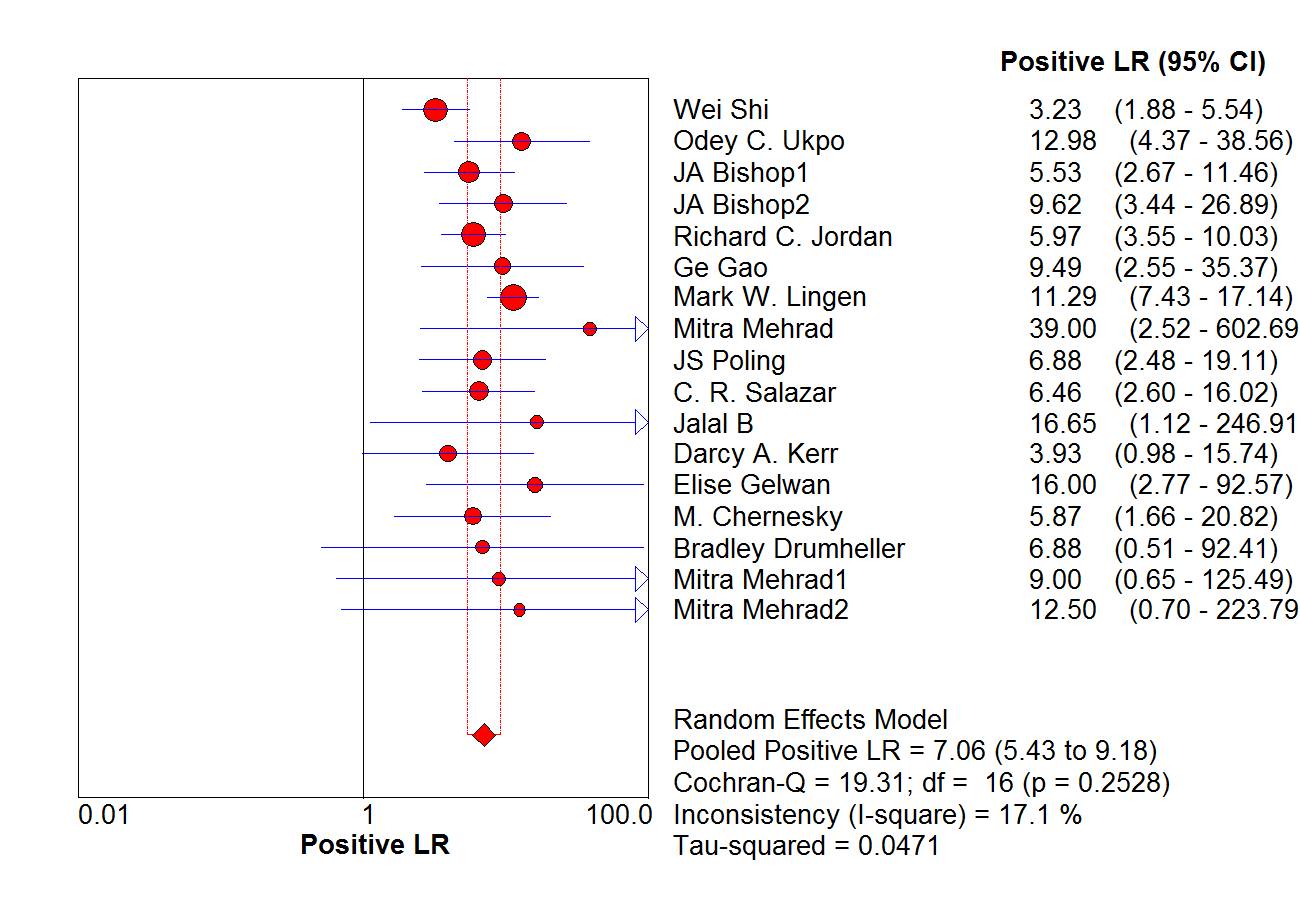

Supplement: Supplementary file 1 [file Data_Sheet_1.zip › Supplement file 2020.1.7/Supplementary file - figure/different countries/North America-positive LR.jpg]

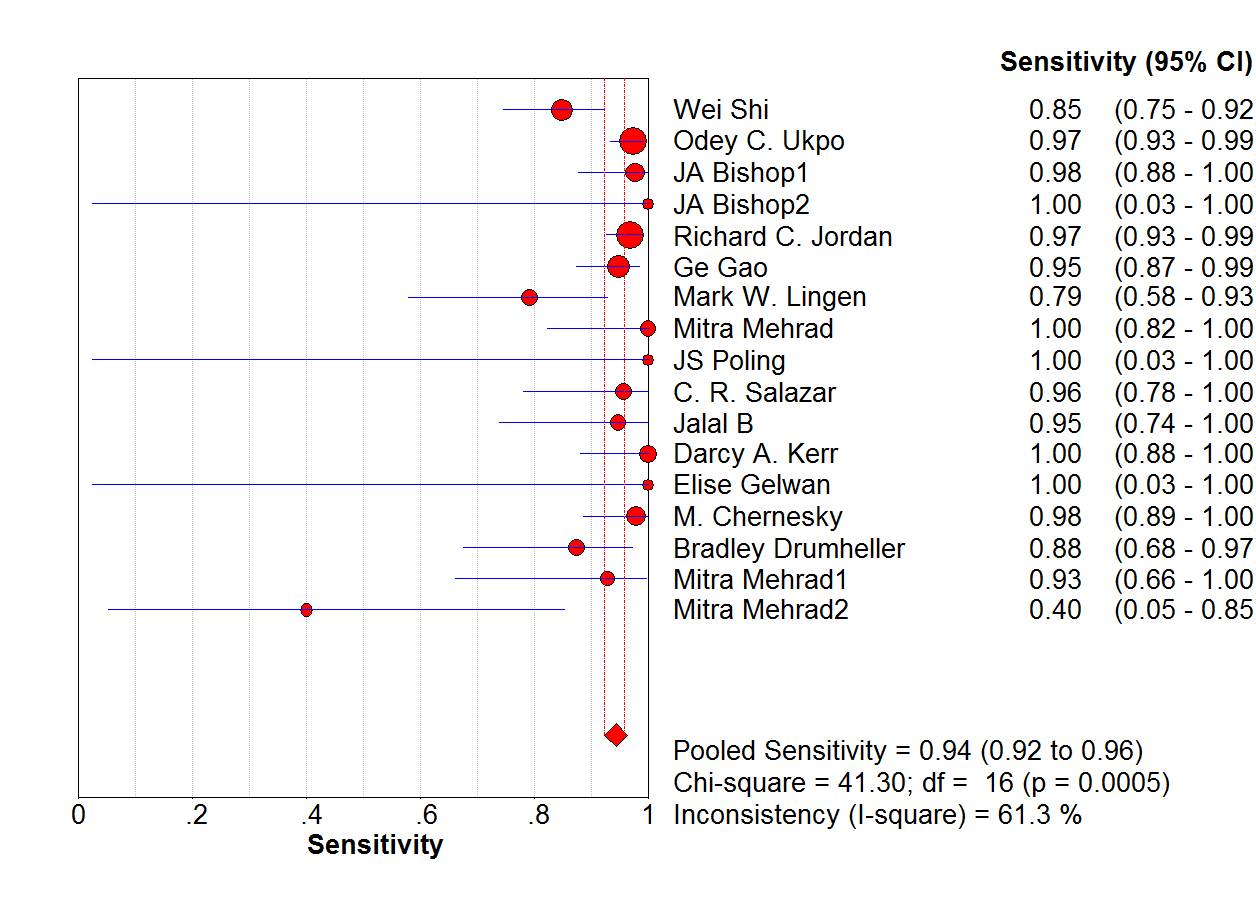

Supplement: Supplementary file 1 [file Data_Sheet_1.zip › Supplement file 2020.1.7/Supplementary file - figure/different countries/North America-sensitivity.jpg]

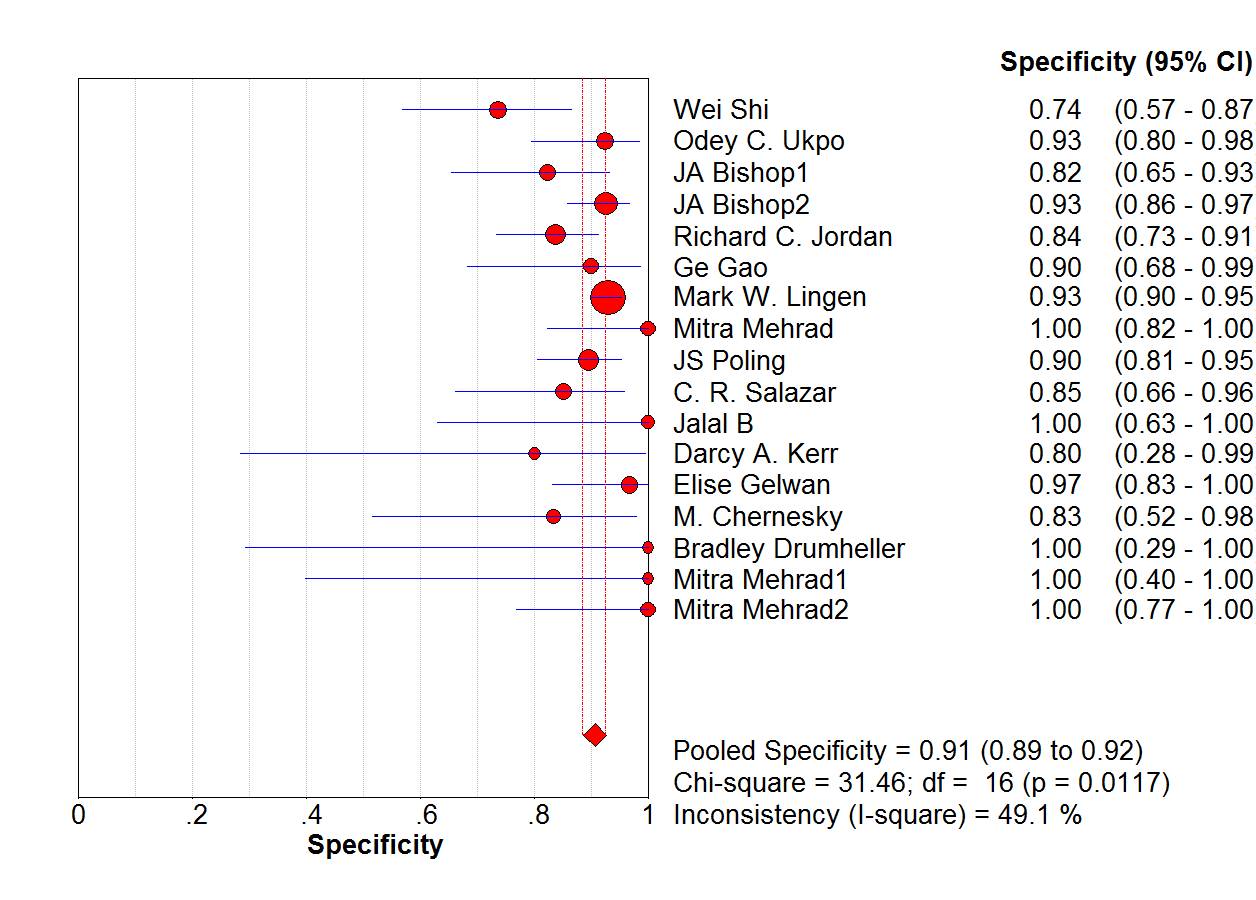

Supplement: Supplementary file 1 [file Data_Sheet_1.zip › Supplement file 2020.1.7/Supplementary file - figure/different countries/North America-specificity.jpg]

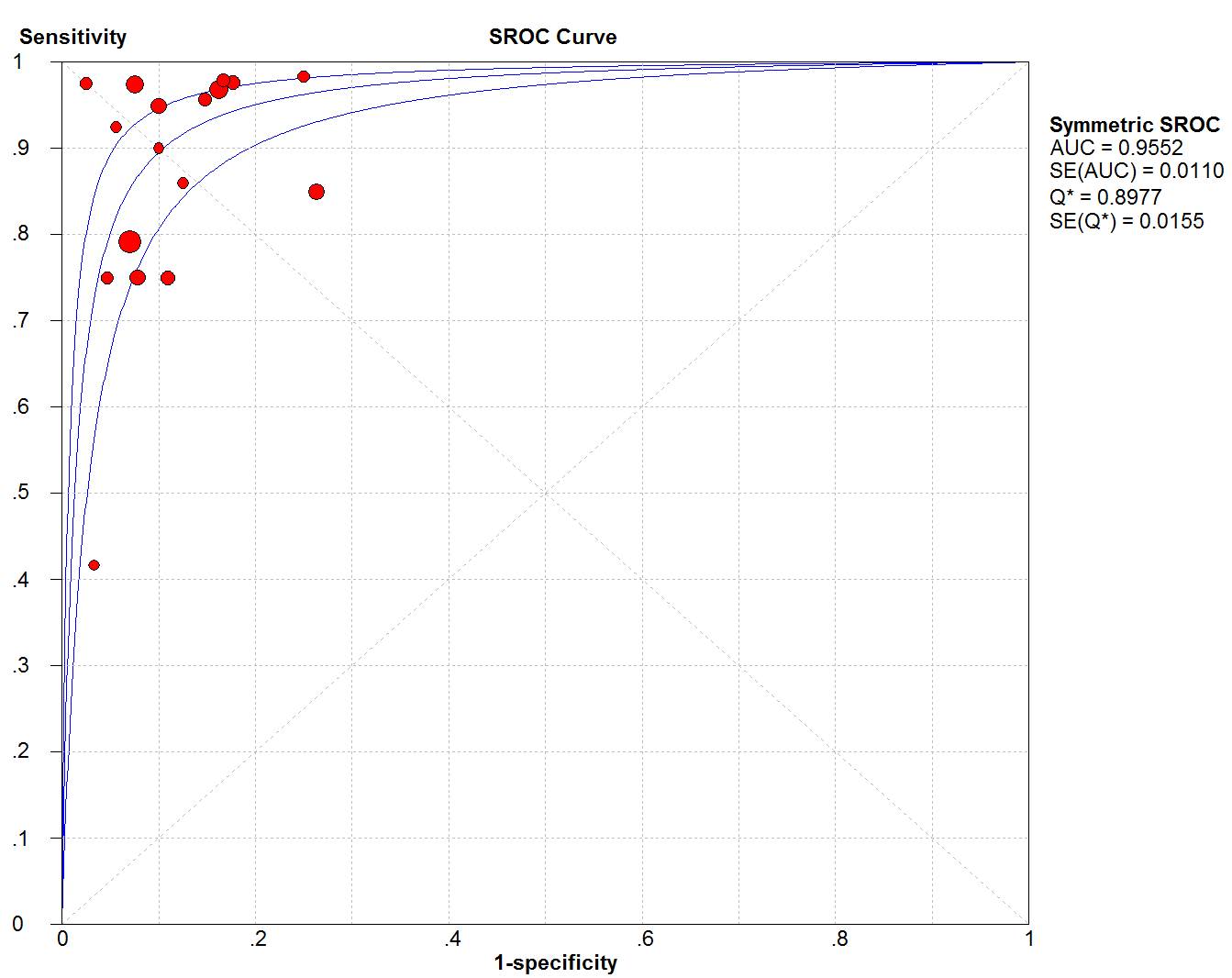

Supplement: Supplementary file 1 [file Data_Sheet_1.zip › Supplement file 2020.1.7/Supplementary file - figure/different countries/North America-SROC curve.jpg]

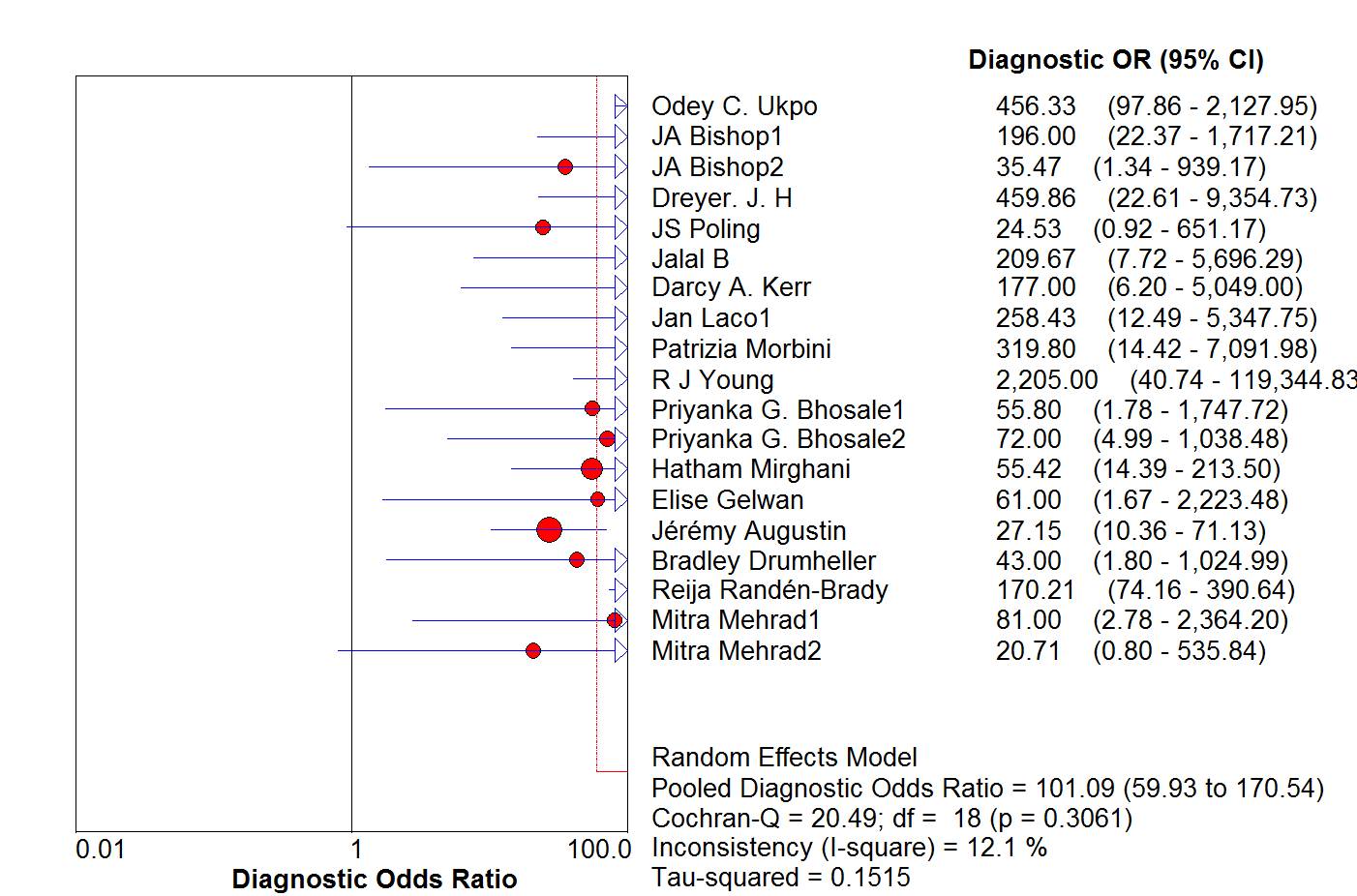

Supplement: Supplementary file 1 [file Data_Sheet_1.zip › Supplement file 2020.1.7/Supplementary file - figure/ISH or PCR/ISH diagnostic OR.jpg]

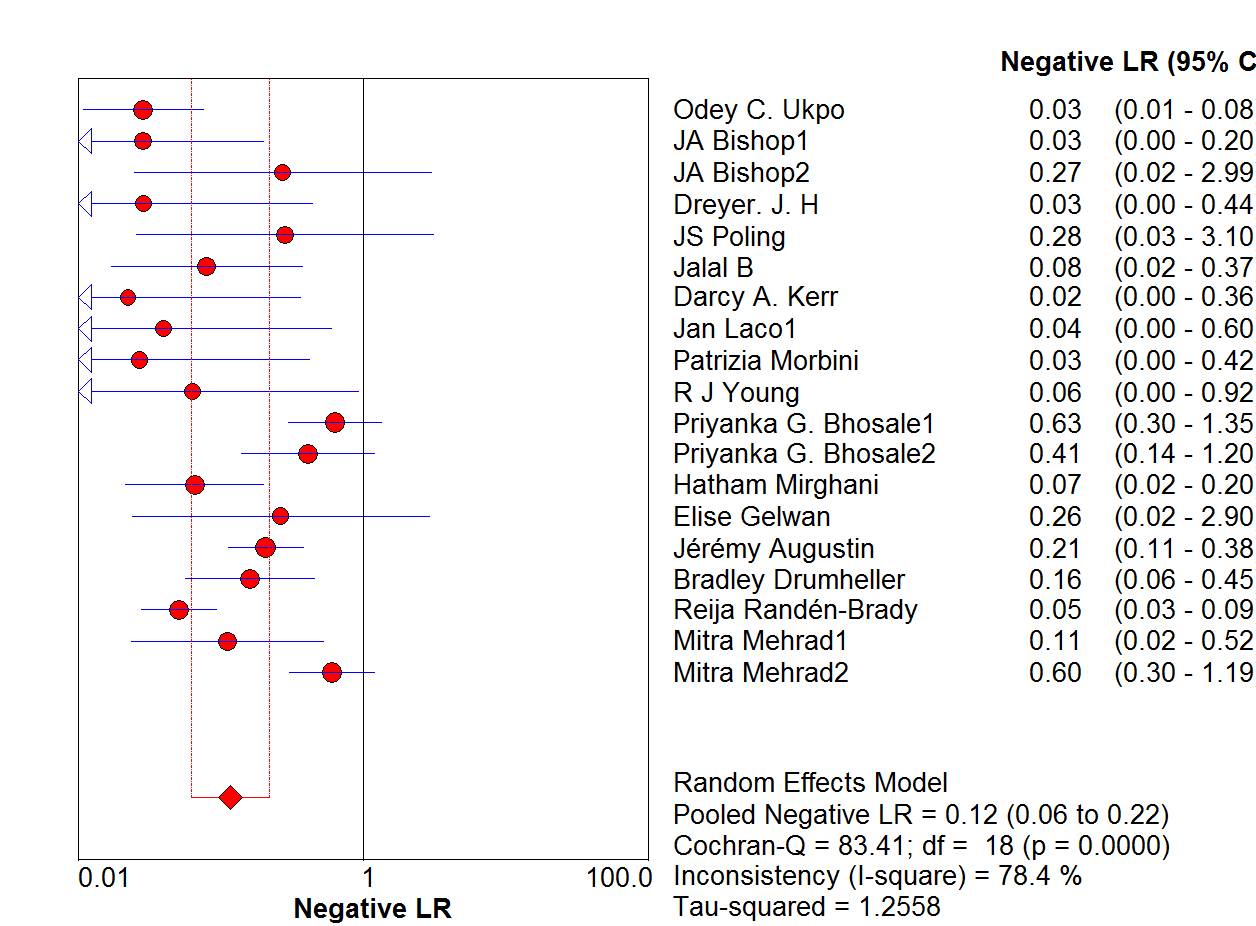

Supplement: Supplementary file 1 [file Data_Sheet_1.zip › Supplement file 2020.1.7/Supplementary file - figure/ISH or PCR/ISH negative LR.jpg]

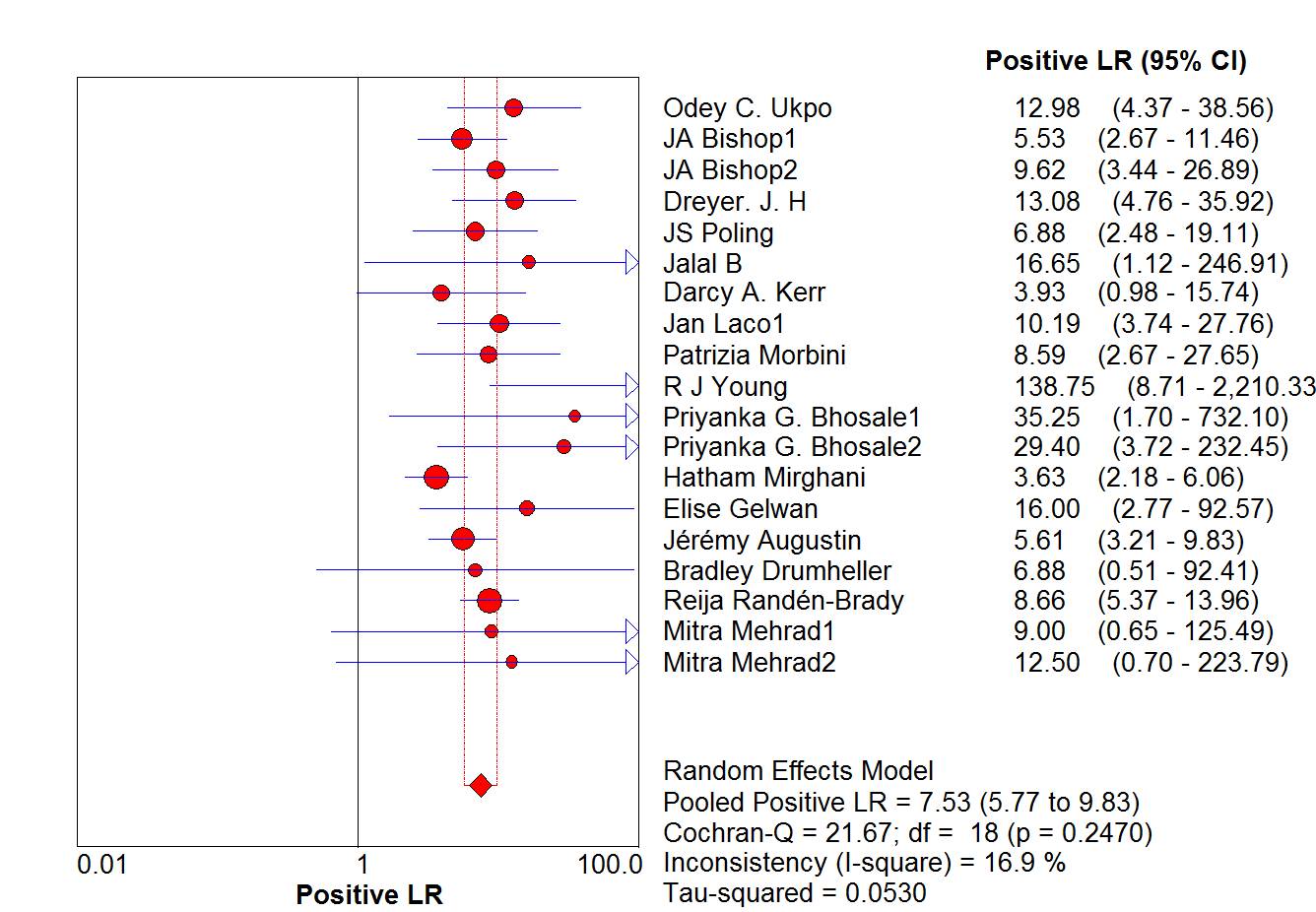

Supplement: Supplementary file 1 [file Data_Sheet_1.zip › Supplement file 2020.1.7/Supplementary file - figure/ISH or PCR/ISH positive LR.jpg]

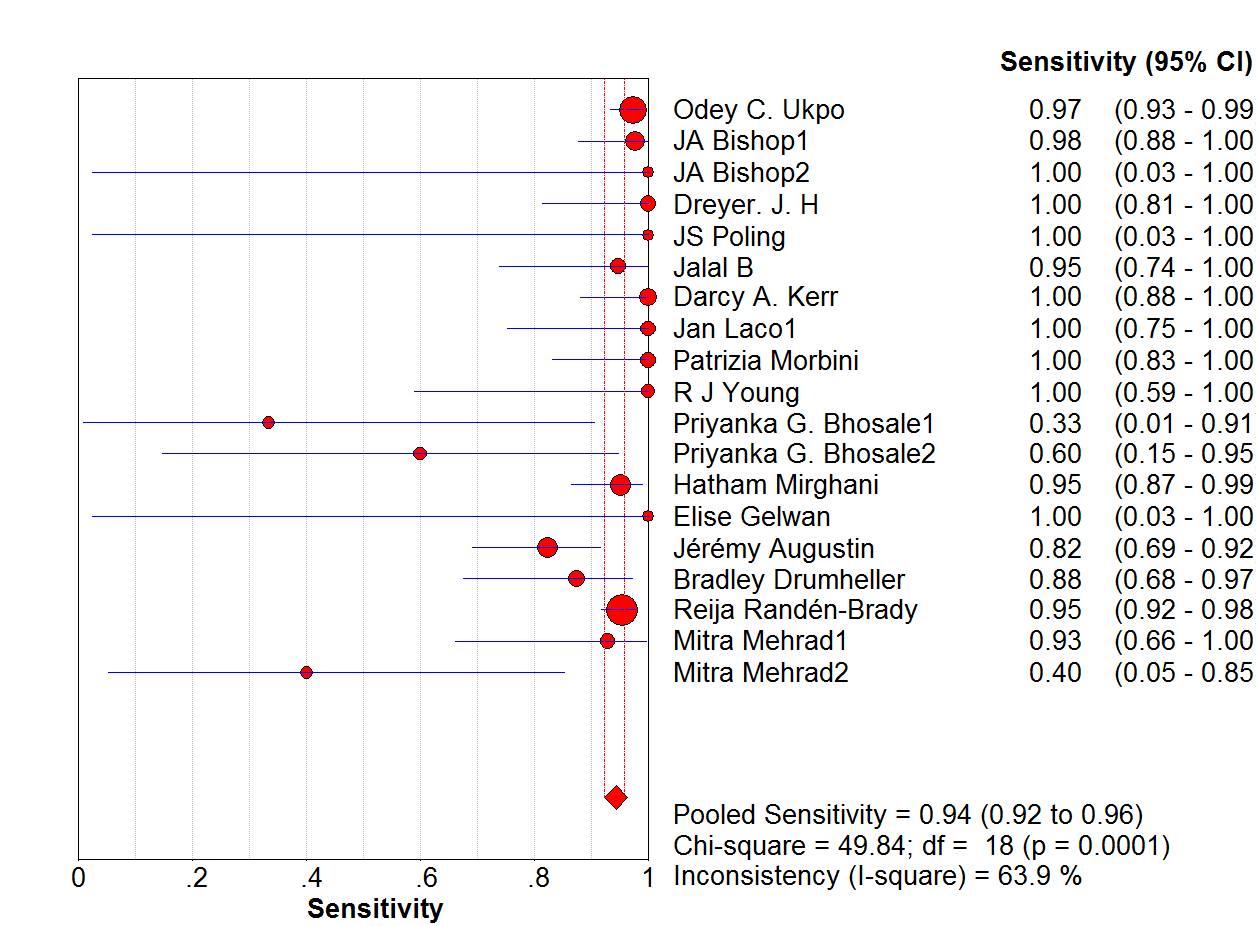

Supplement: Supplementary file 1 [file Data_Sheet_1.zip › Supplement file 2020.1.7/Supplementary file - figure/ISH or PCR/ISH sensitivity.jpg]

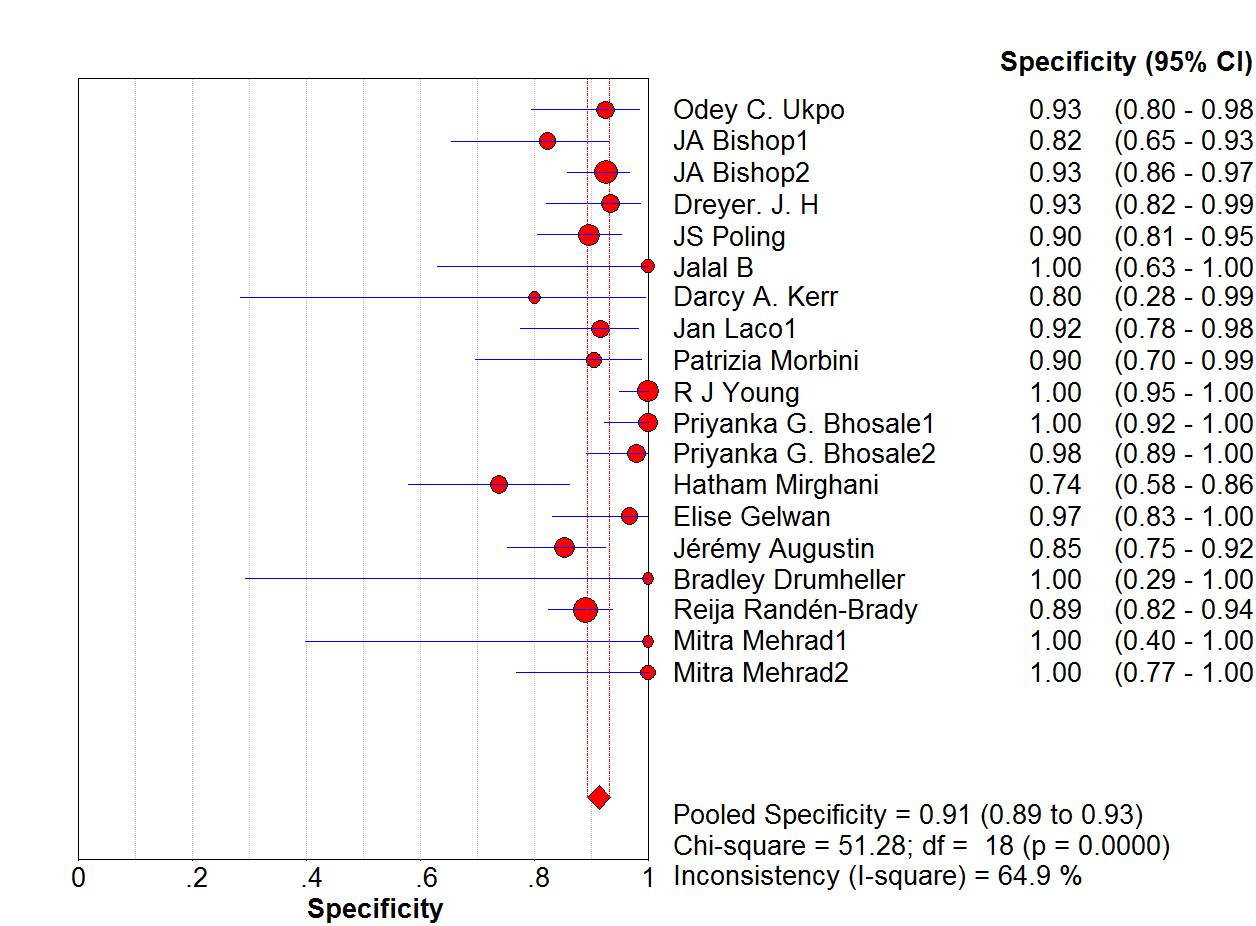

Supplement: Supplementary file 1 [file Data_Sheet_1.zip › Supplement file 2020.1.7/Supplementary file - figure/ISH or PCR/ISH specificity.jpg]

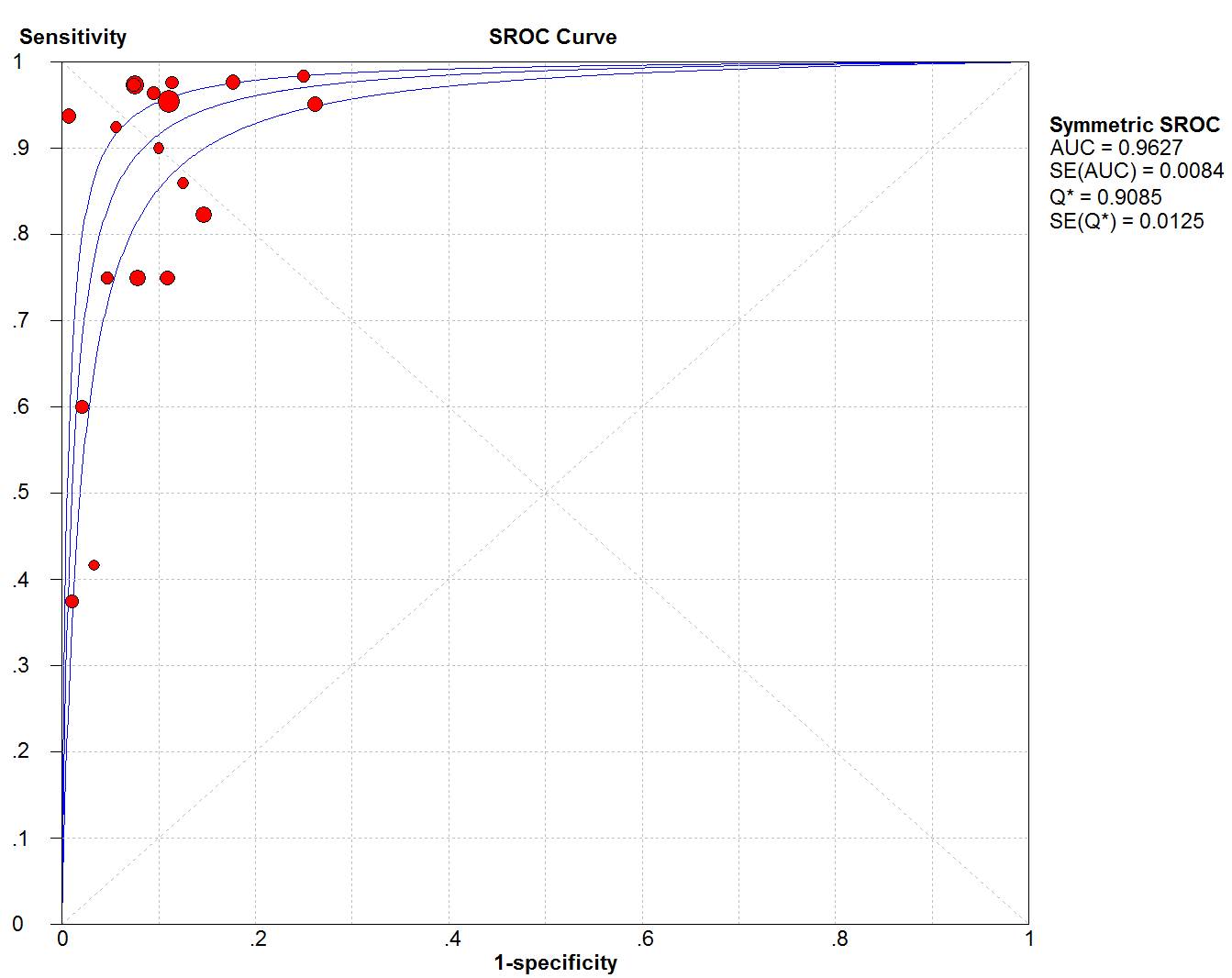

Supplement: Supplementary file 1 [file Data_Sheet_1.zip › Supplement file 2020.1.7/Supplementary file - figure/ISH or PCR/ISH SROC curve.jpg]

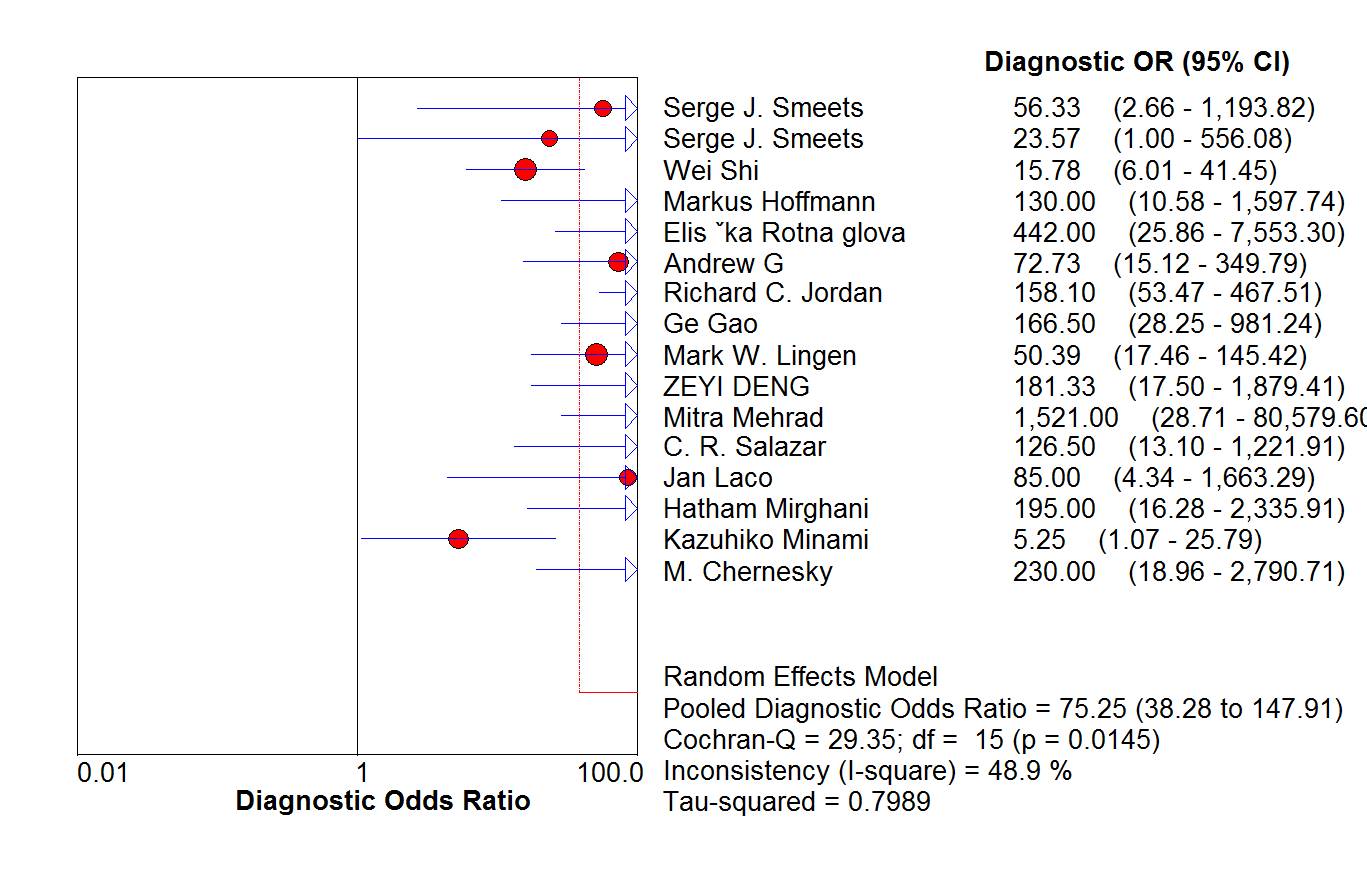

Supplement: Supplementary file 1 [file Data_Sheet_1.zip › Supplement file 2020.1.7/Supplementary file - figure/ISH or PCR/PCR diagnostic OR.jpg]

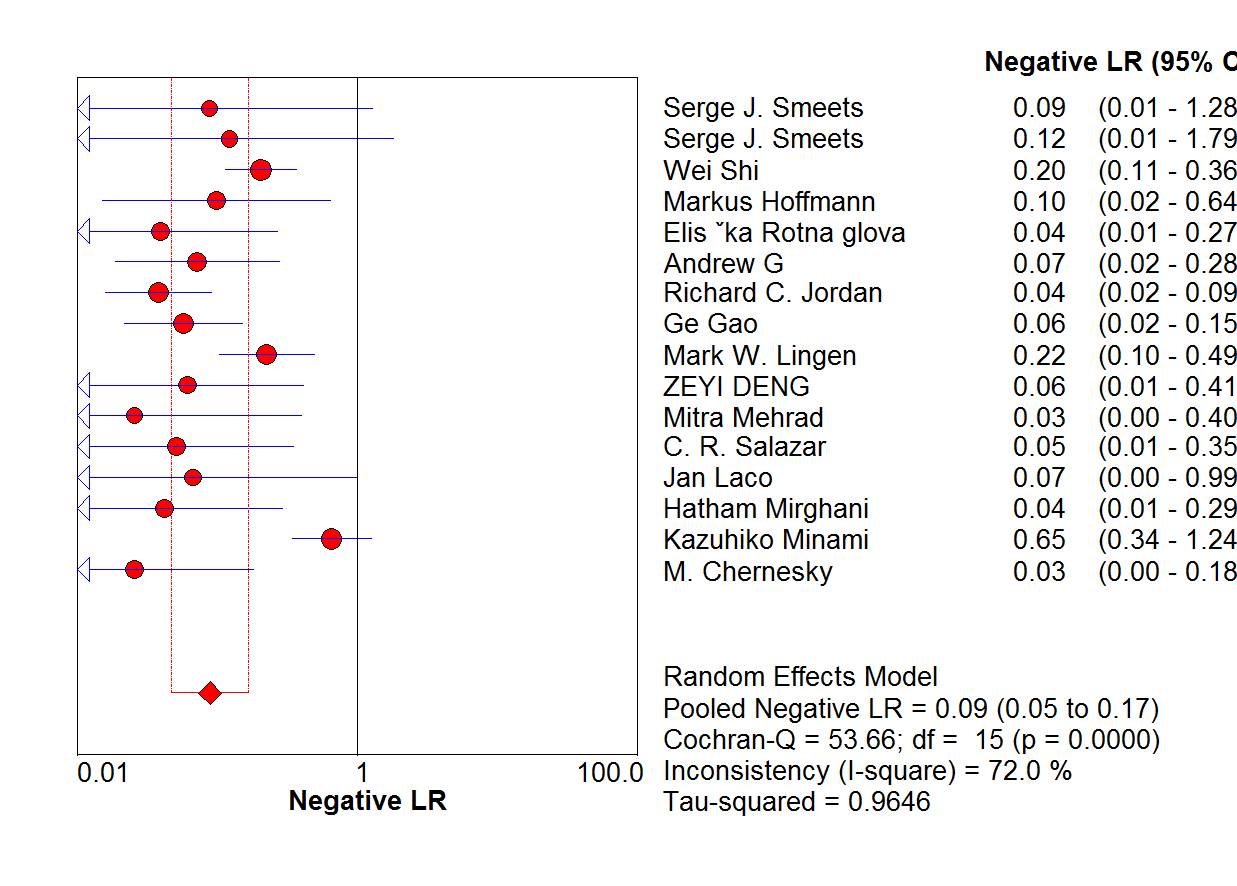

Supplement: Supplementary file 1 [file Data_Sheet_1.zip › Supplement file 2020.1.7/Supplementary file - figure/ISH or PCR/PCR negative LR.jpg]

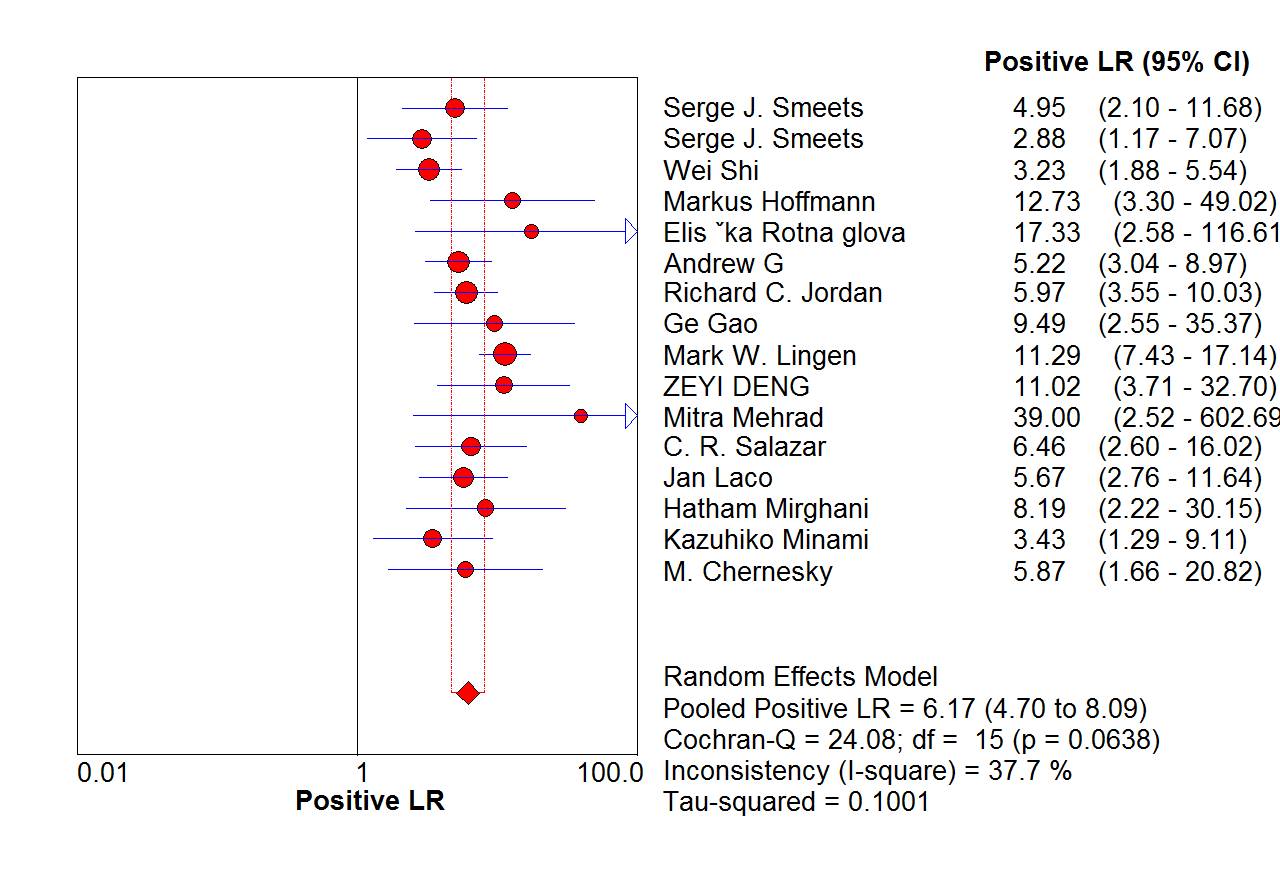

Supplement: Supplementary file 1 [file Data_Sheet_1.zip › Supplement file 2020.1.7/Supplementary file - figure/ISH or PCR/PCR positive LR.jpg]

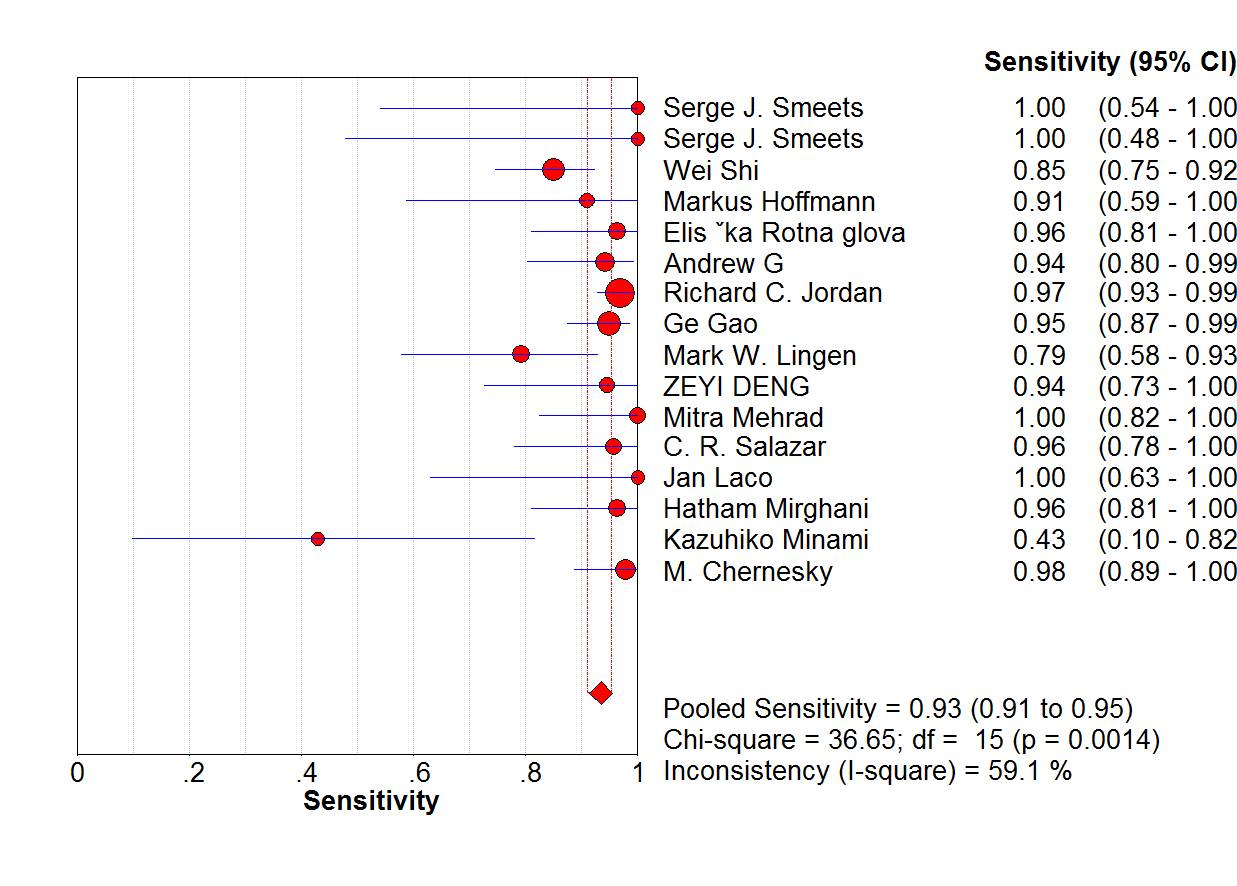

Supplement: Supplementary file 1 [file Data_Sheet_1.zip › Supplement file 2020.1.7/Supplementary file - figure/ISH or PCR/PCR sensitivity.jpg]

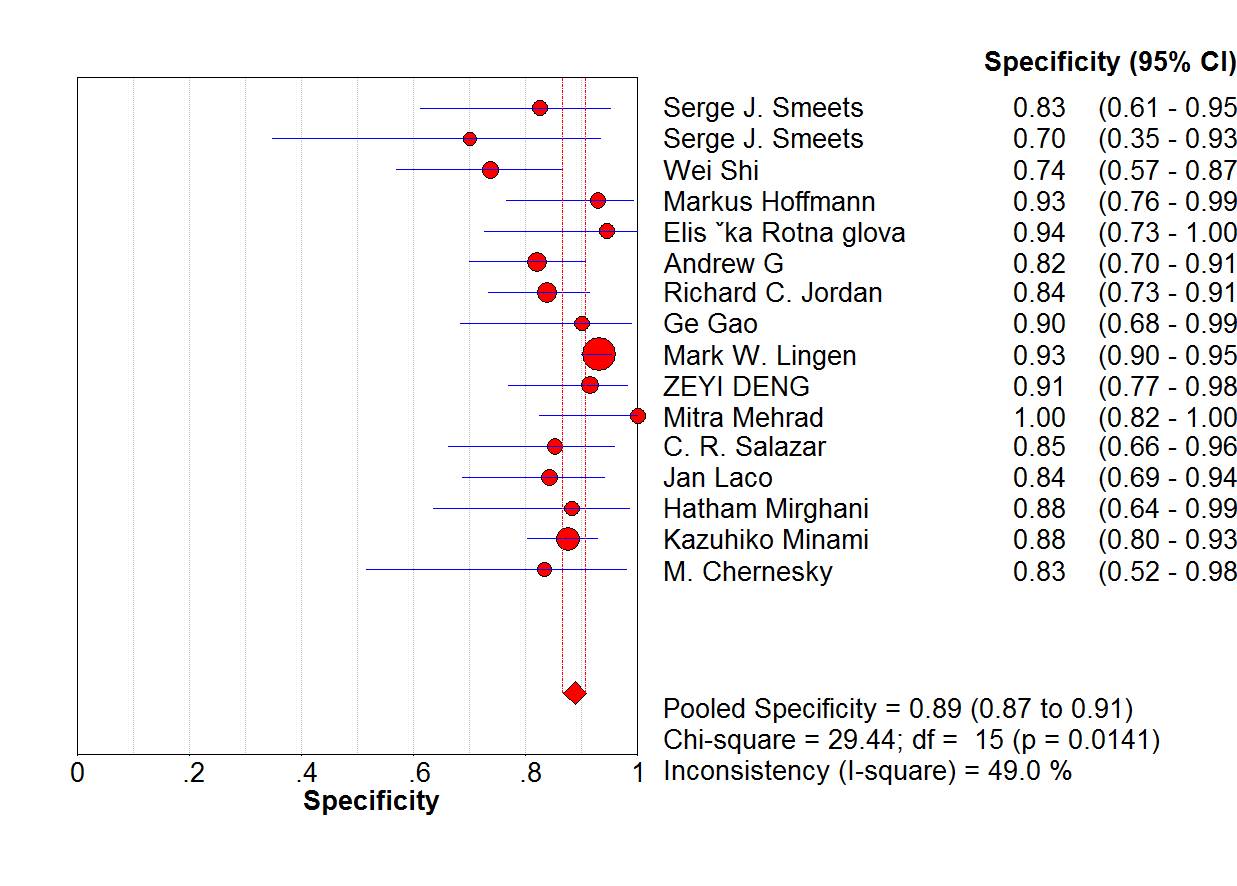

Supplement: Supplementary file 1 [file Data_Sheet_1.zip › Supplement file 2020.1.7/Supplementary file - figure/ISH or PCR/PCR specificity.jpg]

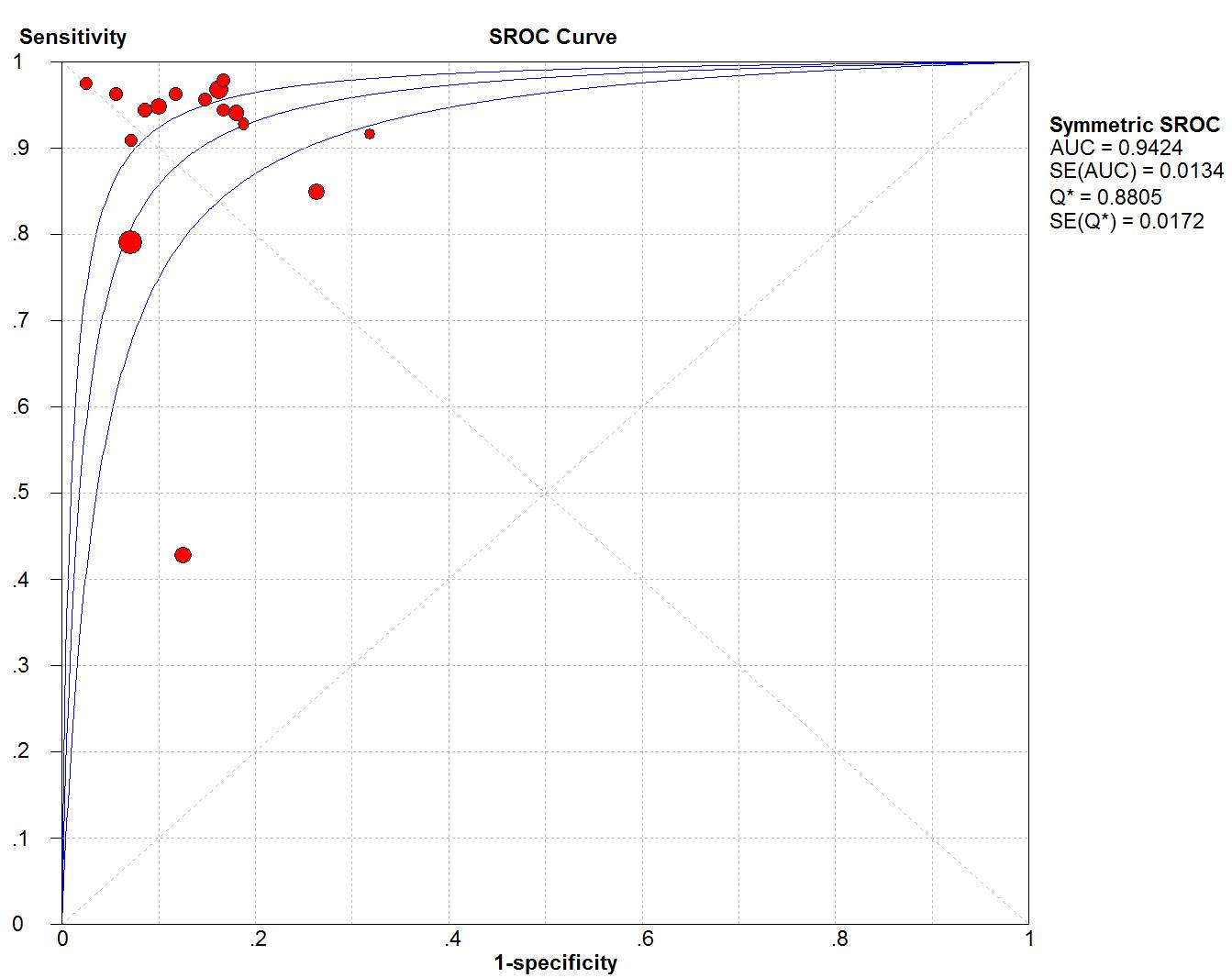

Supplement: Supplementary file 1 [file Data_Sheet_1.zip › Supplement file 2020.1.7/Supplementary file - figure/ISH or PCR/PCR SROC curve.jpg]

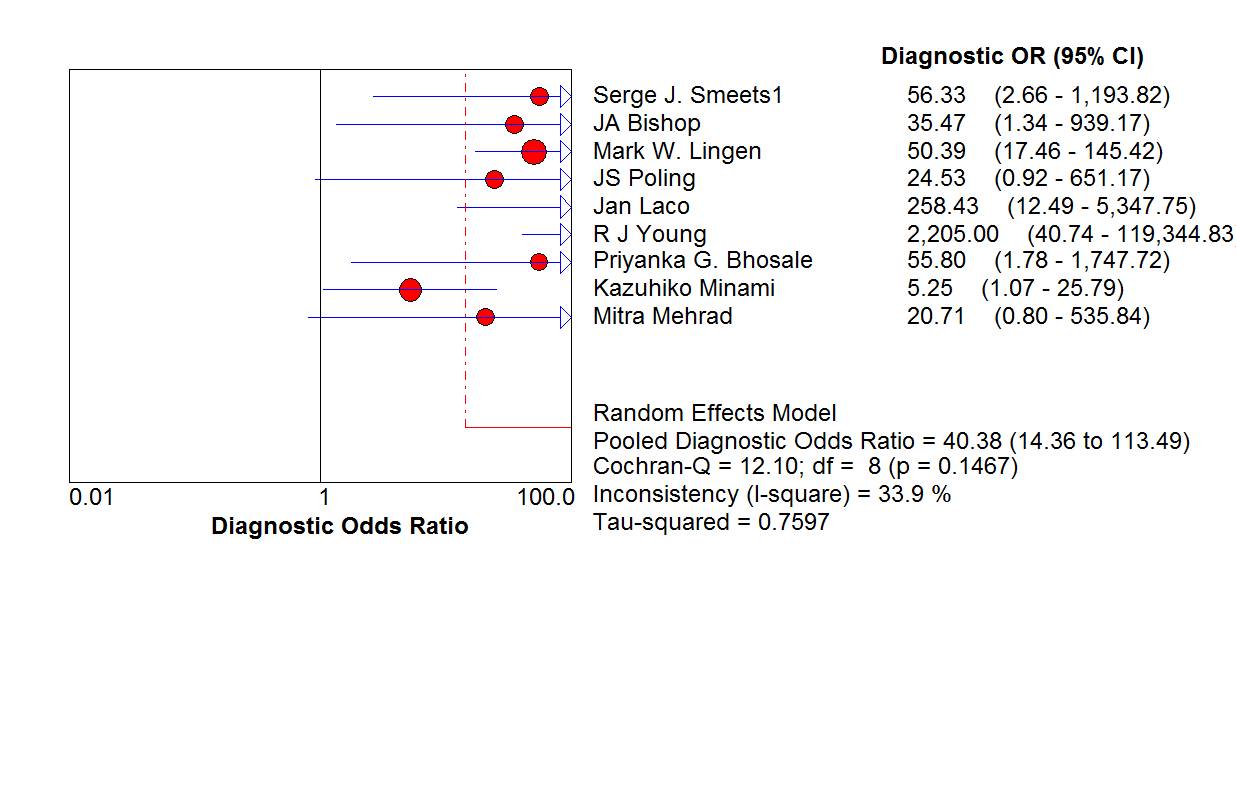

Supplement: Supplementary file 1 [file Data_Sheet_1.zip › Supplement file 2020.1.7/Supplementary file - figure/OP or no-OP/noOP-diagnostic OR.jpg]

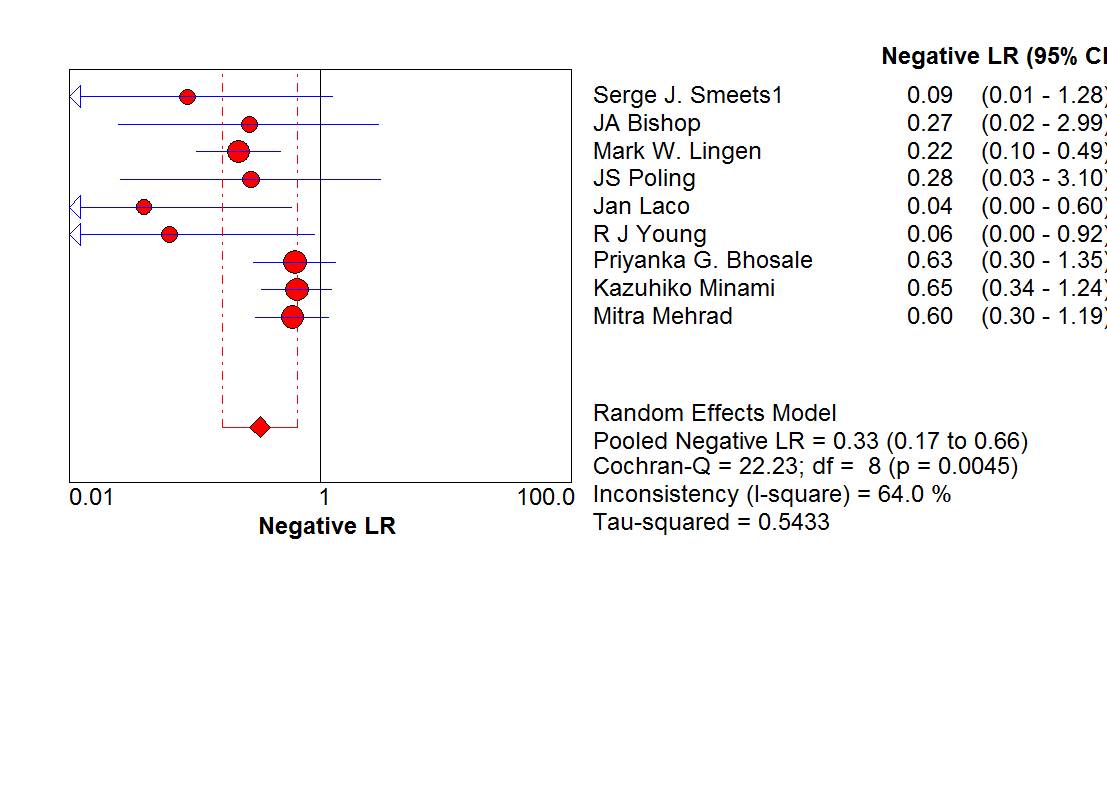

Supplement: Supplementary file 1 [file Data_Sheet_1.zip › Supplement file 2020.1.7/Supplementary file - figure/OP or no-OP/noOP-negativre LR.jpg]

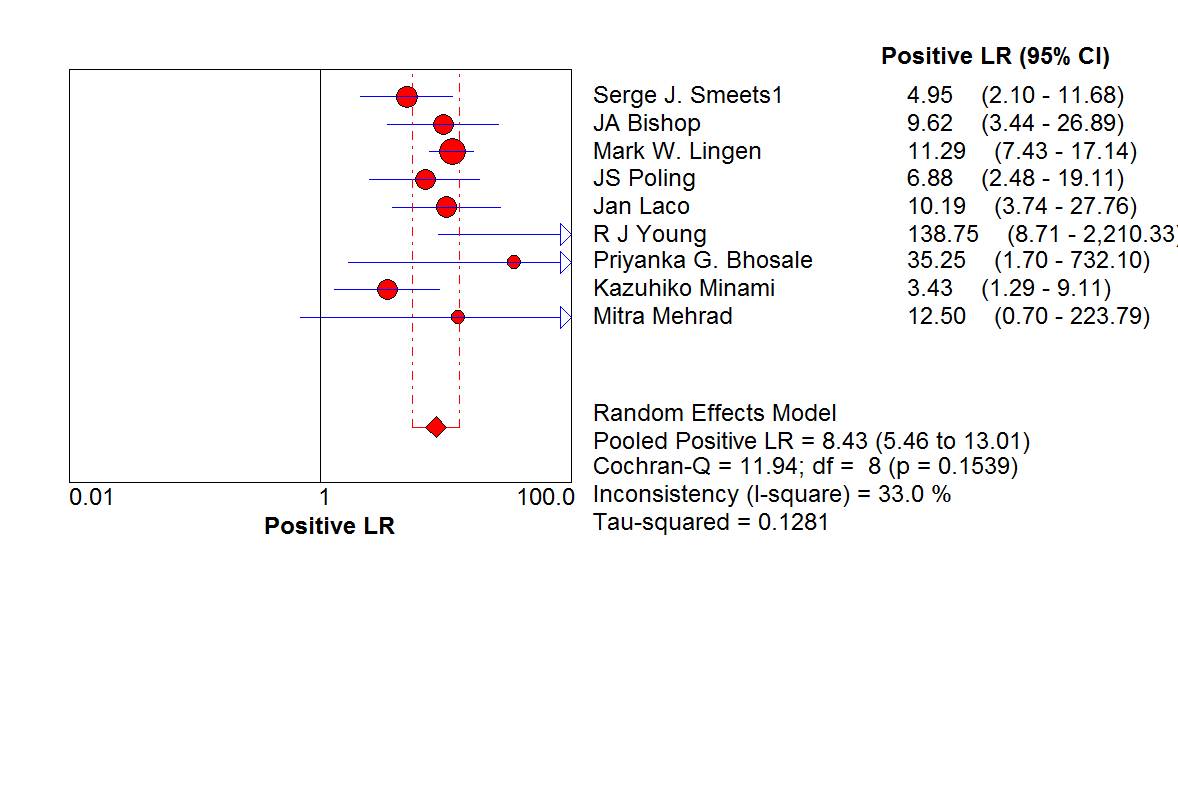

Supplement: Supplementary file 1 [file Data_Sheet_1.zip › Supplement file 2020.1.7/Supplementary file - figure/OP or no-OP/noOP-positive LR.jpg]

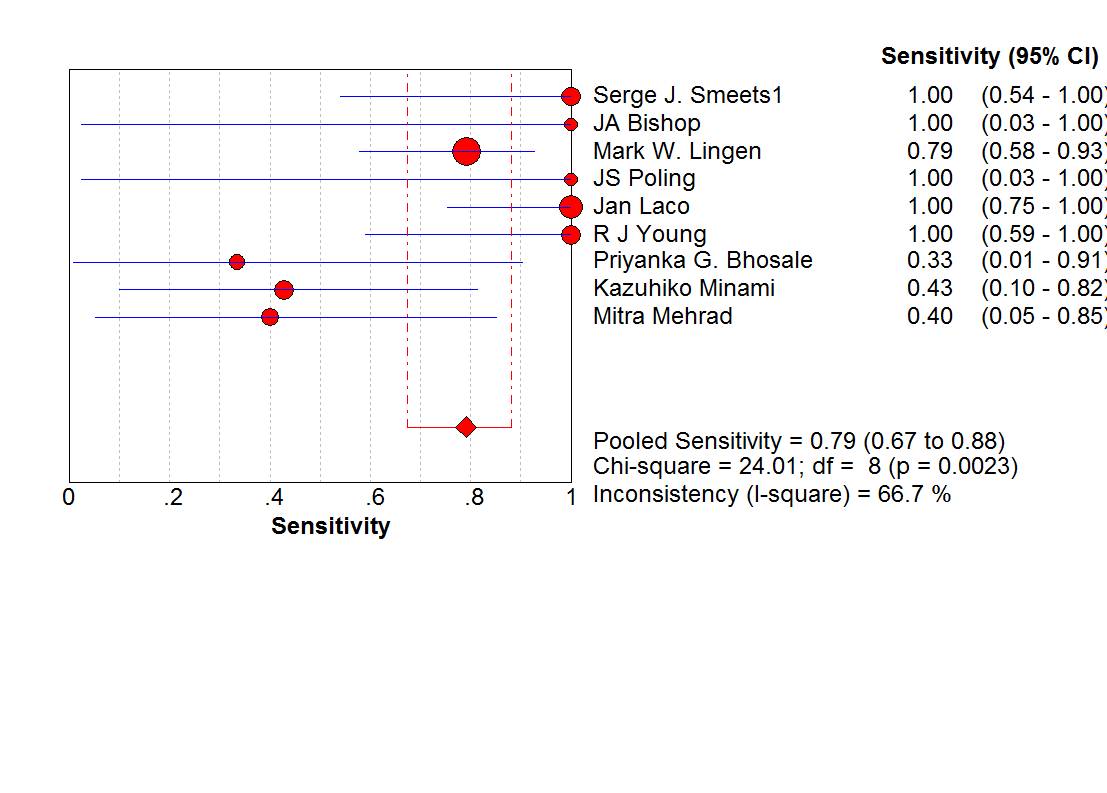

Supplement: Supplementary file 1 [file Data_Sheet_1.zip › Supplement file 2020.1.7/Supplementary file - figure/OP or no-OP/noOP-sensitivity.jpg]

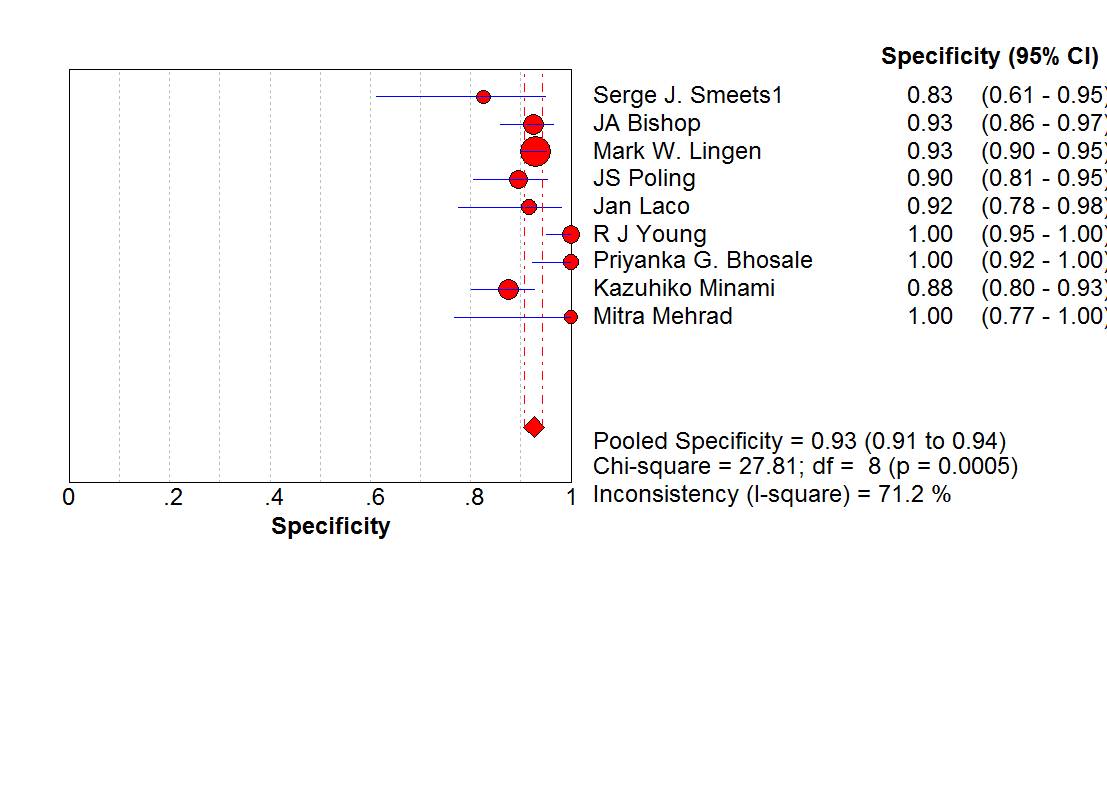

Supplement: Supplementary file 1 [file Data_Sheet_1.zip › Supplement file 2020.1.7/Supplementary file - figure/OP or no-OP/noOP-specificity.jpg]

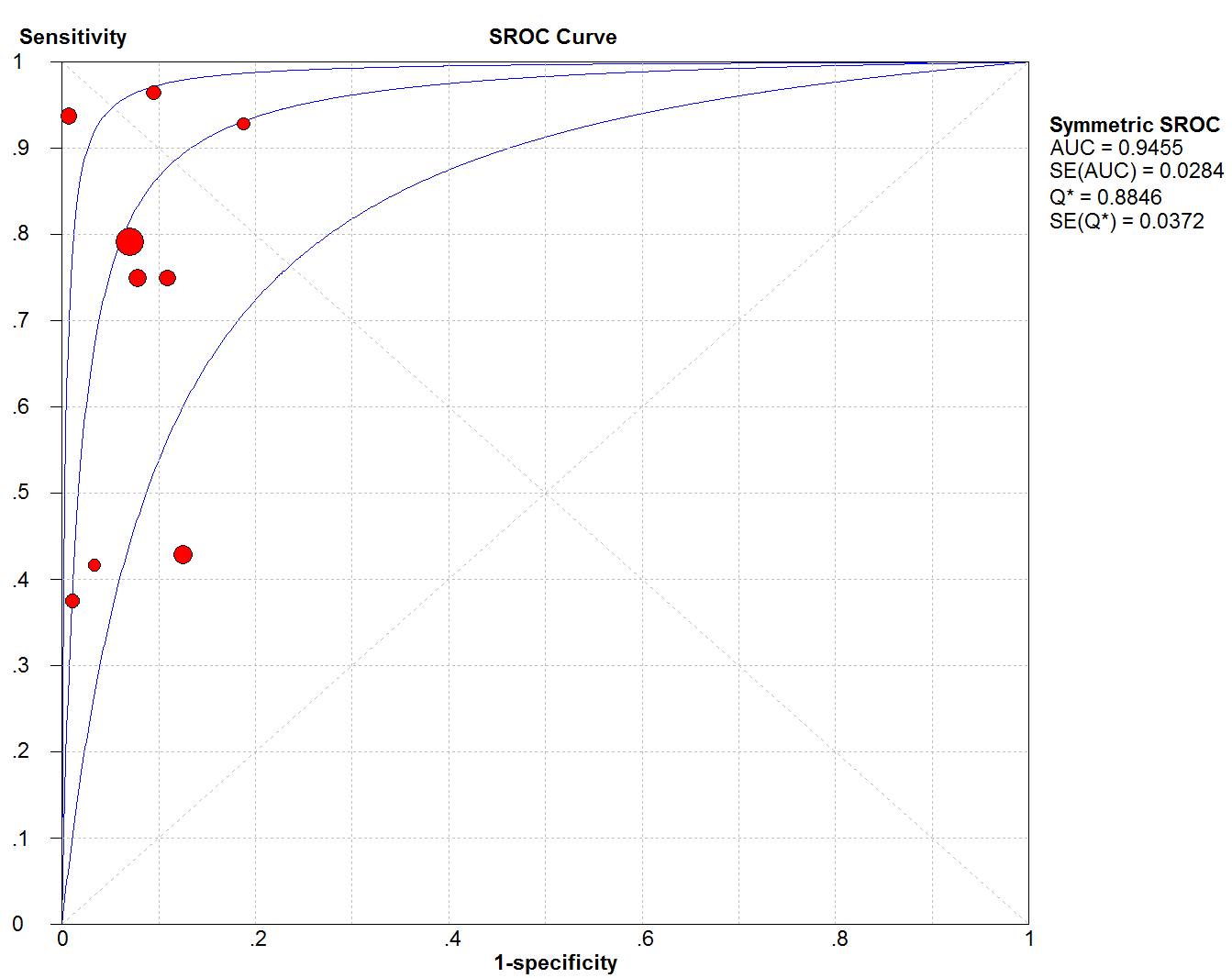

Supplement: Supplementary file 1 [file Data_Sheet_1.zip › Supplement file 2020.1.7/Supplementary file - figure/OP or no-OP/noOP-SROC curve.jpg]

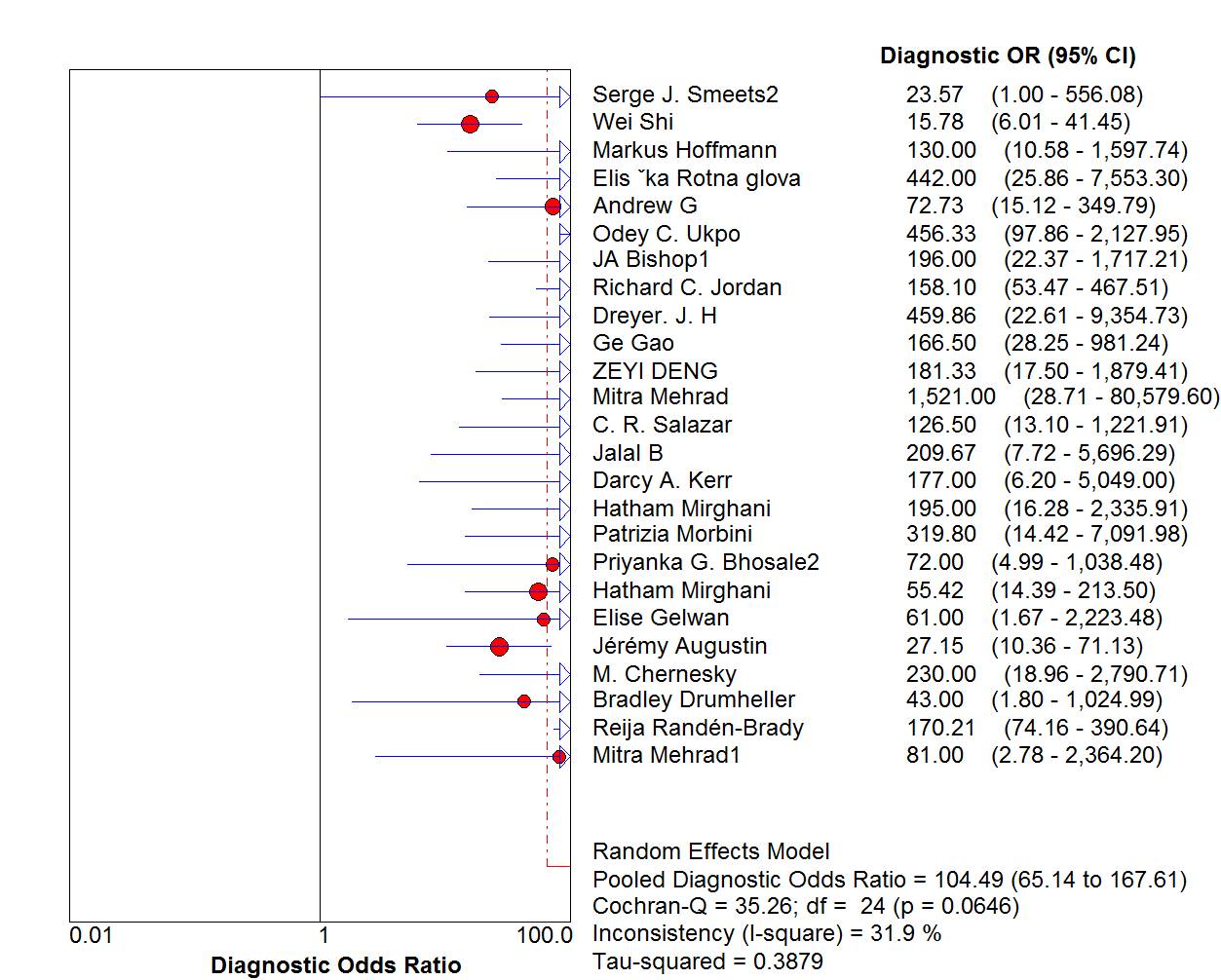

Supplement: Supplementary file 1 [file Data_Sheet_1.zip › Supplement file 2020.1.7/Supplementary file - figure/OP or no-OP/OP-diagnostic OR.jpg]

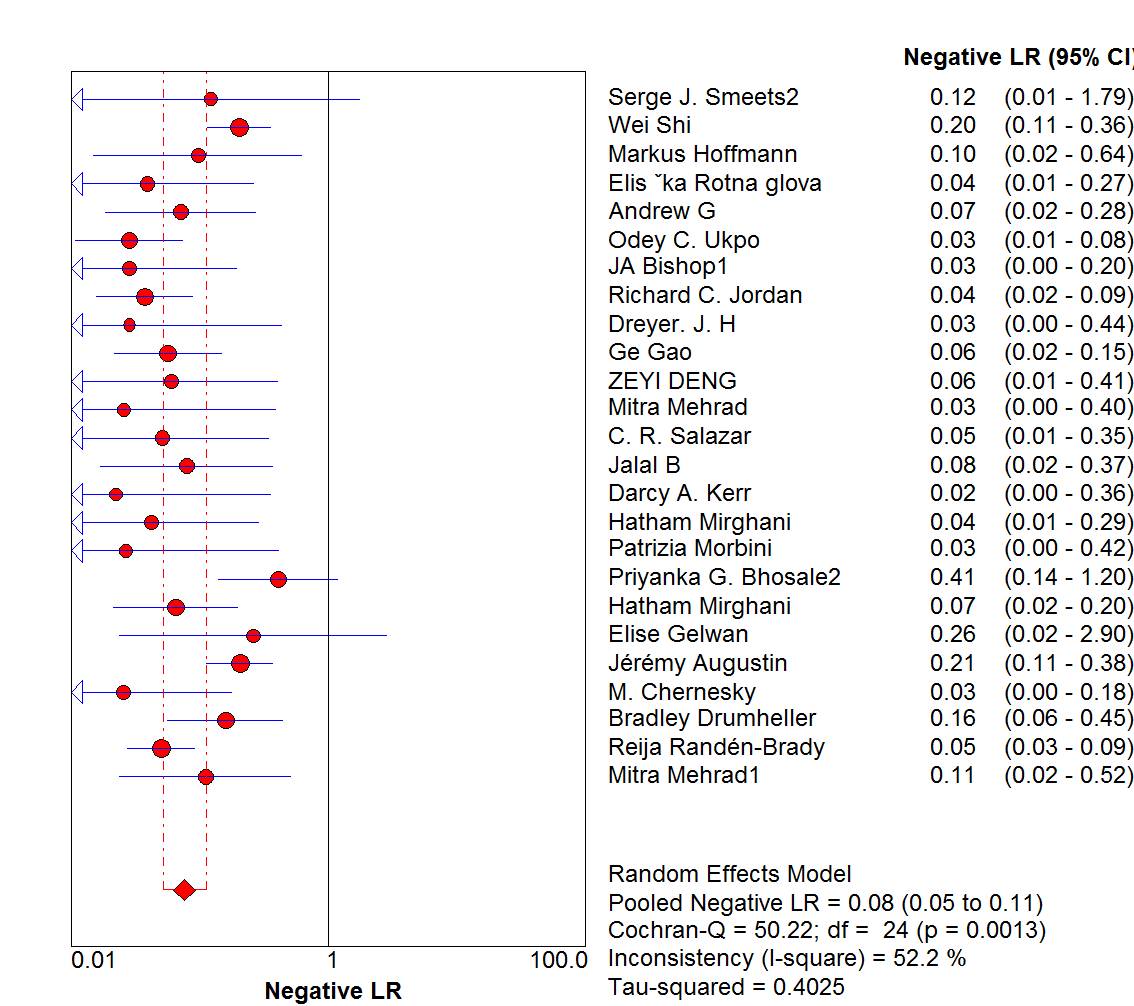

Supplement: Supplementary file 1 [file Data_Sheet_1.zip › Supplement file 2020.1.7/Supplementary file - figure/OP or no-OP/OP-negative LR.jpg]

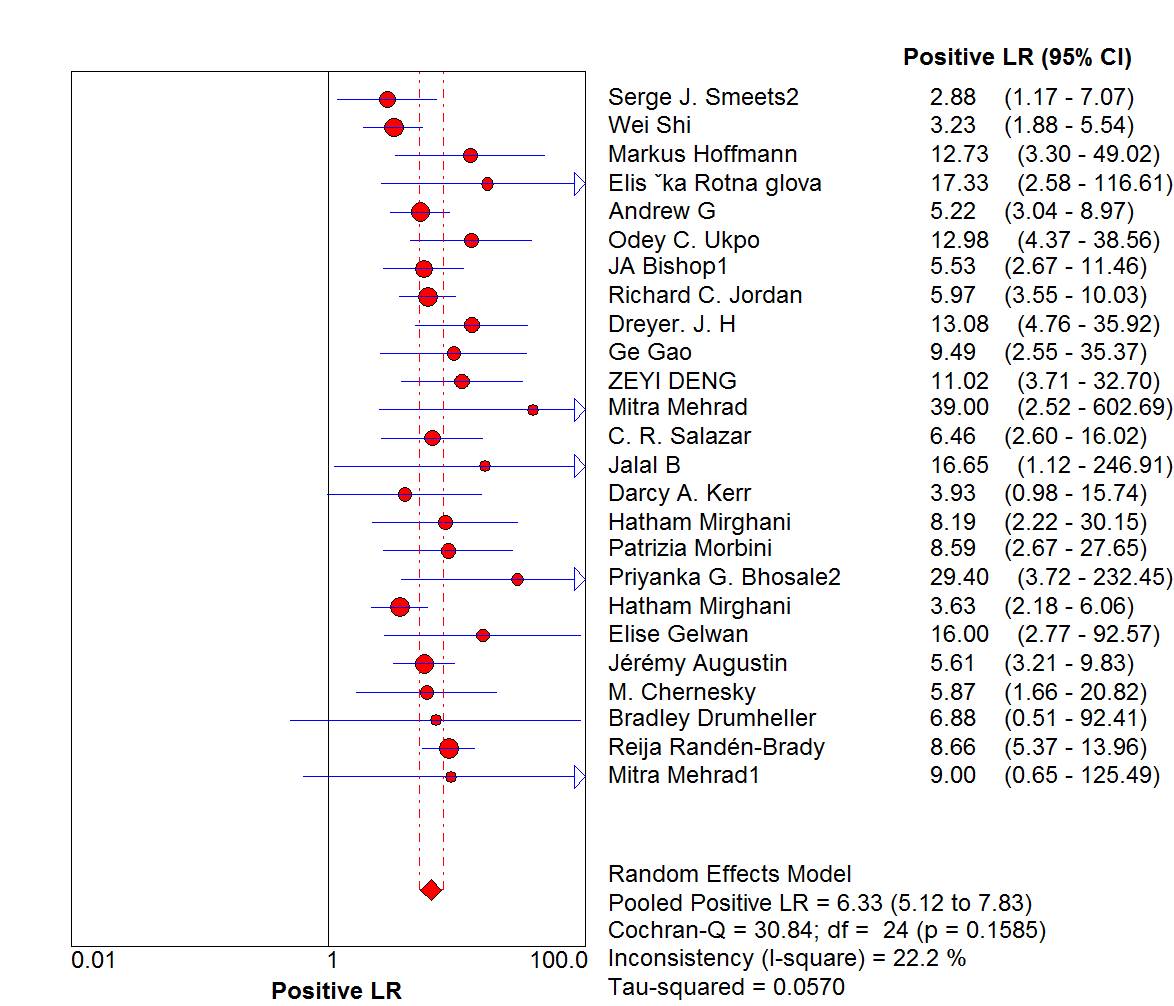

Supplement: Supplementary file 1 [file Data_Sheet_1.zip › Supplement file 2020.1.7/Supplementary file - figure/OP or no-OP/OP-positive LR.jpg]

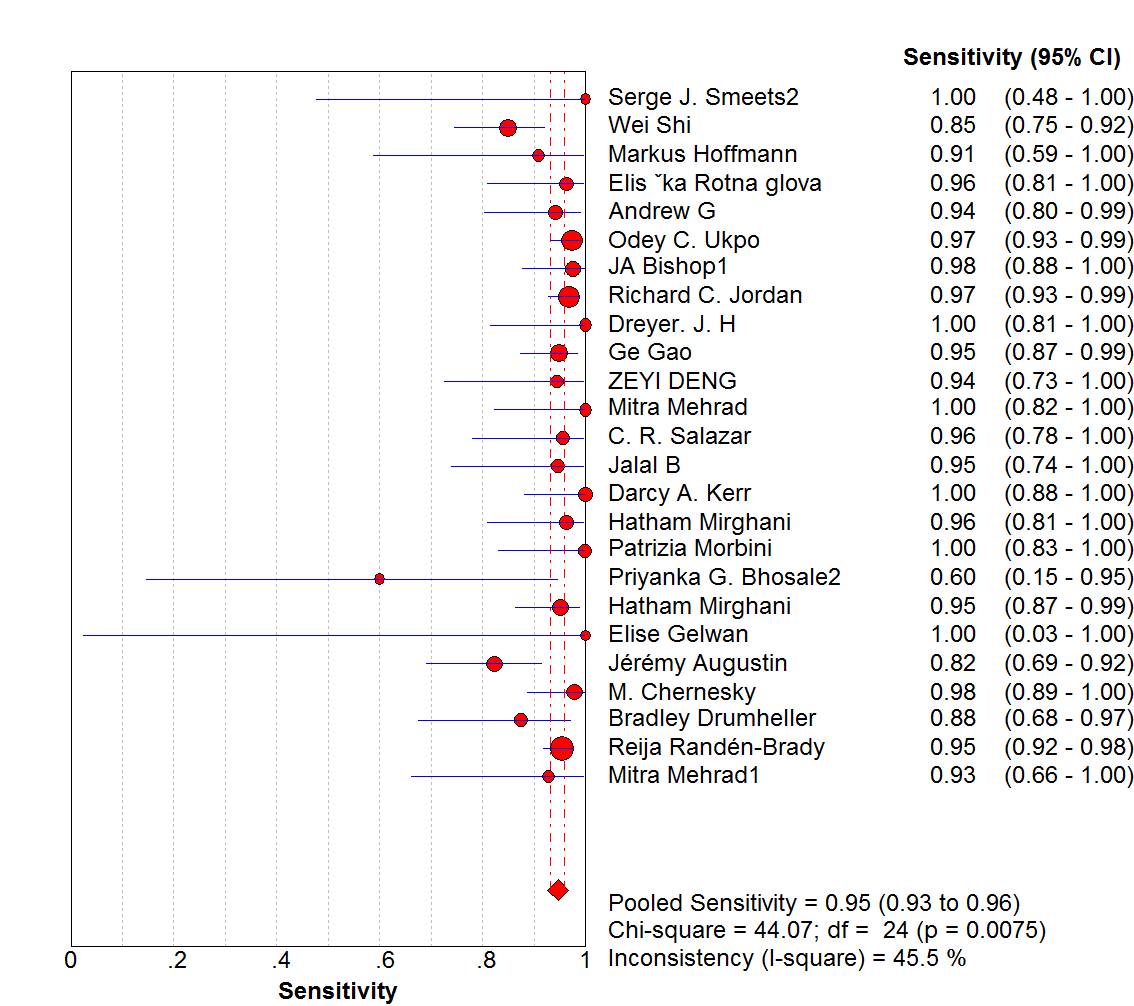

Supplement: Supplementary file 1 [file Data_Sheet_1.zip › Supplement file 2020.1.7/Supplementary file - figure/OP or no-OP/OP-sensitivity.jpg]

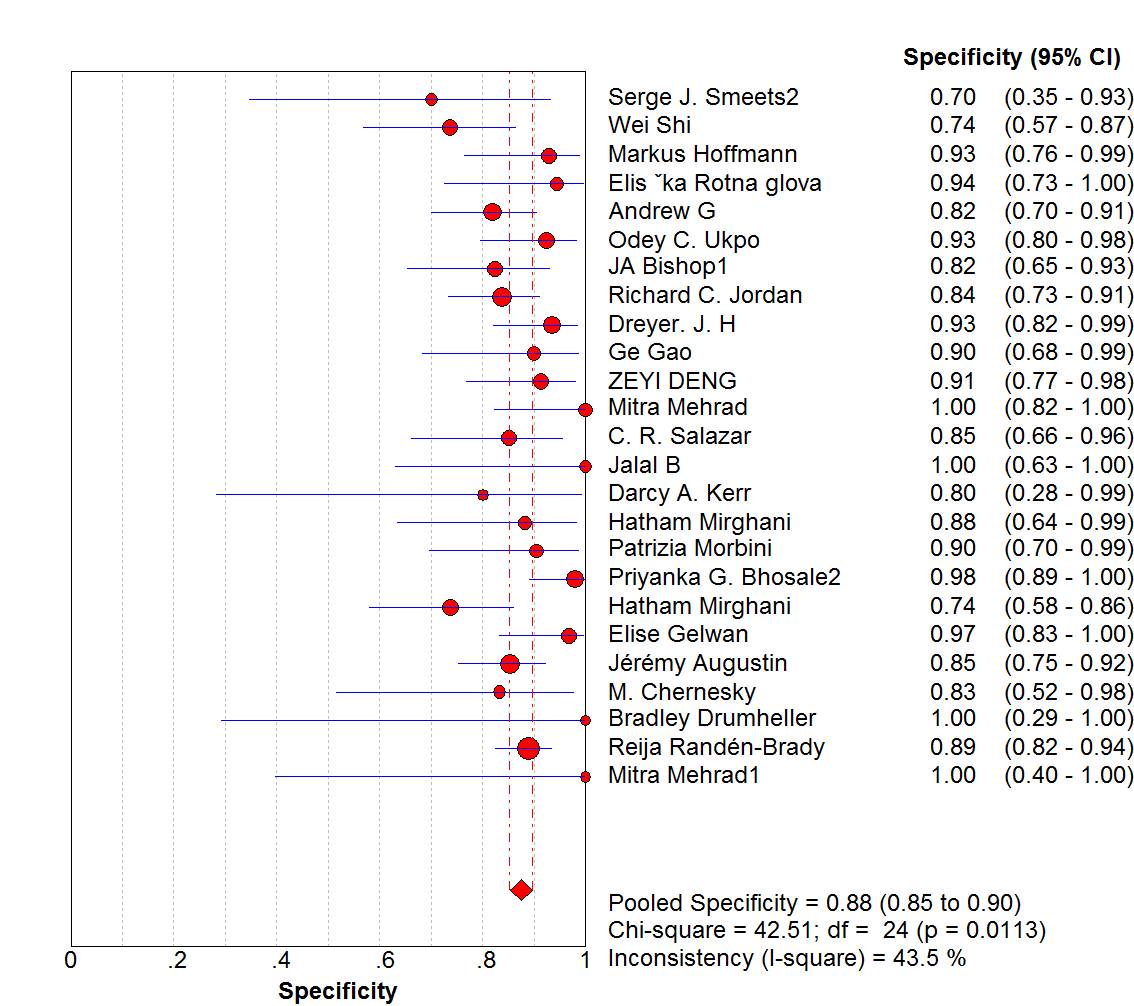

Supplement: Supplementary file 1 [file Data_Sheet_1.zip › Supplement file 2020.1.7/Supplementary file - figure/OP or no-OP/OP-specificity.jpg]

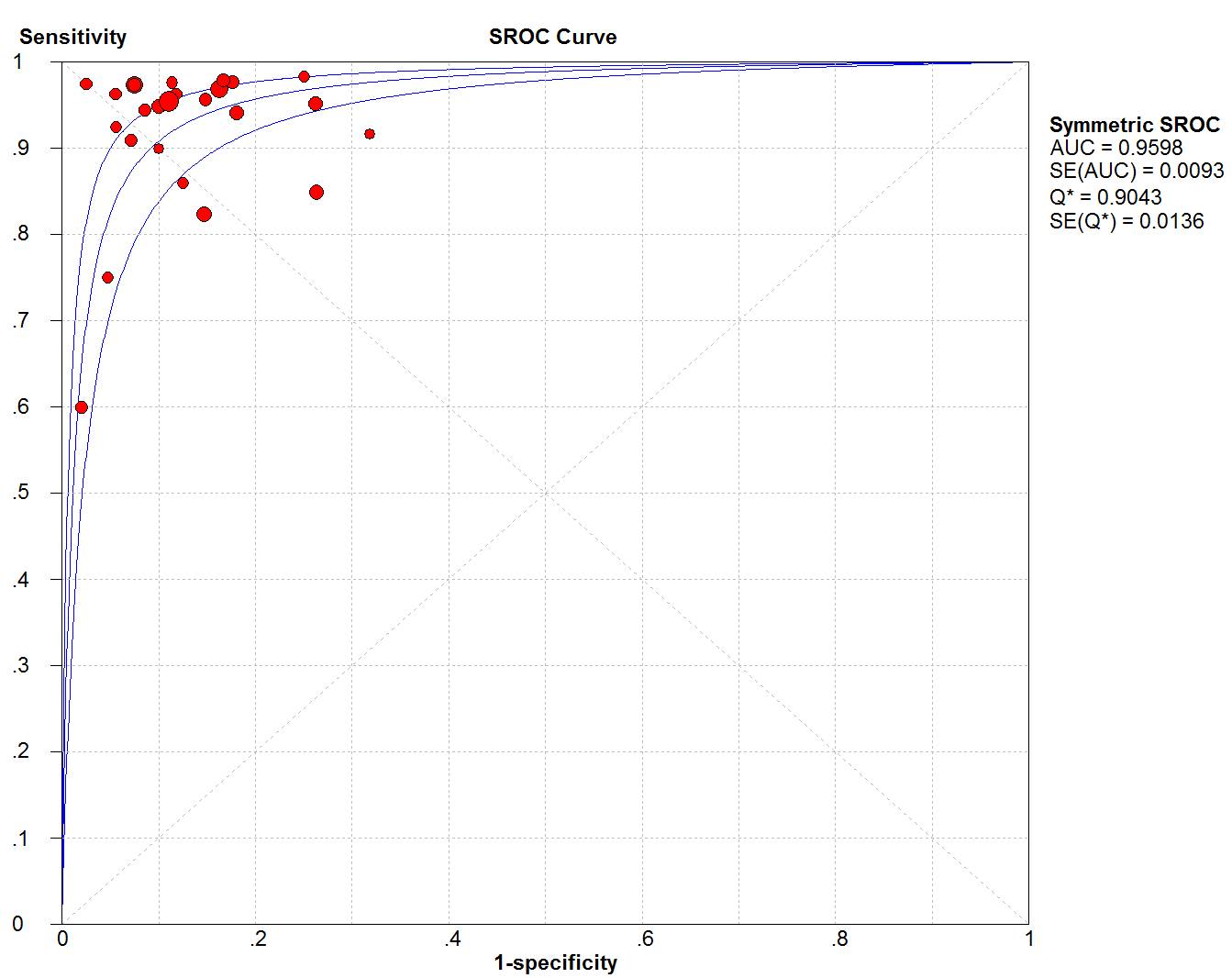

Supplement: Supplementary file 1 [file Data_Sheet_1.zip › Supplement file 2020.1.7/Supplementary file - figure/OP or no-OP/OP-SROC curve.jpg]
